# A Novel Role for *CSRP1* in a Lebanese Family with Congenital Cardiac Defects

**DOI:** 10.3389/fgene.2017.00217

**Published:** 2017-12-18

**Authors:** Amina Kamar, Akl C. Fahed, Kamel Shibbani, Nehme El-Hachem, Salim Bou-Slaiman, Mariam Arabi, Mazen Kurban, Jonathan G. Seidman, Christine E. Seidman, Rachid Haidar, Elias Baydoun, Georges Nemer, Fadi Bitar

**Affiliations:** ^1^Department of Biology, American University of Beirut, Beirut, Lebanon; ^2^Department of Genetics, Harvard Medical School, Boston, MA, United States; ^3^Department of Medicine, Massachusetts General Hospital, Boston, MA, United States; ^4^Division of Cardiology, Howard Hughes Medical Institute, Brigham and Women's Hospital, Boston, MA, United States; ^5^Department of Biochemistry and Molecular Genetics, American University of Beirut, Beirut, Lebanon; ^6^Faculty of Medicine, McGill University, Montreal, QC, Canada; ^7^Department of Pediatrics and Adolescent Medicine, American University of Beirut, Beirut, Lebanon; ^8^Department of Dermatology, American University of Beirut, Beirut, Lebanon; ^9^Department of Dermatology, Columbia University, New York, NY, United States; ^10^Department of Surgery, American University of Beirut, Beirut, Lebanon

**Keywords:** congenital heart disease, CSRP1, TRPS1, polydactyly

## Abstract

Despite an obvious role for consanguinity in congenital heart disease (CHD), most studies fail to document a monogenic model of inheritance except for few cases. We hereby describe a first-degree cousins consanguineous Lebanese family with 7 conceived children: 2 died *in utero* of unknown causes, 3 have CHD, and 4 have polydactyly. The aim of the study is to unveil the genetic variant(s) causing these phenotypes using next generation sequencing (NGS) technology. Targeted exome sequencing identified a heterozygous duplication in *CSRP1* which leads to a potential frameshift mutation at position 154 of the protein. This mutation is inherited from the father, and segregates only with the CHD phenotype. The *in vitro* characterization demonstrates that the mutation dramatically abrogates its transcriptional activity over cardiac promoters like *NPPA*. In addition, it differentially inhibits the physical association of CSRP1 with SRF, GATA4, and with the newly described partner herein TBX5. Whole exome sequencing failed to show any potential variant linked to polydactyly, but revealed a novel missense mutation in *TRPS1*. This mutation is inherited from the healthy mother, and segregating only with the cardiac phenotype. Both TRPS1 and CSRP1 physically interact, and the mutations in each abrogate their partnership. Our findings add fundamental knowledge into the molecular basis of CHD, and propose the di-genic model of inheritance as responsible for such malformations.

## Introduction

Congenital heart defects arise during pregnancy, and are subsequently the most prevalent birth defects worldwide (Hoffman and Kaplan, 2002). They affect chamber and valve formation and function leading to different phenotypes referred to as Congenital Heart Disease (CHD), the major cause of neonatal morbidity and mortality in humans. CHD accounts for one third of all main congenital defects with variable prevalence crosswise countries. In Lebanon, the incidence of infants born with CHD between 1980 and 1995 was 11.5 per 1,000 live births (Bitar et al., 1999), and twenty per cent of those patients were found to be from first degree cousin mating (Nabulsi et al., 2003). Although many studies have attempted to establish a relationship between CHD and consanguinity, the significance of this association and its precise nature is still unclear. So far, at least 50 human disease genes have been associated with CHD, however, a small set of developmental genes [for example, *NKX2-5* (MIM# 600584), *GATA4* (MIM# 600576) and *NOTCH1*] harbor the majority of these CHD-associated mutations (Fahed et al., 2013).

Mutations in genes encoding LIM domain proteins have been however, rarely associated to cardiac morphogenesis or CHD. The LIM domain contains a conserved double zinc finger motif that is evolutionary conserved and is found in a variety of proteins displaying distinct biological roles (Schmeichel and Beckerle, 1994). The LIM domains have been observed to act as a mediator of protein-protein interactions in the cytoplasm and the nucleus. These interactions with specific protein partners are now known to influence its subcellular localization and activity (Khurana et al., 2002; Kadrmas and Beckerle, 2004; Camarata et al., 2006). Many LIM proteins that were initially identified as cytoskeleton-associated proteins, such as members of the cysteine-rich protein (CRP) families, four-and-a-half LIM (FHL), PINCH and Zyxin are recognized to shuttle between the cytoplasm and nucleus of the cell to influence gene expression (Sadler et al., 1992; Schmeichel and Beckerle, 1994; Bianchi-Smiraglia et al., 2013). This dual localization is due to the presence of a putative nuclear targeting signal (KKYGPK) that has been identified in the glycine-rich regions of the CRPs. In humans, three CRP-family members (group two of LIM domain proteins) have been identified which are: CSRP1 (MIM# 123876), CSRP2, and CSRP3/MLP (Pomiès et al., 1997; Henderson et al., 2002; Weiskirchen and Gunther, 2003). CSRPs are small proteins, 22 kDa in size, and contain two functional LIM domains that are linked to glycine-rich repeat. CSRP family members play a role in terminal differentiation in vertebrate muscle development. CSRP1 and CSRP2 are prominent in smooth muscle while CSRP3 is expressed in striated muscle (Weiskirchen and Gunther, 2003). In embryonic development CSRP1 participates in the formation of heart, and its downregulation alters cardiac-committed mesodermal cell migration resulting in *cardia bifida* in zebrafish (Miyasaka et al., 2007). CSRP1 inhibition leads to irregular cell movement in convergent extension resulting in anomalies in midline structures. In adult mouse, CSRP1 is expressed in the smooth muscle cells of cardiac arteries. CSRP1 and CSRP2 were shown to function through coordinated docking of Serum-Response Factor (SRF) to the N-terminal LIM domain and GATA factors, specifically GATA4 and GATA6, to the C-terminal LIM domain (Lilly et al., 2001, 2010). The strong expression of many smooth muscle-differentiation markers is stimulated by this ternary complex of SRF–CSRP–GATA, whereas the pairwise combinations have much less impact on gene expression. In the cytoplasm, CSRPs that are associated with the actin cytoskeleton might function as sensors to assess the physiological status of the contractile machinery by interacting with α-actinin, and with the adhesion plaque LIM protein domain Zyxin (Sadler et al., 1992).

The interaction of CSRP proteins with GATA zinc finger transcription factors underscores their potential implication in CHD, since mutations in genes encoding all three cardiac enriched GATA proteins were shown to be associated with multiple forms of structural cardiac defects (Kassab et al., 2016 #3679; Nemer et al., 2006 #44). Besides GATA4, 5, and 6, an atypical GATA protein was shown to bind the specific GATA sequence on DNA and competes with the canonical GATA proteins to repress their activities (Momeni et al., 2000; Malik et al., 2001; Kunath et al., 2002). Besides GATA1-6, few proteins harbor a GATA-zinc finger motif in their structure. Amongst these, TRPS1 (Trichorhinophalangeal syndrome type I) contains nine putative zinc finger domains with the seventh finger representing the GATA-type while zinc fingers 8 and 9 reveal homology to a conserved domain of lymphoid transcription factors that belong to Ikaros family (Momeni et al., 2000; Malik et al., 2001). TRPS1 differs from other GATA proteins by its *in vitro* and *in vivo* activity as a sequence-specific transcriptional repressor rather than an activator since although it binds a GATA sequence, it fails to activate GATA transactivation reporter (Malik et al., 2001). Mutations in the *TRPS1* (MIM# 604386) is linked to the autosomal dominantly inherited TRP (tricho-rhino-phalangeal) syndrome which is characterized by skeletal and craniofacial malformations (Momeni et al., 2000; Kunath et al., 2002). Specifically, some of the major features include hip malformations, sparse scalp hair, bulbous tip of the nose, protruding ears, short stature, brachydactyly, and cone-shaped epiphyses in the phalanges (Momeni et al., 2000; Malik et al., 2001). Recent studies have shown that some patients with this syndrome display wide range of congenital cardiac defects including persistent foramen ovale (PFO), persistent ductus arteriosus (PDA), aortic stenosis, and left cardiac insufficiency (Verheij et al., 2009; Maas et al., 2015).

We have recently identified a large Lebanese family with CHD and polydactyly composed of the consanguineous marriage between two first-degree cousins. Out of the 7 conceived children, 2 died *in utero* at the ages of 6 and 9 months of unknown causes. Of the remaining 5 children, 3 have CHD (ventricular septal defect, atrial septal defect, and patent ductus arteriosus), and 4 have polydactyly (2 have both). We thus carried targeted and subsequently whole exome sequencing to unravel the genotype-phenotype relationship within this family. The targeted sequencing of 119 cardiac candidate genes, led to the identification of a novel heterozygous frameshift variant in *CSRP1* in all probands with cardiac defects. This variant is inherited from the unaffected father. Whole exome sequencing showed amongst other a potentially damaging missense varaint in *TRPS1* inherited from the unaffected mother. We aimed thus to study the effect of these variants on the protein function and structure in vitro, and our results suggest a digenic model of inheritance that would explain the occurrence of CHD in this family.

## Materials and methods

### Patient recruitment and clinical examination

The study was approved by the institutional review board (IRB) at the American University of Beirut (protocol number: Bioch.GN.01). All subjects gave written informed consent in accordance with the Declaration of Helsinki. Children and adolescents under age of 16 signed an assent form, and got their parents's written consents to be included in the study. Genetic analyses and return of genetic data were performed in accordance with protocols approved by the Partners Human Research Committee. A total of 20 individuals from the same family were enrolled. Standard clinical evaluation included a comprehensive physical exam, electrocardiography (ECG), and two-dimensional (2D) transthoracic echocardiography (TTE) with color Doppler. All affected members underwent karyotyping to assess chromosomal integrity.

### Genetic analysis

Peripheral venous blood was collected from all family members, and DNA extraction was performed using the Qiagen Blood-Midi kit (Qiagen Science Inc., Germantown, MD, USA), following the manufacturer's protocol. DNA quantification was performed using the NanoDrop (Thermo Fisher Scientific Inc., Waltham, MA, USA) at the molecular core facility at AUB. Targeted DNA sequencing was done at Harvard as previously described. One microgram of coded DNA samples from both parents, the proband, four of her siblings, and three of her cousins were shipped to Macrogen, South Korea (http://dna.macrogen.com/eng/) where exome sequencing was performed. Briefly, the samples were prepared according to an Agilent V6 SureSelect Target Enrichment Kit preparation guide. The SureSelect Target Enrichment workflow is solution-based system utilizing ultra-long—120 mer biotinylated cRNA baits—to capture regions of interest, enriching them out of a NGS genomic fragment library (Chen et al., 2015; #2881). The libraries were sequenced with an Illumina HiSeq 2000 sequencer. The Illumina technology utilizes a unique “bridged” amplification reaction that occurs on the surface of the flow cell. Sequencing-by-Synthesis utilizes four proprietary nucleotides possessing reversible fluorophore and termination properties (Bentley et al., 2008; #2882).

Primary analysis was done at Macrogen. Generated Fastq files were mapped to the reference genome using the Burros-Wheeler Alignment Tool (BWA), and the Genome Analysis Toolkit (GATK) was used for variants call, while the SnEff software was used to annotate the variants. Variant analysis was done using the Illumina variant call software, using a family based approach and comparing results to an in house 200 exomes database and to the available online exome and genome databases. Variants segregating with the phenotype (s) were submitted to the Leiden Operation Variome Database (http://www.lovd.nl/3.0/home) under screening ID# 00121860. Sanger sequencing was used to confirm the genotype of the *CSRP1* and *TRPS1* variants in all available family members. Briefly, amplification by polymerase chain reaction (PCR) was done using the Phusion polymerase high-fidelity master mix (F-548S) on a Pico machine (Finnzymes, Espo, Finland), and the amplicons were resolved on a 1.5% agarose gel. Gel purification was performed using the Gel Extraction kit following the manufacturer's protocol (peqGOLD Gel Extraction Kit, PeqLab, Erlangen, Germany). The purified bands were quantified using a NanoDrop (Thermo Fisher Scientific Inc., Waltham, MA) and examined by gel electrophoresis to ensure quality. DNA sequencing was carried out on an ABI 3500 machine at the molecular core facility at the American University of Beirut, followed by analysis using the data collection software from Applied Biosystems Inc. (Foster City, CA).

### Detection of copy number variants from WES

Read counts for all exons were extracted from clinical samples, following the computational approach proposed by Zhang et al. (2015). Briefly, the GRch38 version of the Human genome and a target region covering 50 MB of exons is used to extract counts from BAM (Binary alignment map) files for patients and controls, respectively. The resulting compressed ^*^tar.gz file is then submitted to the DeAnnCNV webserver which infers the CNV for each subject based on a previously described algorithm: the Global Parameter Hidden Markov Model (GPHMM) (Li et al., 2011). DeAnnCNV identifies a shared CNV pattern between patients and provides further annotation of the genes associated with these CNVs.

### Cell lines and plasmids

HEK293 cells (Human Embryonic Kidney cells) and Hela cells (Human cervical cancer cells) were cultured and maintained in Dulbecco's Modified Eagle Media (DMEM-Sigma, Cat#D0819) supplemented with 10% Fetal Bovine Serum (FBS-Sigma, Cat#F9665), 1% Penicillin/ Streptomycin (Biowest-Cat#L0022-100) and 1% sodium pyruvate (Sigma-Cat#S8636). Incubation of cells was carried out in a 5% CO_2_ humid atmosphere at 37°C.

Luciferase reporter plasmids PGL3-VEGF-luc, PGL2-NOS3-luc, and PXP2-NPPA-luc were constructed by ligation of PCR–amplified fragments from mouse VEGF, human NOS3 and rat NPPA promoters into eukaryotic luciferase expression vectors PGL3, PGL2, and pXP2 respectively. pCGN-HA-SRF, HA-tagged pCGN-GATA (4, 5, and 6) and pCGN-TBX5 were cloned into eukaryotic expression vector pCGN. pCMV3-HA-TRSP1 was from Sino Biological Inc. (Cat# HG15989-NY). Flag-tagged wild type human CSRP1 was generated by subcloning CSRP1 fragments into expression vector pCEP4 (Invitrogen). Flag-tagged p.E154Vfs^*^99 CSRP1 mutant was constructed through site-directed mutagenesis in which PCR amplified fragments harboring the p.E154Vfs^*^99 mutation were ligated into eukaryotic expression vector pCEP4. Site directed mutagenesis was also carried out to introduce p.R311S *TRPS1* mutation into the wild-type (WT) TRPS1. After the ligation of the resulting amplicons, transformation into XL-1 Blue competent bacteria was performed. Finally, the yielded plasmids were extracted and sequenced thereafter in order to confirm the incorporation of the mutations.

### Antibodies

Rabbit polyclonal antibody to CSRP1 Cat#:ab70010; Goat anti-mouse antibodies (HRP) Cat# ab6789; and goat anti-rabbit antibodies (HRP) Cat# ab97051 were purchased from Abcam. Mouse monoclonal antibody against flag-tag (OCTA-probe H-5) Cat# sc-166355 and rabbit polyclonal antibodies against HA-tag (HA-probe Y-11) Cat# sc-805 were from Santa Cruz. Mouse Biotinylated species-specific whole antibody (from donkey), Cat#: LRPN1001V and rabbit biotinylated species-specific whole antibody (from sheep); Cat# RPN1004V were from GE healthcare UK limited. Streptavidin Red full length Cat#ab136227 was from abcam. Goat anti-rabbit Alexa fluor 488 was from Invitrogen Cat#A11008.

### Transfection assays to assess CSRP1 target gene promoters

HEK293 cells were grown and maintained at subconfluence ~60% level in Dulbecco's modified Eagle's Medium (DMEM-Sigma) containing 10% fetal bovine serum (FBS). Transient transfections were performed with Polyethylenimine (Sigma). A series of Luciferase assays were performed by transient transfection in combination with pCEP4-Flag-WTCSRP1, pCEP4-Flag-p. E154Vfs^*^99 CSRP1,PCGN-HA-SRF,pCGN-HA-GATA4, pCGN-HA-GATA4,luciferase reporters (3.5 μg) and empty expression vectors, PCGN, to a balanced total of 1 μg of plasmids per 2 wells of the 12-well plate. The results were normalized the total protein concentration in each well, and were expressed as fold activation. Cotransfection experiments were performed in duplicates and repeated at least three times. Luciferase activity was normalized to baseline reporter gene activity as fold activation, with error bars representing SEM.

### Protein overexpression and western blotting

HEK293 cells were transiently transfected with epitope-tagged vectors pCGN-HA-GATA4, pCEP4-Flag-WTCSRP1, pCEP4-Flag-p.E154Vfs^*^99 CSRP1, pCEP4-HA-TBX5, pCGN-HA-GATA5, pCGN-HA-GATA6, PCGN-HA-SRF, pCMV3-HA-TRSP1, and pCMV3-HA-p.R311S TRPS1 using Polyethylenimine (Sigma). HEK293 cells were plated in 100 mm corning culture plates until subconfluence ~80% determined by green fluorescent protein (GFP) transfection assay. After 24 h, 20 μg of DNA and 35 μl PEI (transfection reagent) were added to an Eppendorf tube holding a total volume of 1 ml DMEM medium. The mixture was vortexed for 10 sec, incubated 20 min at RT, and then applied over the cells. Culture medium was changed after 3 h of transfection. Nuclear extracts from transfected HEK293 cells were obtained as previously described. For immunoblotting, 10 μg of nuclear extracted proteins were mixed with 5X Laemmli Buffer. The protein samples were boiled for 5 min and run on denaturing SDS-PAGE for about 1.5 h then transferred to a PVDF membrane (Amersham, UK) Cat#10600023. The membrane was blocked in 5%TBT (TBS-0.02% Tween 20) skimmed dry milk for 45 min at RT. The membrane was incubated with primary antibodies, anti-Flag or ant-HA (1:1,000) overnight at 4°C. On the second day, the membrane was washed three times with TBT and incubated with secondary anti-mouse or anti-rabbit-HRP (1:50,000) for 1 h at RT. Development was done using ECL™ Western Blotting Detection Reagents (Amersham, GE healthcare, Cat# RPN2106). The protein bands were visualized by Chemidoc MP imaging systemBio-Rad and quantified using Image J software.

### Co-immunoprecipitation

After detecting WT CSRP1, p.E154Vfs^*^99 CSRP1, GATA-4, -5, and -6, SRF, TBX5, and WT TRPS1 and p.R311S TRPS1 proteins by western blot, co-immunoprecipitation assay was done to assess the physical interaction between WT/ p.E154Vfs^*^99 CSRP1 (Flag-tagged), GATA-4, -5, and -6 (HA-tagged), SRF (HA-tagged), TBX5 (HA-tagged), and WT/p.R2311S TRPS1 (HA-tagged). About 5 μg of anti-rabbit HA (Santa Cruz) plus PBS (1x, 0.001% Tween 20) were incubated with Dynabeads® Protein G [size: 1 ml (30mg/ml) Novex by Life Technologies, Cat# 10003D] for 1 h at 4°C. 200 μg of total proteins (ten times the amount used in western blot) were incubated with antibodies and beads for 2 h at RT. Immunocomplexes were captured on magnet and washed three times with PBS 1X. Coimmunoprecipitated proteins were subjected to Western Immunoblot analysis as per regular protocol (anti-Flag, 1:1,000). PVDF membrane was stripped and probed with anti-HA antibody (Santa Cruz), 1:1,000. The protein bands were visualized by autoradiography.

### Immunostaining

Hela cells were grown onto 12-well costar culture plates on coverslips at subconfluence ~60% level and maintained in Dulbecco's modified Eagle's medium (DMEM) having 10% FBS. Transfections were done using polyethylenimine (PEI-Sigma). Five micrograms of DNA was diluted in 150 μl of serum free DMEM medium and 6 μl of PEI was added into an Eppendorf in a ratio of 1:3 DNA to PEI. Hela cells were fixed in 4% paraformaldehyde (Hummel et al., 1990). Nonspecific binding was blocked with 3% Bovine serum Albumin (BSA) in 0.2% PBS-Tween20 (PBT) and primary mouse monoclonal antibody (1:250) against flag-tag (OCTA-probe H-5) or rabbit polyclonal IgG (HA-probe Y-11) were applied overnight at 4°C. Secondary anti-mouse Biotinylated species-specific whole antibody (from donkey), GE healthcare UK limited, or secondary anti-Rabbit biotinylated species-specific whole antibody (from sheep); diluted 1:500 was added for 1 hour at room temperature. Cells were washed three times with PBT and incubated for 1 hour at room temperature with Streptavidin Texas Red full length (abcam, Cat#ab136227) diluted 1:500. Cells were washed three times with PBT and incubated with Hoechst staining for the nucleus, diluted 1:30 in water, for 15 minutes. The cells were then mounted on a rectangular slide containing an anti-fading agent DABCO (Sigma-Aldrich). The slides were examined using the Olympus BH-2 microscope at the molecular core facility in the faculty of medicine-AUB.

### Immunohistochemistry

Continuous sections which are 5 μm thick were prepared from each formalin-fixed, paraffin embedded tissue. Immunohistochemical staining was performed to evaluate the expression of CSRP1. All sections on the slides were dewaxed and rehydrated with xylene and graded alcohol, then dripped 3% hydrogen peroxide on them to quench endogenous peroxidase. Afterwards, high-temperature antigen retrieval was carried out in citrate buffer (pH 6.0) in a microwave oven to enhance immunoreactivity, followed by 3% BSA (Amresco Life science, Cat#0332-100G) in 0.2% PBT to reduce the non-specific bindings. Primary rabbit-antibody against CSRP1 (ab70010, abcam, 1:100) were applied to the sections respectively and incubated overnight at 4°C. Subsequently, slides were incubated with 1:250 secondary anti-rabbit Biotinylated species-specific whole antibody (from donkey), (GE healthcare UK limited, Cat# RPN1004V) and 1:250 streptavidin–peroxidase conjugate, and antibody-specific binding was visualized with 3,3-diaminobenzidine solution (DAB –Sigma, Cat#D3939-1SET). Lastly, slides were counterstained with Methyl Green and mounted. PBS was used as a negative control by replacement of the relevant primary antibody.

### Statistical analysis

The significance of luciferase assay was studied using Paired Students' *T*-test. The results are presented as fold activation and the values are Mean ± *SD* (standard deviation). Each experiment was performed in duplicates and repeated three times (*n* = 3). The significance is defined as ^*^p < 0.05 or ^**^p < 0.01.

## Results

**Figure 1:**
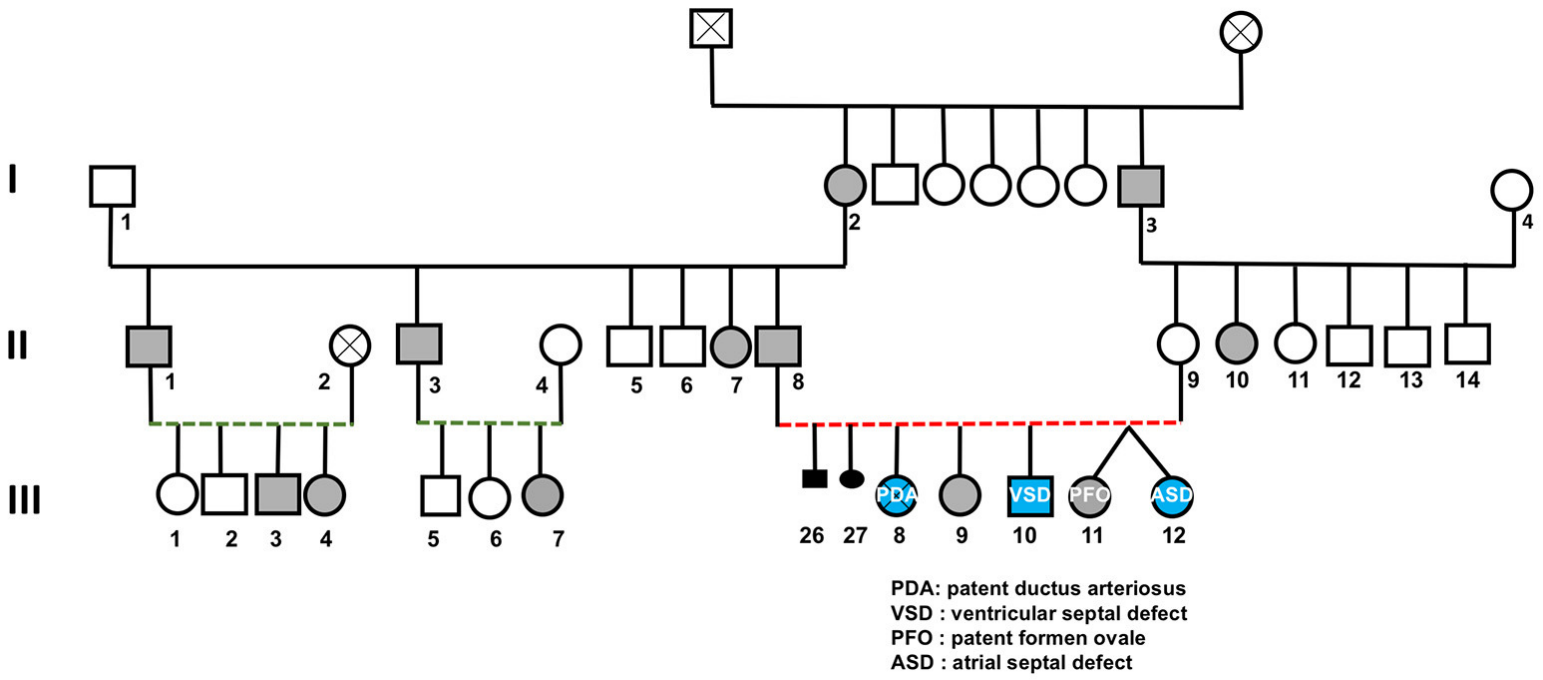
Congenital heart and limb deformities in a large consanguineous Lebanese family. The pedigree shows a three generations' family (roman numbers) with inherited polydactyly (gray symbols) or congenital heart defects amongst its members (Arabic numbers). Red and green dotted lines indicate first and second degree cousin marriages respectively.

A CHD consanguineous multiplex family with CHD and polydactyly was recruited as part of the Congenital Heart Disease Genetics Program at the American University of Beirut (Figure 1). The indexed-patient (III-8, Figure 1) presented to the AUB-MC Children's Heart center shortly after birth with a patent ductus arteriosus (PDA) and bilateral postaxial polydactyly on both hands and feet: the PDA was closed using an Amplatzer™ device. The girl passed away at 7 years of age following a severe lung infection. Her brother (III-10) presented with a small perimembraneous ventricular septal defect (VSD), lower extremity bilateral postaxial polydactyly, right hand syndactyly with postaxial finger formation, and left hand postaxial polydactyly with syndactyly in the 4^th^ and 5^th^ digits. Further examination of the core family (Figure 1) showed that the father (II-8) has bilateral postaxial polydactyly on feet and hands while the mother (II-9) is phenotypically normal (Figure 1). Both parents and the unaffected child (III-9) were confirmed upon echocardiography to be CHD free. The parents are first degree cousins, and had recently monozygotic twin-girls; one of them with a patent foramen ovale (PFO) and left-hand postaxial polydactyly (III-11), while the other (III-12) has only an atrial septal defect (ASD) (Figure 1).

### CHD targeted sequencing: the *CSRP1* variant

**Figure 2:**
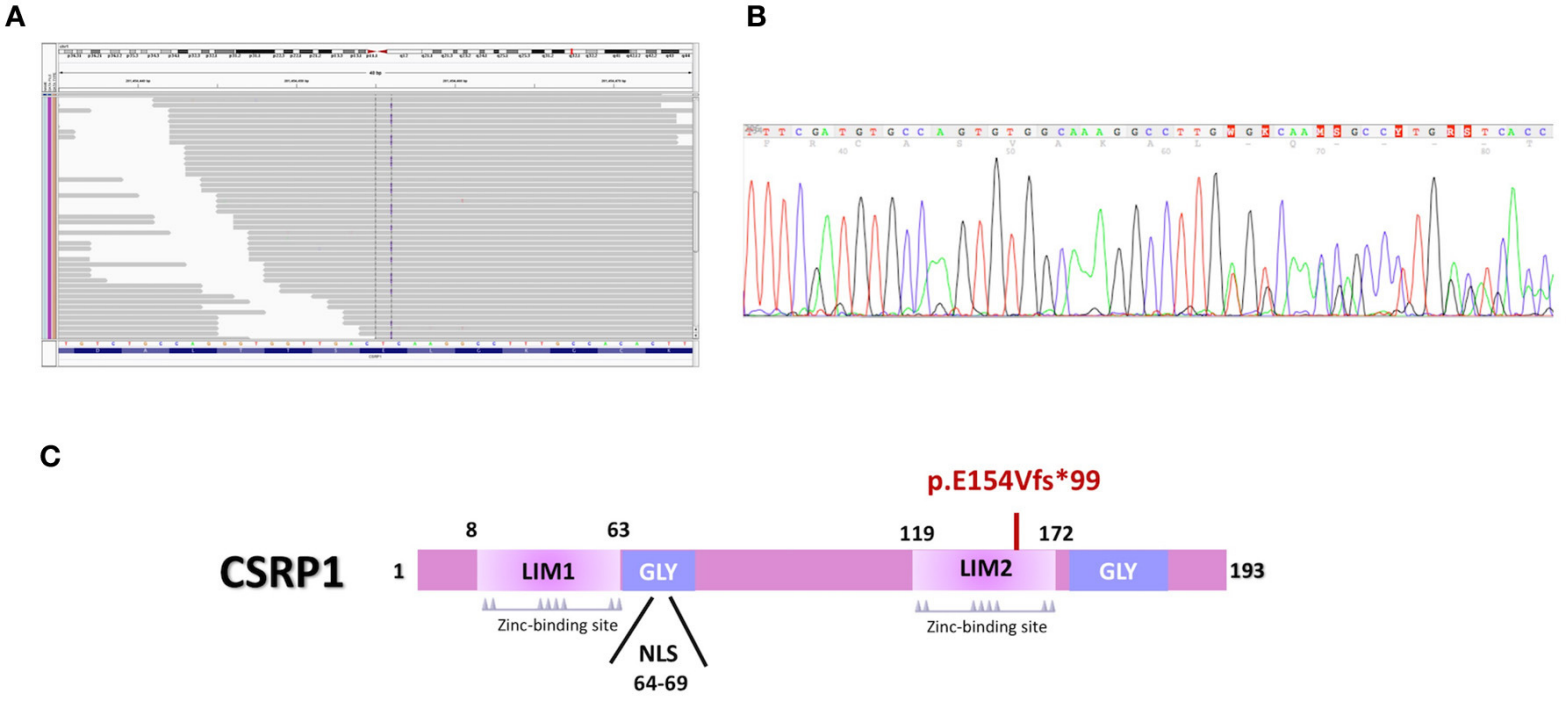
The *CSRP1* variant alters the structure of the protein. **(A)** Integrative genomics viewer (IGV) visualization of the targeted exome sequencing shows an insertion (Blue line) in Csrp1 gene. **(B)** Sanger Sequencing of the *CSRP1* gene confirmed the 14 nucleotides duplication (TGTGGCAAAGGCCT) in exon 5. **(C)** Schematic representation of the mutation that abrogates the second LIM domain of the protein.

Targeted sequencing of 119 genes implicated in CHD for probands III-8 and III-10 showed that none of these genes harbor shared rare (MAF < 1%) damaging variants except for the *CSRP1* gene (Supplementary Table 1). The variant [NM_004078.2:c.447_460dupTGGCAAAGGCCTTG] is an insertion of a segment of 14 nucleotides chr1:20145445-T>TCAAGGCCTTTGCCA that leads to a frameshift mutation with an extended C-terminal domain of the protein p.E154Vfs^*^99 (Figure 2A). The variant was shared by both probands and was neither present in the Genome Aggregation Database (gnomAD), nor in 200 Lebanese patients with CHD screened using the same approach. The variant was confirmed by Sanger sequencing (Figure 2B). Genotype-phenotype analysis across the extended pedigree did not support any role for *CSRP1* in the polydactyly phenotype, but suggested a potential role in the cardiac phenotype observed in the core family since the third affected child with ASD III-12 carried the variant, whereas proband III-9 who has no cardiac phenotypes did not (Table 1). The variant is inherited from the father who has a normal heart on echocardiography. This suggested that this variant could be involved in the cardiac phenotype observed in the family. Indeed, we carried out a Barnard's test for 2 × 2 contingency table and it showed a *p*-value of 0.021. This was corroborated by a Fisher's exact test yielding a p-value of 0.0476; both suggesting a significant association of the *CSRP1* variant with the CHD phenotype.

**Table 1:** Genotype of Family Members for CSRP1 and TRPS1 variants.

**Family member**	*CSRP1* (p.E154Vfs^*^99)	***TRSP1* (p.R311S)**
I-1,2,3,4	N/A	N/A
II-1	−/−	−/−
II-2,3,4,5,6,7	N/A	N/A
II-8	+/−	−/−
II-9	−/−	+/−
II-10	−/−	−/−
II-11,12,13,14	N/A	N/A
III-1,2	N/A	N/A
III-3,4	−/−	−/−
III-5,6,7	N/A	N/A
III-8	+/−	+/−
III-9	−/−	−/−
III-10	+/−	+/−
III-11	+/−	+/−
III-12	+/−	+/−

We thus, retrieved the coding sequence of the human *CSRP1*. Our analysis indicates that the duplication of 14 nucleotides in exon 5 (Figure 2A) leads to a reading frameshift that disrupts the 2nd LIM domain and extends the C-terminal domain of the protein (NP_001180500) (Figure 2C). The mutated protein harbors 253 amino acids instead of 193 suggesting a potential conformational, structural, and functional change.

### Cardiac expression and cellular localization of CSRP1

Before characterizing the impact of the mutation on the protein cellular localization and transcriptional activity, we did look at the expression of the protein during heart development in mice to correlate it with the phenotype(s) observed in the affected individuals. Results of immuno-staining showed a strong expression of the protein in the heart at all stages of development starting as early as E12.5, and onwards with a strong nuclear expression in all cardiac compartments, but not in the valves (Supplementary Figure 1). Of note the absence of the expression of the protein in the endocardial cells, and the progressive increase in the cytoplasmic localization of the protein whereby the newborn hearts of mice have mainly cytoplasmic CSRP1.

### Characterization of the transcriptional activity of the CSRP1 mutated protein

In order to assess the impact of the p.E154Vfs^*^99 mutation on the structural and functional properties of CSRP1, site-directed mutagenesis was carried out on the coding region of the human CSRP1 cDNA subcloned into a Flag-tagged plasmid. Both plasmids were sequenced before transiently expressing them into Hela and HEK293 cells. The wild type and mutated proteins were equally produced as assessed by western blot analysis of nuclear proteins extracted from these cells (Supplementary Figure 2A). Cellular localization was assessed in Hela cells which showed that both the wild type and mutated proteins are present in both the nuclei and cytoplasm of the transfected cells with no substantial differences (Supplementary Figure 2B).

**Figure 3:**
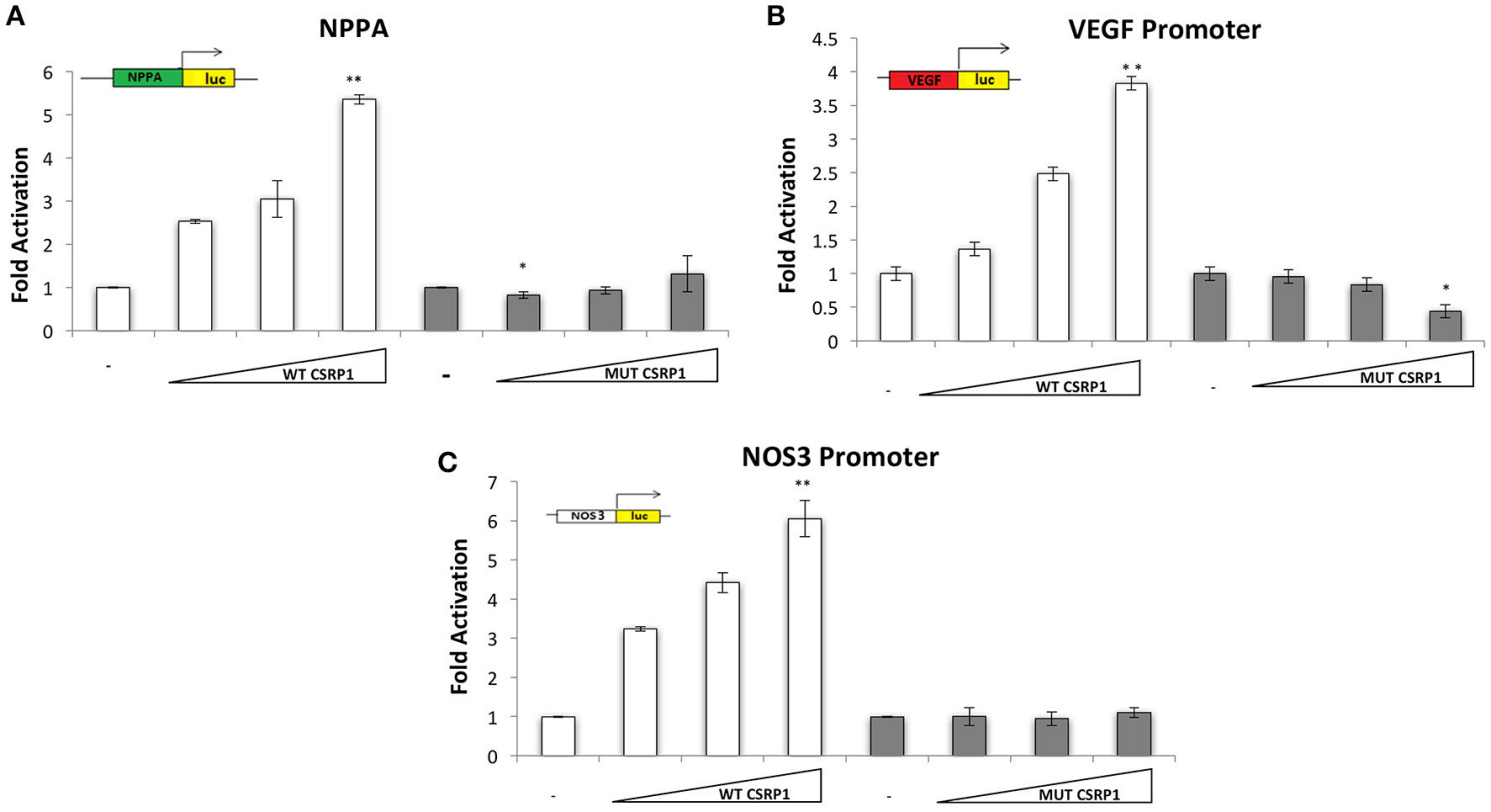
Transcriptional activity of WT and/or MUT CSRP1. **(A–C)** WT or MUT CSRP1 was transiently transfected along with 3.5 μg of NPPA, VEGF, and NOS3-luciferase promoters respectively in HEK293 cells. Relative luciferase activities were presented as fold changes. The data represent the means of 3 independent experiments done in duplicates and the values are ± SE. *P*-value was assessed used Students' *T*-test. Significance *p* < 0.01 is indicated by an (^**^) while *p* < 0.05 is indicated by (^*^); significance is tested relative to control. The triangle represents an increasing dose of the WT and MUT CSRP1 (200, 400, and 600 ng, respectively).

Although CSRP1 is predicted to act as a transcription factor, no previous studies have shown a direct activity of this protein on promoter regions of genes. In order to assess the effect of p.E154Vfs^*^99 mutation on the function of CSRP1, HEK293 cells were transiently transfected with increasing concentrations of the plasmid encoding either the wild type or mutant CSRP1 along with a fixed dose of either one of the following cardiac and/or vascular enriched promoters fused to Luciferase: NPPA, VEGF, and NOS3. While the wild type protein was able to activate the promoters in a dose-dependent manner reaching up to 6-fold over the NOS3 promoter, the mutation completely abolished this activity (Figure 3).

The weak transactivation properties of the CSRP1 protein coupled to the lack of a *bona fide* binding site for this class of LIM proteins prompted us to look at the effect of the mutation on its interacting partners.

### The p.E154Vfs^*^99 mutation alters the physical and functional interaction between CSRP1 and SRF

CRSP1 was previously shown to be recruited preferentially by the serum response factor (SRF) protein to promoter regions of target genes involved in smooth muscle cells differentiation (Chang et al., 2003). We thus assessed the effect of the mutation on this interaction by co-immunoprecipitation assays on HEK293 cells transiently overexpressing both proteins. The results showed that the mutation drastically inhibited the CSRP1/SRF interaction by 82% (Supplementary Figure 3A). The functional interaction was subsequently tested in co-transfection assays which show that both CSRP1 and SRF can synergistically activate the NPPA, VEGF, and NOS3 promoters up to 100-, 22.3-, and 14-folds, respectively (Supplementary Figure 3B), and that the mutation completely inhibits this synergy over the VEGF, and NOS3 promoters while drastically inhibiting it by 50% over the NPPA promoter (Supplementary Figures 3C,D).

### CSRP1/GATA4 interaction altered by the p.E154Vfs^*^99 CSRP1 variant

**Figure 4:**
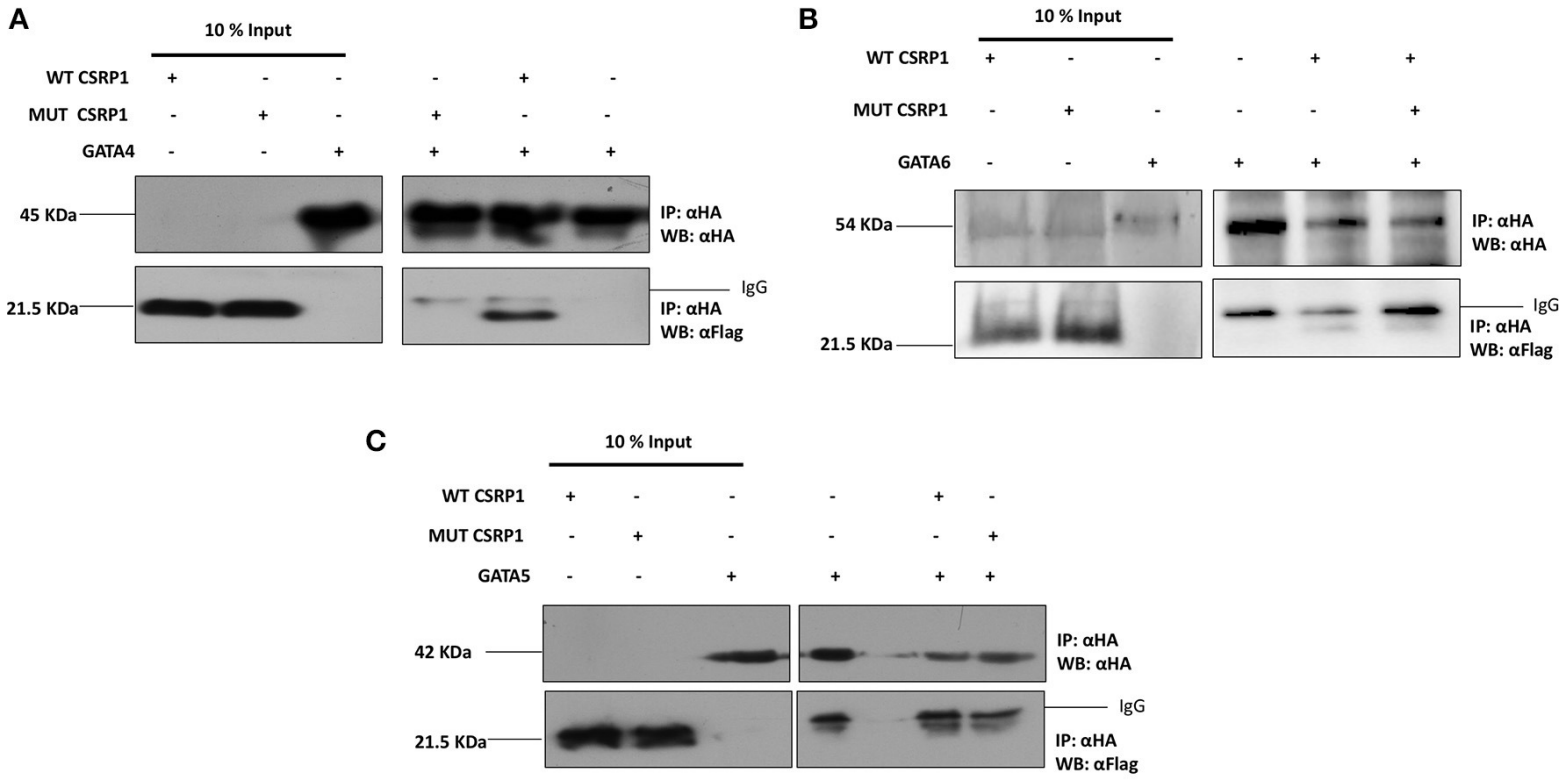
Selective inhibition of the GATA4 physical interaction with CSRP1 by the p.E154Vfs^*^99 mutation. **(A–C)** The amount of the proteins used for immunoprecipitation was ten times that used in western blot. Nuclear lysates of CSRP1 protein were immunoprecipitated with anti-HA antibody and CSRP1 proteins were detected using anti-Flag antibody (arrows). After membrane stripping, subsequent western blot analysis using anti-HA was performed to detect GATA4, 5, and 6 proteins (arrowheads).

GATA transcription factors and LIM-domain proteins (including CSRP1) have comparable zinc finger motifs through which they heterodimerize (Chang et al., 2003). Thus, we assessed the effect of the mutation by co-immunoprecipitation assay on HEK293 cells transiently expressing WT CSRP1, p.E154Vfs^*^99 variant, and GATA4, or GATA5, or GATA6. Our results show that only GATA4 and GATA6 readily interact with CSRP1 while GATA5 does not (Figure 4). More importantly the CSRP1 variant dramatically inhibited the GATA4/CSRP1 interaction up to 85% but has a mild effect on the CSRP1/GATA6 interaction which increased by ~15% (Figures 4A,B). Concurrently, the functional interaction between GATA4 and CSRP1 was also tested in co-transfection assays. The synergy between the two proteins was completely lost on the cardiac enriched promoters NPPA, VEGF, and NOS3 promoters going down from 22-fold to 5 (Supplementary Figure 4).

### TBX5 is a novel partner for CSRP1

**Figure 5:**
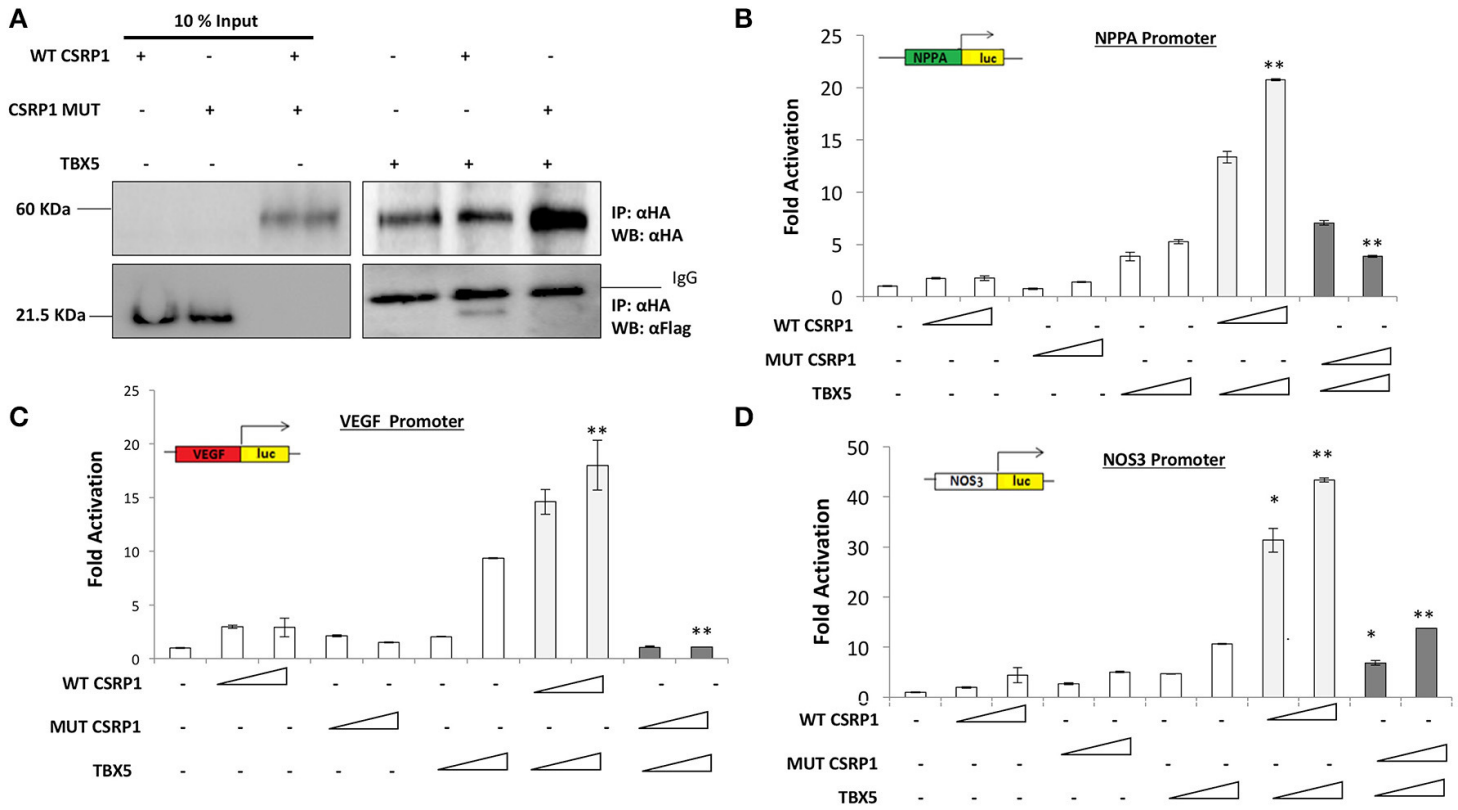
The effect of p.E154Vfs^*^99 mutation on the physical and functional interaction between CSRP1 and TBX5. **(A)** Physical interaction between HA-tagged TBX5 and Flag-tagged CSRP1 (WT and MUT) is demonstrated in the lanes of the right panel The amount of the proteins used for immunoprecipitation was 10 times that used in western blot. Nuclear lysates of CSRP1 protein was immunoprecipitated with anti-HA antibody and CSRP1 proteins were detected using anti-Flag antibody. After membrane stripping, subsequent western blot analysis using anti-HA was performed to detect TBX5 protein. **(B–D)** Transcriptional activity of WT and/or MUT CSRP1 along with TBX5. WT or MUT CSRP1 were transiently cotransfected with TBX5 along with NPPA, VEGF, and NOS3-luciferase promoters respectively in HEK293 cells. Relative luciferase activities were presented as fold changes. The data represent the means of 3 independent experiments done in duplicates and the values are ± SE. *P*-value was assessed used Students' *T*-test. Significance *p* < 0.01 is indicated by (^**^), while *p* < 0.05 is indicated by (^*^); significance of synergy for WT is tested relative to the sum of individual activations, while that of mutant is tested relative to synergy The triangle represents an increasing dose of the WT and MUT CSRP1 (400 and 600 ng, respectively) and TBX5 (200 and 400 ng).

The T-Box transcription factor, TBX5, is involved in vertebrate cardiac and limb development and mutation in this gene cause amongst others cardiac septal malformation, similar to the ones observed in our family. Moreover, it was shown that TBX5 interacts with the chicken and zebrafish LMP4, a member of PDZ-LIM proteins (Camarata et al., 2006). Thus, we were interested in assessing a potential interaction between CSRP1 and TBX5. Co-immunoprecipitation results revealed that TBX5 is a strong physical partner of CSRP1 (Figure 5A). Interestingly, the results of co-immunoprecipitation assays of HEK293cells overexpressing TBX5 and the variant revealed that the mutation decreased the interaction by up to 55.3%. We also assessed the functional interaction between CSRP1 and TBX5 by transient co-transfection assays in HEK293. The results showed a synergistic activation of these promoters reaching up to 40-fold on NOS3 promoter (Figure 5B); however, this synergy was completely abolished by the mutation in CSRP1 (Figures 5C,D).

### Exome sequencing: a novel TRPS1 variant

Since we did not find variations in *CSRP1* partners including *SRF, GATA4*, and *TBX5* to explain the genotype of the phenotypically normal individual II.8 (Table 1), we decided to carry on whole-exome sequencing (WES) on selected members of the family (II.8, II.9, III.1, III.2, III.8, III.9, III.10, III.11, and III.12). The raw data results showed an average total yield of ~6 × 10^9^ bp reads, with an average throughput depth of 87. Quality control analysis with the FastQC software showed an average of 90.6% coverage of target regions with more than 20 reads. From a total number of around 70,000 single nucleotide polymorphisms (SNPs) and 6,000 insertions/deletions (Indels), we only analyzed those occurring in the coding regions of the genes. We started by filtering out all variants with a minor allele frequency (MAF) >5%, and we kept common inherited variants amongst affected individuals with the cardiac and/or polydactyly phenotype (Supplementary Table 2). We first assumed an unbiased recessive model of inheritance for the cardiac phenotype (Figure 1), but we have failed to find a common recessive variant for individuals III8-10, -12 (Figure 1). We then moved to test the hypothesis of a common autosomal dominant variant shared between members affected with polydactyly alone as per the analysis of the phenotypes using the same stringent approach (Figure 1). We did not identify any rare damaging variant in previously documented polydactyly associated genes (Supplementary Table 3). Furthermore, there were no single variants common to all polydactyly affected individuals and not found in the rest of the healthy individuals from within the family and/or in the 200 in-house Lebanese exomes. This prompted us finally to investigate the potential implication of CNVs in both cardiac and limb defects independently using the DeAnnCNV webserver. The results did not yield any such significant CNV shared among patients as compared to controls.

**Figure 6:**
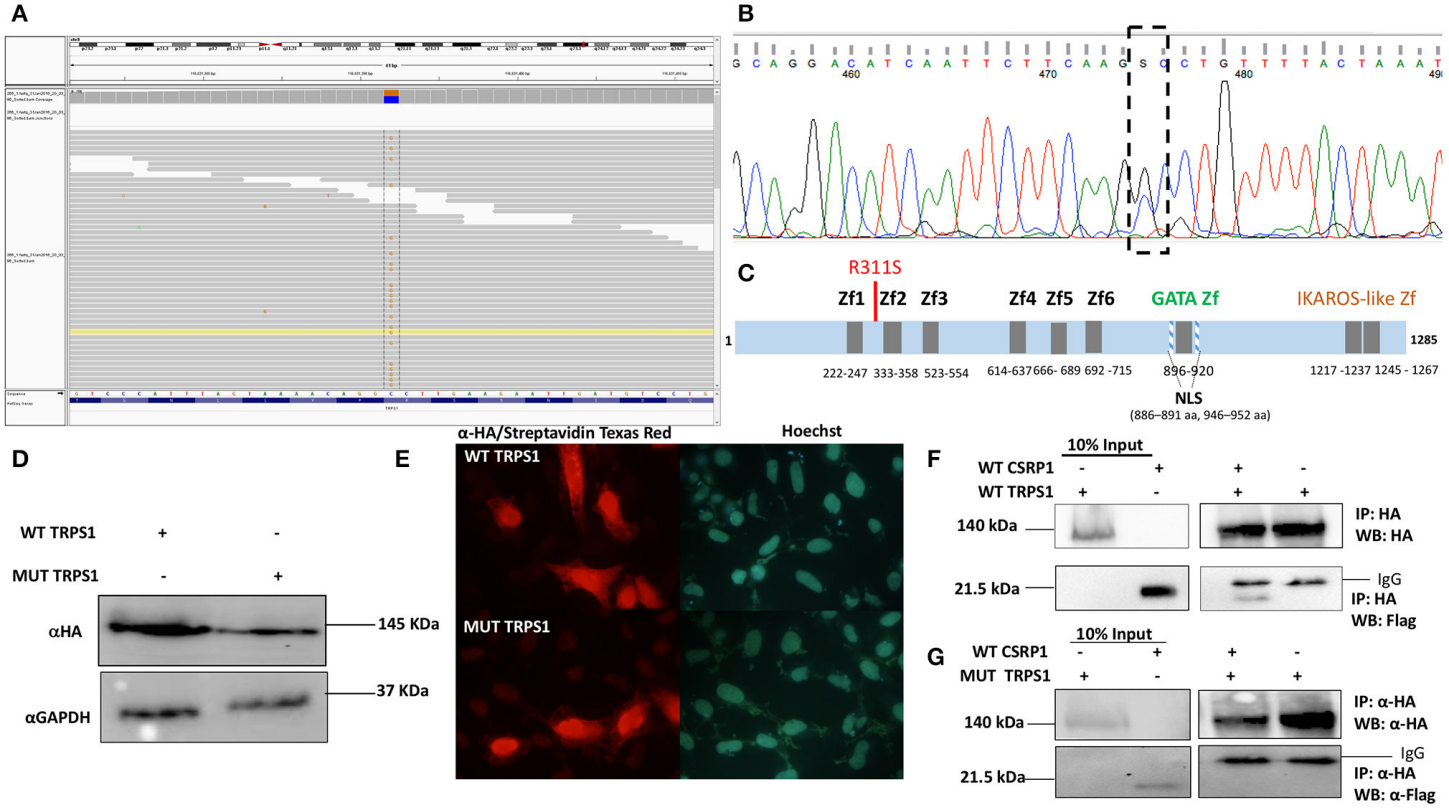
The Physical interaction between TRPS1 and CSRP1. **(A)**Whole-exome sequencing shows a point mutation [NM_014112.4:c.933G>C] in Trsp1 gene. **(B)** Sanger sequencing of *TRPS1* gene confirms the point mutation G>C in exon 8. **(C)** Schematic Representation of the TRPS1 protein with the different domains (Zf, Zinc finger; NLS, Nuclear Localization Signal), and the position of the mutation. **(D)** Nuclear extracts from transfected HEK293 cells with either WT TRPS1 or MUT TRPS1 were resolved on an SDS-PAGE. Immunoblotting using anti-HA antibody showed equal amounts of expressed proteins at 20 μg. Anti-GAPDH was used as a loading control. **(E)** Immunofluorescence of Hela cells transfected with WT TRPS1 and MUT TRPS1 plasmids. The localization of TRPS1 was visualized using anti-HA antibody followed by biotinylated anti-Rabbit antibody and then Streptavidin Texas Red for both WT TRPS1 and MUT TRPS1. Nuclei were stained blue with the Hoechst 33342 dye. TRPS1 (WT or MUT) showed cytoplasmic and nuclear localization (Red color). **(F)** Physical interaction between HA-tagged TRPS1 and Flag-tagged CSRP1 is demonstrated in the lanes of the right panel. The amount of the proteins used for immunoprecipitation was ten times that used in western blot. Nuclear lysates of CSRP1 protein was immunoprecipitated with anti-HA antibody and WT CSRP1 protein was detected using anti-Flag antibody. **(G)** This interaction is lost when using the MUT TRPS1 protein instead of the WT.

We then interrogated the digenic hypothetical model for CHD inheritance in the family by identifying rare damaging variants inherited from the mother (II.9) to the affected CHD probands. Using this approach with yet the same filtering stringency in addition to excluding any variant found in the in-house Lebanese exome database, we identified a list of 54 variants (Supplementary Table 4) amongst the individuals with cardiac defects (III-8, -9, -12 Figure 1) that are inherited from the mother (II-9, Figure 1). Amongst these, a non-synonymous mutation in exon 8 of the *TRPS1* gene [NM_014112.4:c.933G>C] was considered to be potentially implicated in the defect because of the previously documented expression and function of this gene in the heart, while other variants were within coding regions of genes not implicated in heart development and diseases. We confirmed by Sanger sequencing the TRPS1 genotyping of all available members of the family (Table 1). Structurally, this variation leads to a missense p.R311S mutation (ENST00000519076) at the level of the protein (Figures 6A–C). *In silico* analysis shows that the p.R2311S variant could have a probably damaging or neutral effect on the protein function, depending on the software used SIFT (http://sift.jcvi.org), Polyphen2 (http://genetics.bwh.harvard.edu/pph2/), Provean (http://provean.jcvi.org/index.php), and MutationTaster (http://www.mutationtaster.org). The variant is not found in the gnomAD database though highly covered both in the exome and genome databases (AF = 0). We thus hypothesized that a combinatorial role for the two variants in *CSRP1* and *TRPS1* could explain the CHD phenotype.

### Role of the digenic CSRP1/TRPS1 variants

In order to assess the impact of the p.R311S variant on the functional properties of TRPS1, site-directed mutagenesis was carried out on the coding region of the human TRPS1 cDNA sub-cloned into an HA-tagged plasmid. Both plasmids were sequenced before transiently expressing them into Hela and HEK293 cells. The wild type and mutated proteins were equally produced as assessed by western blot analysis of nuclear proteins extracted from these cells (Figure 6D). Cellular localization was assessed in Hela cells which showed that both proteins are present in the nuclei and cytoplasm of the transfected cells with no substantial differences (Figure 6E). Functional luciferase assays on the three cardiac enriched promoters did not show any significant transcriptional activity (data not shown), prompting us to study the direct interaction between CSRP1 and TRPS1. Co-immunoprecipitation assays from HEK293 cells overexpressing both proteins show a relatively stable interaction between the two (Figures 6F,G). Both mutations in *CSRP1* and *TRPS1* totally inhibit the interaction with the obligate partner (Figure 6, and data not shown), proving that both variants are deleterious.

## Discussion

We used both targeted exome and whole exome sequencing to unravel the genetic factors responsible for both cardiac malformations and polydactyly in a large Lebanese family with high consanguinity. We showed that despite the high consanguinity between members of the family, there were no homozygous mutations that could account for either or both phenotypes in the annotated genes. In addition, we found that both phenotypes are not linked, and that the cardiac phenotype is associated with a novel mutation in the gene encoding CSRP1. This is the second gene encoding a LIM protein shown to be implicated in CHD after ISL1 (Stevens et al., 2010; Luo et al., 2014). In addition, we interrogated the penetrance of this mutation by searching for potential modifiers. Our results unraveled a novel di-genic interaction between *CSRP1* and *TRPS1* which encodes a GATA-like zinc finger protein.

### *CSRP1*: a specific integrator of cardiac-enriched transcription factors

To the best of our knowledge, this is the first *CSRP1* variant identified in CHD patients or in any other cardiovascular diseases. The p.E154Vfs^*^99 mutation did not affect the cellular localization of the protein; however, it affected the structure and function of CSRP1 protein. The disruption of the protein physical interactions with its partners and the inhibition of the transcriptional activity on several cardiac enriched promoters suggest the severity of this mutation which affects the cardiac phenotypic outcome in the family. Our *in silico* analysis showed that this mutation is disrupting the second LIM domain at the C-terminal of the protein. The LIM domain has been shown to be implicated in protein-protein interactions (Arber et al., 1994; Schmeichel and Beckerle, 1994). Since the mutation lies in an essential domain of the protein, we suggested a conformational, structural and functional change at the protein level. This was corroborated by the obstruction of the physical interaction between CSRP1 and its partner SRF, a transcription factor mandatory for the appearance of cardiac mesoderm during embryonic mouse development and an essential partner of cardiac GATA4 (Wang et al., 2002; Small and Krieg, 2003). Similarly, the p.E154Vfs^*^99 has abrogated the interaction between CSRP1 and GATA4 which is also a vital transcription factor in the early and late heart development such as valve formation and cardiac septation (Molkentin et al., 1997). Dominant *GATA4* mutations cause severe CHD including atrial and atrioventricular septal defects (ASDs and AVSDs) as well as Tetralogy of Fallot (Garg et al., 2003; Nemer et al., 2006). This role of GATA4 in multiple forms of CHD is reminiscent of our findings for CSRP1 in this particular family, and could be explained by the broad yet timely expression pattern of the proteins during heart development. Indeed, we showed that CSRP1 is expressed in all cardiac compartments, and it was previously shown that a Cre-driven CSRP1 enhancer is highly expressed in the outflow tract, and both in the mesenchymal cells as well as cardiomyocytes supporting a role for CSRP1 in cardiac development (Snider et al., 2008). This specific expression in the heart would support a potential role for the CSRP1 variant in the different cardiac phenotypes observed in our family, but the fact that one normal individual carrying it stands against this conclusion. Interestingly, we showed that the mutation in CSRP1 did not affect its interaction with GATA5 and/or GATA6 suggesting different interfaces with different outcomes on different promoters. Additionally, we documented a novel interaction of CSRP1 with TBX5, a member of the T-box family, implicated in the Holt-Oram syndrome. This interaction is not novel between the two classes of protein, since it was previously shown that the LIM4 and pdlim7 proteins regulate cellular localization of TBX5 during pectoral and heart development (Camarata et al., 2006, 2010a,b). In our case, the interaction is functional between the two proteins resulting in a synergistical activation of downstream target genes like NPPA. Although our results show that CSRP1 can coordinate protein partners through its LIM domain to form a robust network of transcriptional activators, it is still possible to speculate over the contribution of the LIM DNA binding-domain on its own on specific promoters (Kadrmas and Beckerle, 2004).

Despite the potential link that this interaction could suggest over the polydactyly phenotype observed in members of the family, the mutation in CSRP1 does not segregate with the limb phenotype, and thus we exclude any role for this mutation in the limb defects. This dichotomy in the genetic inheritance of two different phenotypes is not however novel in the case of CHD and limb defects. In fact, besides syndromic cases caused by monogenic mutations like *TBX1,3* and *5, SALL4*, and *EVC*, there are no published studies on variants that cause only cardiac and limb defects whether monogenic or multigenic (Kohlhase et al., 2002; Packham and Brook, 2003; Hills et al., 2011).

### CSRP1/TRPS1: a new digenic paradigm in CHD

Since CHD is a multifactorial disease in that it frequently reveals variable penetrance, genetic heterogeneity, and variable expressivity, it was essential to unravel other partners for CSRP1 that could account for the partial penetrance problem we faced in this particular family. Since the *CSRP1* variant is inherited from the father's side (II-8) who yet has a normal cardiac phenotype but has a limb defect, it was instrumental to go for whole-exome sequencing since cardiac targeted sequencing was not enough to explain the cardiac phenotypes in probands and we could not find a variation that explains the limb phenotype. The extracted data failed to show any monoallelic variation in genes previously shown to be linked to polydactyly (Biesecker, 2011) nor to yield a common variant between the 7 members with the same phenotype included in the screening. In contrast, the WES data yielded multiple variants inherited from the mother (II-9) and only present in the cardiac-affected probands. The *TRPS1* variant (p.R311S) stands alone among a short list of missense variants (Supplementary Table 4), since it was novel, absent from the gnomad database, as well from 200 Lebanese exomes. In addition both the *TRPS*1 and *CSRP1* variants were absent from regional databases like the Saudi Genome project, reinforcing again the claim that both are responsible for the cardiac phenotype. Genetically, besides the *TRPS1* variant, only a SNV in *SHOX2* (Supplementary Table 4) could have had a deleterious effect on cardiac development, but the fact that SHOX2 is exclusively expressed in the sinoatrial node makes it less plausible in this particular familial case (Puskaric et al., 2010; Hoffmann et al., 2016). The *in silico* analysis shows that this variant would have a moderate effect on the function of the protein, and we thus hypothesized that alone it would not affect cardiac development, thus explaining the normal phenotype of the mother. Since probands III-8, -10, and -12 who carry the *CSRP1* and *TRPS1* variants expressed a cardiac phenotype, we hypothesized that the *TRPS1* variant has no effect unless it is expressed with the *CSRP1* variant. Mutations or deletions in *TRPS1* give rise to Tricho-rhino-phalangeal syndrome (TRPS), and a relatively high proportion of patients with TRPS exhibit CHD, ranging from minor to severe anomalies (Verheij et al., 2009; Maas et al., 2015). Although previous reports did not describe Trps1 expression in the mammalian heart, Trps1 was recently shown to be expressed in a restricted region within the cardiac cushion of OFT and developing valves (Nomir et al., 2016). This could partially explain why patients with *TRPS1* mutations show a broad range of congenital cardiac defects. In our case, we establish a direct physical interaction between the two proteins that was affected by the *CSRP1* variant. However, we suspect a much more complicated functional regulation since TRPS1 acts as a repressor and CSRP1 as a weak activator of downstream target genes. We hypothesize that the transcriptomal assembly of CSRP1 and TRPS1 co-factors over cardiac promoters, would be largely affected by the mutations, and cause the observed phenotypes in the family members along the broad spectrum of phenotypes associated to CHD. We also suggest that the inhibitory role of TRPS1 over the GATA-driven promoters is at stake in this interaction, and that the mutation in *CSRP1* would specifically affect GATA4 transcriptional activation in a context where both TRPS1 and GATA4/5/6 are competing for DNA occupancy.

Although efficient strategies such as whole exome sequencing potentially contribute to the understanding of rare human diseases and allows the detection of multiple rare variants, they are still short of elucidating the network of such genes that are involved in CHD (Postma et al., 2016). Thus, genome-wide association studies (GWAS) should be combined with WES or even WGS since they have provided evidence that common genetic variation can influence the risk of certain types of CHD (detect somatic mutations and noncoding sequences). Many whole-genome CNV screening studies indeed have revealed that a significant number of CHD patients have pathogenic CNVs. The highest frequency of pathogenic CNVs are found in patients who have both CHD and extra-cardiac anomalies which is similar to our case (Andersen et al., 2014).

## Conclusion

Dissecting phenotypes and establishing direct genotype/phenotype interaction is a must in any strategical approach in CHD. We have shown that despite the high consanguinity within one family, there are no homozygous mutations that could explain the associated cardiac and limb defects. In contrast, a digenic model of inheritance is proposed to explain the cardiac phenotype.

## Author contributions

AK, AF, KS: did all the experiments, and participate in the analysis and writing up of the manuscript. SB-S and NE-H: did the bioinformatics analysis. MA, MK, RH, and FB: did the clinical assessment and recruitment of the patients and their family members. JS, CS, EB, FB, and GN designed the study, secured the funding, analyzed the data, and wrote up the manuscript.

### Conflict of interest statement

The authors declare that the research was conducted in the absence of any commercial or financial relationships that could be construed as a potential conflict of interest.
