# Supplementary material for: A Novel Role for CSRP1 in a Lebanese Family with Congenital Cardiac Defects
Source: Front Genet. 2017 Dec 18;8:217. doi: 10.3389/fgene.2017.00217 (PMC5741687; doi:10.3389/fgene.2017.00217)
Supplement: Supplementary file 1 [file Table1.PDF]

Supplementary Table 1: List of Targeted Sequencing Genes

ACTA2	FBN1	LEFTY2	PEX13	TFAP2B
ACTC1	FBN2	LRP2	PITX2	TLL1
ACVR1	FGFR1	MAX	PKD2	TWIST1
AHSA2	FLNA	MEF2A	PLAGL1	UBR1<br>USP34
ANKRD1	FOXC1	MEF2C	PPM1K	VEGFA
ASXL2	FOXH1	MGP	PPP3CA	VEGFC
BCL11A	FOXL2	MID1	TBX20	USP34
BCOR	GATA4	MSX1	TBX3	ZEB2
BMP4	GATA5	MSX2	PTPN11	ZFPM1
BMP7	GATA6	MYH11	RAB10	ZFPM2
BMPR2	GDF1	MYH6	RAB23	ZIC3
CCT4	GJA1	NF1	RAI1	
CFC1	GJA9	NFATC3	RAI2	
CHD7	GPC3	NFATC4	REL	
CITED2	HAND1	NIPBL	ROR2	
CREBBP	HAND2	NKX2-5	SALL1	
CRELD1	HES1	NKX2-6	SALL4	
CSRP1	HES4	NODAL	SEMA3E	
CTNNA3	HEY2	NOTCH1	SESN1	
DHCR7	HOXA1	NOTCH2	SHOC2	
DNAI1	ID2	NPHP3	SLC2A10	
DVL1	IGFBP4	NSD1	SOS1	
ELN	ISL1	OSR1	SRF	
EP300	JAG1	PAPOLG	TBX1	
ESCO2	KCNJ2	PCMTD2	TBX5	
EVC	KLF13	PCSK5	TCF21	
EVC2	LBR	PEX1	TDGF1	
