# Supplementary material for: A Novel Role for CSRP1 in a Lebanese Family with Congenital Cardiac Defects
Source: Front Genet. 2017 Dec 18;8:217. doi: 10.3389/fgene.2017.00217 (PMC5741687; doi:10.3389/fgene.2017.00217)
Supplement: Supplementary file 2 [file Table2.PDF]

Supplementary Table 2: Variants inherited from either parents with a MAF< 5%

Gene	Variant	Chr	Coordinate	Genotype	Read Depth	Consequence	Protein Positio	Amino Acids	Sift	PolyPhen	dbSNP ID	Global Allele Freq
LOC1009964	CCCG>GCCGCC/GCCGCC	X	153151277	hom	6	frameshift_variant						0
SCARF2	G>CC/CC	22	20780031	hom	15	frameshift_variant	749				rs5844423	0
MMP12	G>TT/TT	11	102738795	hom	109	frameshift_variant	210				rs5003690	0
ZNF717	GTCC>TTTCT/TTTCT	3	75790811	hom	4	frameshift_variant						0
CDCP2	T>TG/TG	1	54605318	hom	76	frameshift_variant, feature_elongation					rs3841798, rs7	0
HSH2D	TA>T/T	19	16268207	hom	110	frameshift_variant, feature_truncation	221				rs5827321	0
LOC283710	CG>C/C	15	31521505	hom	103	frameshift_variant, feature_truncation	26				rs71110857, rs	0
OR2T35	TCAGCACG>T/T	1	248801944	hom	15	frameshift_variant, feature_truncation					rs143010547	0
HRNR	AT>A/A	1	152195728	hom	52	frameshift_variant, feature_truncation	1				rs34061715	0
SAMD1	C>CG/CG	19	14200896	hom	16	frameshift_variant, splice_region_variant, feature_elongation						0
FLJ22184	A>AG/AG	19	7938306	hom	71	frameshift_variant, splice_region_variant, feature_elongation	90				rs58971992, rs7	0
ZNF778	G>GGTGA/GGTGA	16	89291210	hom	39	frameshift_variant, splice_region_variant, feature_elongation	110				rs113560259	0
MROH8,RPN	G>GCTTATAGACAGGGCCCCGCGGCCGGCACT/GCTT	20	35807790	hom	11	intron_variant, feature_elongation	0				rs147277915, rs	0
STRN4,FKRP	A>AGGCCG/AGGCCG	19	47249351	hom	21	intron_variant, feature_elongation	0					0
C21orf49,C2	A>T/T	21	34166190	hom	119	missense_variant	181	F/L	tolerated(1)	benign(0)	rs74617086	0
RFPL4AL1	G>A/A	19	56284457	hom	160	missense_variant	259	G/E	tolerated(1)	benign(0.004)	rs78244980	0
RFPL4AL1	A>T/T	19	56284396	hom	53	missense_variant	239	N/Y	tolerated(1)	benign(0)	rs141642534	0
KRTAP4-9	C>T/T	17	39261933	hom	40	missense_variant	98	A/V	tolerated(1)	unknown(0)	rs7207685	0
AGAP7	A>T/T	10	51465552	hom	4	missense_variant	302	Y/N	tolerated(1)	benign(0)	rs76363375	0
GPRIN2	A>G/G	10	46999604	hom	238	missense_variant	242	R/G	tolerated(1)	benign(0)	rs3127683	0
FAM205A	T>C/C	9	34725742	hom	184	missense_variant	499	M/V	tolerated(1)	benign(0)	rs62547039	0
FAM115C	A>G/G	7	143400301	hom	6	missense_variant	72	T/A	tolerated(1)	benign(0.001)	rs80035595	0
POTEI	T>C/C	2	131221102	hom	3	missense_variant	839	M/V	tolerated(1)	benign(0)	rs139258542	0
OR2T12	C>G/G	1	248458717	hom	57	missense_variant	55	R/T	tolerated(1)	benign(0)	rs12745228	0
FCGR3B	T>C/C	1	161599693	hom	143	missense_variant	101	N/S	tolerated(1)	benign(0)	rs76714703	0
CLCC1	G>A/A	1	109482304	hom	148	missense_variant	332	A/V	tolerated(1)	benign(0.001)	rs36032236	1
FCGBP	A>G/G	19	40374034	hom	54	missense_variant	4015	V/A	tolerated(0.99)	benign(0.001)	rs138587194	0
MUC20	G>A/A	3	195451881	hom	387	missense_variant	136	R/K	tolerated(0.94)	benign(0)	rs146888306	0
MRVI1	T>C/C	11	10647977	hom	75	missense_variant	302	T/A	tolerated(0.84)	benign(0.002)	rs74898367	1
PRDM9	C>G/G	5	23527239	hom	18	missense_variant	681	T/S	tolerated(0.81)	benign(0.003)	rs6875787	0
NCF1	G>A/A	7	74193668	hom	49	missense_variant	99	G/S	tolerated(0.74)	benign(0.004)	rs17856077, rs1	0
KIR3DL3	G>A/A	19	55239176	hom	70	missense_variant	152	R/H	tolerated(0.69)	benign(0.035)	rs199609975	0
ACOT2	A>G/G	14	74041748	hom	10	missense_variant	328	H/R	tolerated(0.63)	benign(0.006)	rs149033118	0
OR2T11	G>T/T	1	248789967	hom	68	missense_variant	155	L/M	tolerated(0.62)	possibly_damaging(0.753)		0
UBL4B	A>G/G	1	110655407	hom	143	missense_variant	84	Q/R	tolerated(0.62)	benign(0.113)		0
KRTAP4-8	C>T/T	17	39253835	hom	21	missense_variant	168	A/T	tolerated(0.61)	unknown(0)	rs72625995	0
ZNF717	C>T/T	3	75787728	hom	7	missense_variant	349	R/H	tolerated(0.61)	benign(0)	rs75074846, rs1	0
RIIAD1	A>G/G	1	151701278	hom	90	missense_variant	82	H/R	tolerated(0.58)	benign(0.001)	rs146920112	0.09
MUC20	A>G/G	3	195451880	hom	379	missense_variant	136	R/G	tolerated(0.55)	benign(0)	rs142096782	0
TOMM40	T>G/G	19	45394819	hom	3	missense_variant	49	S/R	tolerated(0.54)	unknown(0)	rs11556510	1
CDK5RAP3	G>A/A	17	46052883	hom	43	missense_variant	173	E/K	tolerated(0.52)	benign(0.04)	rs61741125	2
DPP9	G>A/A	19	4714275	hom	48	missense_variant	44	A/V	tolerated(0.47)	benign(0)	rs147966989	1
OR2T5	A>G/G	1	248651927	hom	45	missense_variant	13	K/R	tolerated(0.47)	benign(0)	rs201060805	0
C19orf45	T>A/A	19	7565988	hom	25	missense_variant	94	W/R	tolerated(0.43)	benign(0)	rs79777490	2
KIR2DL3	G>T/T	19	55263185	hom	3	missense_variant	267	R/L	tolerated(0.42)	benign(0.007)	rs140667350	0
TBC1D26	A>G/G	17	15640815	hom	10	missense_variant	59	H/R	tolerated(0.42)	benign(0)	rs201215351	0
PRSS3	A>G/G	9	33798017	hom	200	missense_variant	188	T/A	tolerated(0.41)	benign(0.007)	rs855581	0
KLF14	A>C/C	7	130418720	hom	9	missense_variant	47	H/Q	tolerated(0.39)	benign(0)	rs111359117	0
NBPF14,TXNI	G>A/A	1	145440096	hom	170	missense_variant	177	R/Q	tolerated(0.39)	benign(0.004)	rs6674773	1
RFPL4AL1	T>G/G	19	56284507	hom	188	missense_variant	276	S/A	tolerated(0.37)	benign(0.001)	rs115250131	0
MSTO1	A>G/G	1	155583937	hom	5	missense_variant	529	K/R	tolerated(0.37)	benign(0.058)	rs147713038	0
PCDHA1,PCD	G>A/A	5	140214381	hom	93	missense_variant	138	R/K	tolerated(0.36)	benign(0)	rs10067182	0
CHAF1A	G>A/A	19	4433253	hom	143	missense_variant	797	R/Q	tolerated(0.35)	benign(0.065)	rs45597332	1
KCNC3	T>C/C	19	50832152	hom	17	missense_variant	63	D/G	tolerated(0.34)	unknown(0)		0
KRTAP9-6	A>G/G	17	39421781	hom	13	missense_variant	51	Y/C	tolerated(0.32)	unknown(0)	rs12938374	0
OR2T35	A>T/T	1	248801778	hom	8	missense_variant	261	L/Q	tolerated(0.31)	probably_damaging(0.998)	rs201643849	0
RNF19B	T>G/G	1	33430102	hom	7	missense_variant	62	Q/P	tolerated(0.31)	unknown(0)	rs113840389	0
PVRL2	G>A/A	19	45385488	hom	9	missense_variant	355	A/T	tolerated(0.27)	benign(0.025)	rs187706273	0.18
RFPL4AL1	A>G/G	19	56284535	hom	124	missense_variant	285	E/G	tolerated(0.22)	benign(0)	rs144990325	0
OR2T29	T>C/C	1	248722722	hom	22	missense_variant	24	Q/R	tolerated(0.22)	benign(0.004)	rs199528787	0
POTEF	G>A/A	2	130832185	hom	7	missense_variant	954	R/W	tolerated(0.21)	benign(0.067)	rs202181461	0
PCSK4	G>A/A	19	1482396	hom	30	missense_variant	592	T/I	tolerated(0.2)	benign(0.217)	rs61733913	0.18
OR2T12	T>C/C	1	248458676	hom	12	missense_variant	69	M/V	tolerated(0.2)	benign(0.001)	rs6678138	0
POTEI	C>T/T	2	131221170	hom	20	missense_variant	816	R/H	tolerated(0.17)	probably_damaging(0.993)	rs144934754	0
POTEF	T>A/A	2	130832292	hom	15	missense_variant	918	Y/F	tolerated(0.16)	possibly_damaging(0.844)	rs75775141	0
ZNF812	C>T/T	19	9801832	hom	269	missense_variant	116	G/D	tolerated(0.15)	benign(0.001)	rs62105698	1
RFPL4AL1	C>A/A	19	56284529	hom	130	missense_variant	283	S/Y	tolerated(0.14)	benign(0.001)	rs199519773	0
KIR2DL3	G>A/A	19	55263191	hom	2	missense_variant	269	C/Y	tolerated(0.14)	benign(0.025)	rs138488498	0
SIRPB1	C>A/A	20	1559233	hom	95	missense_variant	62	V/L	tolerated(0.13)	benign(0.044)	rs41275424	0.46
TCP10L2	T>C/C	6	167592524	hom	22	missense_variant	228	L/P	tolerated(0.13)	benign(0)	rs2989545	0
DAP3	G>A/A	1	155706776	hom	44	missense_variant	345	V/I	tolerated(0.11)	possibly_damaging(0.5)	rs41264967	0.37
FBN3	G>A/A	19	8212229	hom	67	missense_variant	46	R/W	deleterious(0.03)	probably_damaging(0.988)	rs142752628	0.46
ANKRD24	C>T/T	19	4200157	hom	36	missense_variant	111	A/V	deleterious(0.03)	benign(0.002)		0
C2CD4A	C>G/G	15	62360366	hom	4	missense_variant	185	P/R	deleterious(0.03)	probably_damaging(0.99)		0
LRP1B	G>A/A	2	141598583	hom	238	missense_variant	1673	T/M	deleterious(0.03)	possibly_damaging(0.829)	rs199519370	0
PRKACA	C>T/T	19	14211656	hom	94	missense_variant	134	R/Q	deleterious(0.02)	benign(0.309)		0
PPP2R2C	G>A/A	4	6383507	hom	59	missense_variant	23	P/S	deleterious(0.02)	benign(0.002)	rs116399332	0.09
OR10J3	G>A/A	1	159284100	hom	133	missense_variant	117	T/I	deleterious(0.02)	benign(0.036)		0
PPIAL4E	A>G/G	1	148644408	hom	6	missense_variant	108	N/S	deleterious(0.02)	benign(0.131)	rs200516190	0
DHX35	T>C/C	20	37621052	hom	157	missense_variant	189	I/T	deleterious(0.01)	benign(0.004)	rs36053162	2
KPNA2	T>A/A	17	66042639	hom	34	missense_variant	506	V/D	deleterious(0.01)	possibly_damaging(0.847)	rs138669765	2
LGALS9B	A>G/G	17	20353347	hom	4	missense_variant	336	L/P	deleterious(0)	probably_damaging(0.999)		0
ANKRD60	A>T/T	20	56803871	hom	2	downstream_gene_variant	0					0
ZNF717	T>C/C	3	75787620	hom	4	missense_variant	385	H/R	deleterious(0)	probably_damaging(0.977)	rs145606249, rs	0
FARP2	G>A/A	2	242312572	hom	33	missense_variant	17	R/H	deleterious(0)	probably_damaging(0.987)	rs61739702	2
FARSA	G>A/A	19	13035732	hom	74	downstream_gene_variant	0				rs201276620	0.09
LOC1009964	C>G/G	X	153149715	hom	36	missense_variant	14	P/A		benign(0)	rs6643650	0
CD177	T>G/G	19	43860192	hom	4	missense_variant	184	V/G			rs71337594	0
MUC16	C>G/G	19	8987087	hom	65	missense_variant	13968	S/T		probably_damaging(0.943)		0
POLDIP2	T>G/G	17	26684394	hom	15	missense_variant	27	Q/P			rs113730440	0
ZFPM1	AGCCTCTGG>CCC/CCC	16	88599697	hom	9	missense_variant		EPLA/AP				0
ZNF598	T>G/G	16	2059708	hom	16	missense_variant	14	H/P			rs71384662	0
SLC35G3	C>T/T	17	33520642	hom	2	downstream_gene_variant	0				rs149382529	0
OVOS	G>T/T	12	9707329	hom	7	missense_variant	776	R/L			rs201854834	0
OVOS	T>C/C	12	9707317	hom	5	missense_variant	772	V/A			rs200514101	0
MUC12	T>C/C	7	100646199	hom	12	missense_variant	4119	F/L		unknown(0)	rs181671450	0
MUC12	A>C/C	7	100639095	hom	5	missense_variant	1751	T/P		unknown(0)	rs71557212	0
MUC12	C>T/T	7	100639066	hom	16	missense_variant	1741	S/L		unknown(0)	rs28483629	0
MUC12	G>C/C	7	100639049	hom	29	missense_variant	1735	L/F		unknown(0)	rs56357244	0
MUC12	C>T/T	7	100639012	hom	67	missense_variant	1723	T/I		unknown(0)	rs55638317	0
MUC12	A>G/G	7	100638856	hom	64	missense_variant	1671	H/R		unknown(0)	rs76496013	0
MUC12	C>A/A	7	100637193	hom	13	missense_variant	1117	P/T		unknown(0)	rs77621644	0
MUC12	G>A/A	7	100636408	hom	15	missense_variant	855	R/H		unknown(0)	rs142190605	0
MUC3A	A>C/C	7	100550766	hom	40	missense_variant	449	R/S			rs28654698	0
MUC3A	T>A/A	7	100550737	hom	40	missense_variant	440	F/I			rs66606045	0
LOC1002872	T>G/G	7	57008674	hom	12	missense_variant	124	N/T			rs201042309	0
HLA-DRB5	AGCG>CTCA/CTCA	6	32489791	hom	19	missense_variant		DA/DE				0
TREH	C>CG/CG	11	118529044	hom	8	downstream_gene_variant	0				rs11448549	0
MUC4	T>C/C	3	195512186	hom	22	missense_variant	2089	I/V		benign(0.004)	rs202060675	0
MUC4	T>A/A	3	195512107	hom	46	missense_variant	2115	D/V		benign(0.004)		0
MUC4	T>C/C	3	195512042	hom	46	missense_variant	2137	T/A		benign(0.322)		0
MUC4	A>G/G	3	195511331	hom	50	missense_variant	2374	S/P		benign(0.353)	rs56359992	0
HBG1	G>A/A	11	5270686	hom	18	downstream_gene_variant	0				rs1061234	0
MUC4	A>G/G	3	195506473	hom	5	missense_variant	3993	V/A		benign(0.148)	rs201922637	0
THSD7B	G>A/A	2	138208478	hom	189	missense_variant	977	S/N				0
C9orf66,DOC	G>T/T	9	215060	hom	61	intron_variant	0				rs141134256	2
NBPF1	T>G/G	1	16895634	hom	0	missense_variant	850	K/Q			rs75027498	0
HNRNPCP5	T>C/C	1	13183439	hom	151	missense_variant	145	H/R			rs28713604	0
FAM153A	A>G/G	5	177163580	hom	4	missense_variant, splice_region_variant	145	F/L	tolerated(1)	benign(0)	rs201479484	0
CATSPERD	A>C/C	19	5766166	hom	58	missense_variant, splice_region_variant	520	N/T	deleterious(0.01)	probably_damaging(0.984)		0
UNKL	TCCCCGTGCTTCACACTGGGGCAGGGCGTGGACCTG	16	1447264	hom	11	splice_acceptor_variant, coding_sequence_variant, intron_variant, feature_truncation						0
ANO7	G>T/T	2	242157307	hom	29	splice_donor_variant	0					0
RBBP6	GTA>G/G	16	24564879	hom	11	splice_donor_variant, intron_variant, feature_truncation	0				rs72133882	0
LDLR	G>A/A	19	11231203	hom	39	splice_region_variant, intron_variant	0				rs72658867	0.23
2-Mar	C>T/T	19	8503267	hom	8	splice_region_variant, intron_variant	0				rs79496212	1
CHD3	C>G/G	17	7788409	hom	6	splice_region_variant, intron_variant	0				rs201772788	0
APIP	AA>C/C	11	34909832	hom	10	splice_region_variant, intron_variant	0					0
ADAP1	G>GGGCAAAGGCA/GGGCAAAGGCA	7	940258	hom	14	downstream_gene_variant	0				rs113839920	0
GOT1	G>A/A	10	101163636	hom	16	splice_region_variant, intron_variant	0				rs139135226	0.23
GPAA1	C>T/T	8	145140777	hom	162	splice_region_variant, intron_variant	0				rs117116707	0.41
FAM86B1	T>C/C	8	12042880	hom	39	splice_region_variant, intron_variant	0				rs142379100	0
THOC3	C>T/T	5	175387139	hom	9	splice_region_variant, intron_variant	0				rs79720333	0
DDX60L	A>G/G	4	169312801	hom	70	splice_region_variant, intron_variant	0				rs56794833	0
FAM184B	GC>ACAA/ACAA	4	17634255	hom	29	splice_region_variant, intron_variant	0					0
NMD3	T>A/A	3	160939776	hom	13	splice_region_variant, intron_variant	0				rs28711573	0

DNAH12	T>A/A	3	57431752	hom	28	splice_region_variant, intron_variant	0				rs148564870	0
LIMD1	GCA>TGC/TGC	3	45677637	hom	189	splice_region_variant, intron_variant	0					0
DOPEY2	A>AT/AT	21	37619931	hom	57	splice_region_variant, intron_variant, feature_elongation	0				rs5843755, rs11	0
SOS2	C>CA/CA	14	50585574	hom	13	splice_region_variant, intron_variant, feature_elongation	0				rs75654803, rs1	0
CCNT1	G>GA/GA	12	49091972	hom	11	splice_region_variant, intron_variant, feature_elongation	0				rs11420004	0
WNK1	C>CT/CT	12	987533	hom	41	splice_region_variant, intron_variant, feature_elongation	0				rs112284021, rs	0
NXPE1	C>CA/CA	11	114398627	hom	49	splice_region_variant, intron_variant, feature_elongation	0				rs34542949, rs5	0
TRIM64C	C>CAA/CAA	11	49076963	hom	38	splice_region_variant, intron_variant, feature_elongation	0				rs34650085, rs1	0
PTPRD	A>AAACTTACCATTCTTGAACTGT/AAACTTACCATTC	9	8331574	hom	39	splice_region_variant, intron_variant, feature_elongation	0				rs146237556	0
NDUFAF6	T>TA/TA	8	96047806	hom	15	splice_region_variant, intron_variant, feature_elongation	0				rs34960210	0
AP3B1	T>TA/TA	5	77524068	hom	44	splice_region_variant, intron_variant, feature_elongation	0				rs35569618, rs5	0
ANXA5	T>TA/TA	4	122590883	hom	24	splice_region_variant, intron_variant, feature_elongation	0				rs199756402, rs	0
SMC4	A>ATGTGTGTGTGTGTGTG/ATGTGTGTGTGTGTGTG	3	160142810	hom	34	splice_region_variant, intron_variant, feature_elongation	0					0
SGK2	GGTGT>G/G	20	42199704	hom	22	splice_region_variant, intron_variant, feature_truncation	0				rs11467250, rs6	0
DNMT1	GA>G/G	19	10270746	hom	61	splice_region_variant, intron_variant, feature_truncation	0				rs5827087, rs3	0
INSR	GGA>G/G	19	7184651	hom	10	splice_region_variant, intron_variant, feature_truncation	0				rs3835070	0
ESCO1	GAT>G/G	18	19119974	hom	27	splice_region_variant, intron_variant, feature_truncation	0				rs35031202	0
TUBD1	GA>G/G	17	57937792	hom	30	splice_region_variant, intron_variant, feature_truncation	0				rs34427733	0
DHX40	TA>T/T	17	57663596	hom	44	splice_region_variant, intron_variant, feature_truncation	0				rs58298344	0
HDAC5	TG>T/T	17	42170181	hom	21	splice_region_variant, intron_variant, feature_truncation	0				rs67111880	0
ZNF18	GA>G/G	17	11887518	hom	43	splice_region_variant, intron_variant, feature_truncation	0				rs34299203	0
DYNC1LI2	GA>G/G	16	66757728	hom	23	splice_region_variant, intron_variant, feature_truncation	0				rs77481802	0
GALC	GA>G/G	14	88417095	hom	39	splice_region_variant, intron_variant, feature_truncation	0				rs11300320	0
ZFC3H1	TA>T/T	12	72028607	hom	21	splice_region_variant, intron_variant, feature_truncation	0				rs34399921	0
PLEKHA5	AT>A/A	12	19410474	hom	23	splice_region_variant, intron_variant, feature_truncation	0				rs10706958	0
RTKN2	GAAAAA>G/G	10	63977011	hom	40	splice_region_variant, intron_variant, feature_truncation	0				rs148142088	0
GSAP	CA>C/C	7	76984752	hom	45	splice_region_variant, intron_variant, feature_truncation	0				rs68053062, rs3	0
IL20RA	TAA>T/T	6	137325901	hom	10	splice_region_variant, intron_variant, feature_truncation	0					0
ASCC3	TA>T/T	6	101215223	hom	40	splice_region_variant, intron_variant, feature_truncation	0				rs11345864	0
RNF145	GAAAAA>G/G	5	158588633	hom	24	splice_region_variant, intron_variant, feature_truncation	0				rs139682876	0
BRD8	TAA>T/T	5	137507104	hom	28	splice_region_variant, intron_variant, feature_truncation	0				rs200476181	0
PET112	TA>T/T	4	152609920	hom	23	splice_region_variant, intron_variant, feature_truncation	0				rs11292952	0
MYH15	CA>C/C	3	108179219	hom	13	splice_region_variant, intron_variant, feature_truncation	0				rs11303627	0
DNAH7	GA>G/G	2	196756535	hom	36	splice_region_variant, intron_variant, feature_truncation	0				rs11292337, rs3	0
SPC25	GAAA>G/G	2	169728069	hom	24	splice_region_variant, intron_variant, feature_truncation	0				rs201633076, rs	0
ITGB6	GAA>G/G	2	160958349	hom	12	splice_region_variant, intron_variant, feature_truncation	0				rs5835793, rs2	0
DARS	TA>T/T	2	136690414	hom	35	splice_region_variant, intron_variant, feature_truncation	0				rs34744196	0
PCDP1	AT>A/A	2	120404629	hom	67	splice_region_variant, intron_variant, feature_truncation	0				rs11349518	0
WDR64	TA>T/T	1	241954005	hom	28	splice_region_variant, intron_variant, feature_truncation	0				rs35435449	0
TTC13	GA>G/G	1	231094050	hom	45	splice_region_variant, intron_variant, feature_truncation	0				rs60736833	0
NBPF14,PDE	TAGGAAGAAACGGCA>T/T	1	144923823	hom	145	splice_region_variant, intron_variant, feature_truncation	0				rs11268079	0
PTPN22	TA>T/T	1	114372332	hom	10	splice_region_variant, intron_variant, feature_truncation	0				rs57877024	0
AKNAD1	TA>T/T	1	109377157	hom	29	splice_region_variant, intron_variant, feature_truncation	0				rs5776950, rs34	0
COL11A1	GA>G/G	1	103496805	hom	20	splice_region_variant, intron_variant, feature_truncation	0				rs67059272, rs3	0
CCDC144B	G>GA/GA	17	18498497	hom	16	splice_region_variant, intron_variant, nc_transcript_variant, featu	0				rs59933375	0
C21orf49	GT>G/G	21	34157235	hom	22	splice_region_variant, intron_variant, nc_transcript_variant, featu	0				rs78077441, rs1	0
PEX19	G>A/A	1	160253385	hom	100	downstream_gene_variant	0				rs144256391	0.05
DCST1	C>T/T	1	155014280	hom	141	downstream_gene_variant	0					0
SLAIN1	G>GC/GC	13	78272276	hom	6	upstream_gene_variant	0				rs71102772, rs2	0
EMG1	T>GC/GC	12	7080212	hom	65	upstream_gene_variant	0				rs11428482	0
GPSM1	T>C/C	9	139222174	hom	4	upstream_gene_variant	0				rs28495820	0
RAB6C	G>A/A	2	130738163	hom	235	upstream_gene_variant	0				rs144773989	0
APOA2	CCACACA>C/C	1	161192845	hom	50	upstream_gene_variant	0				rs141599125, rs	0
IGSF9	TAA>T/T	1	159897681	hom	44	upstream_gene_variant	0				rs142211405	0
OAZ3	A>T/T	1	151739761	hom	53	upstream_gene_variant	0					0
OAZ3	C>T/T	1	151739736	hom	54	upstream_gene_variant	0					0
ZNF233	GTTG>GTTG/ATA	19	44778796	het	35	frameshift_variant						0
PKD1L2	TTT>TTT/C	16	81242149	het	115	frameshift_variant						0
IL32	A>A/GG	16	3119304	het	82	frameshift_variant	172					0
ZAN	GTT>GTT/ATTG	7	100371474	het	106	frameshift_variant						0
DDIT4L	TT>TT/C	4	101108876	het	47	frameshift_variant	180					0
CLDN16	GG>GG/C	3	190106073	het	160	frameshift_variant					rs3214506	0
TCEAL8	G>G/GT	X	102508874	het	109	frameshift_variant, feature_elongation						0
ABHD3	A>A/ACACG	18	19231604	het	142	frameshift_variant, feature_elongation	393					0
SYCE1L	C>C/CG	16	77246091	het	37	frameshift_variant, feature_elongation	163					0
ATXN3	C>C/CTGCTGCTGCTGCTGCTG	14	92537354	het	50	frameshift_variant, feature_elongation						0
FAM186A	C>C/CCTGCTGAGGGGTGAGAGGGATCCCCAGGGCC	12	50745863	het	77	frameshift_variant, feature_elongation	1584					0
IFNA17	A>A/AT	9	21228002	het	79	frameshift_variant, feature_elongation	57				rs200400518, rs	2
KMT2C	G>G/GT	7	151945071	het	467	frameshift_variant, feature_elongation	816					0
FAM20C	G>G/GGACAGGTGAGCCCTTCCTTCCTCCCTCCATCCC	7	286468	het	59	frameshift_variant, feature_elongation						0
NOP16	C>C/CGT	5	175811094	het	134	frameshift_variant, feature_elongation	196				rs56989856, rs7	0
NOP16	C>C/CAT	5	175811094	het	134	frameshift_variant, feature_elongation	196				rs56989856, rs7	0
C4orf51	G>G/GA	4	146601514	het	149	frameshift_variant, feature_elongation						0
PRSS12	C>C/CATCA	4	119229713	het	199	frameshift_variant, feature_elongation	503					0
CRIPAK	G>G/GTGCCCATGTGGAGTGCCCGCCTGCTCACACA	4	1388350	het	141	frameshift_variant, feature_elongation						0
ZNF806	C>C/CA	2	133075904	het	172	frameshift_variant, feature_elongation					rs144380111	0
ANKRD36C	C>C/CA	2	96610395	het	190	frameshift_variant, feature_elongation					rs147285461, rs	0
CSRP1	T>T/TCAAGGCCTTTGCCA	1	201454455	het	80	frameshift_variant, feature_elongation	154					0
PSG6	GA>GA/G	19	43420567	het	103	frameshift_variant, feature_truncation	46					0
NME4	CT>CT/C	16	447234	het	8	frameshift_variant, feature_truncation	5					0
LOC732265	TA>TA/T	15	74392545	het	13	frameshift_variant, feature_truncation	2				rs3214584	0
ZNF660	CAT>CAT/C	3	44636278	het	34	frameshift_variant, feature_truncation						0
ZNF806	CA>CA/C	2	133076117	het	272	frameshift_variant, feature_truncation	580				rs111405036	0
ZNF806	TC>TC/T	2	133075478	het	142	frameshift_variant, feature_truncation	367				rs111944984	0
NOTCH2	CGG>CGG/C	1	120612002	het	33	frameshift_variant, feature_truncation	6					0
HNRNPCL1	CT>CT/C	1	12907357	het	61	frameshift_variant, feature_truncation	262					0
CELA1	TAAAGGAC>TAAAGGAC/GGAAGCG	12	51740409	het	11	frameshift_variant, splice_region_variant						0
NFATC1	T>T/TCCCAGCGCTCAGCTCAGGAGCTGCCTCTG	18	77211126	het	51	frameshift_variant, splice_region_variant, feature_elongation	575					0
PDE4D	ATCT>ATCT/A	5	58476466	het	226	inframe_deletion, splice_region_variant	254	R/-				0
PCDHA1,PCD	A>A/ACCAACTGATCTGATATATTGTATAGTTTAATAG	5	140222137	het	84	intron_variant, feature_elongation	0					0
UBE2K,ZBTB1	T>T/TGCACGTTCCTGTGGTGGTTGAGGTTGTTGCTG	14	39771939	het	86	intron_variant, feature_elongation	0					0
RAB34,NARR	TG>TG/T	17	27044589	het	89	intron_variant, feature_truncation	0					0
RNF213	A>A/G	17	78357478	het	76	intron_variant, nc_transcript_variant	0				rs12944088	2
TAS2R43	C>C/G	12	11244631	het	50	intron_variant, nc_transcript_variant	0				rs138563991	1
TAS2R43	G>G/C	12	11244369	het	160	intron_variant, nc_transcript_variant	0				rs200586631	0
TAS2R43	A>A/C	12	11244319	het	224	intron_variant, nc_transcript_variant	0				rs116243872	2
TAS2R43	C>C/A	12	11244230	het	193	intron_variant, nc_transcript_variant	0				rs144622176	2
TAS2R46	A>A/G	12	11214472	het	139	intron_variant, nc_transcript_variant	0				rs200936852	0
TAS2R46	T>T/C	12	11214455	het	165	intron_variant, nc_transcript_variant	0				rs72477411	0
TAS2R31	A>A/C	12	11183722	het	90	intron_variant, nc_transcript_variant	0				rs78562467	0
TAS2R31	C>C/A	12	11183697	het	124	intron_variant, nc_transcript_variant	0				rs73049074	0
TAS2R31	C>C/T	12	11183676	het	141	intron_variant, nc_transcript_variant	0				rs73049072	0
TAS2R31	A>A/G	12	11183642	het	147	intron_variant, nc_transcript_variant	0				rs73049067	0
TAS2R31	T>T/C	12	11183576	het	97	intron_variant, nc_transcript_variant	0				rs72475488	0
TAS2R31	T>T/A	12	11183224	het	242	intron_variant, nc_transcript_variant	0				rs116926686	1
TAS2R31	C>C/A	12	11183092	het	388	intron_variant, nc_transcript_variant	0				rs139069360	1
TAS2R19	T>T/A	12	11174715	het	120	intron_variant, nc_transcript_variant	0				rs75356565	0
TAS2R19	T>T/C	12	11174498	het	80	intron_variant, nc_transcript_variant	0				rs115299813	1
TAS2R19	G>G/A	12	11174390	het	183	intron_variant, nc_transcript_variant	0				rs74992161	0
TAS2R19	C>C/T	12	11174380	het	196	intron_variant, nc_transcript_variant	0				rs76970958	0
TAS2R19	C>C/G	12	11174372	het	205	intron_variant, nc_transcript_variant	0				rs74772077	0
KRTAP5-AS1,	G>G/C	11	1605983	het	13	intron_variant, nc_transcript_variant	0				rs59007122	0
TTN-AS1,TTN	A>A/C	2	179401074	het	179	intron_variant, nc_transcript_variant	0				rs200166942	0
ZNF695	C>C/T	1	247150739	het	64	intron_variant, nc_transcript_variant	0				rs61753584	0
L1CAM	C>C/T	X	153135080	het	162	missense_variant	388	A/T	tolerated(1)	benign(0.013)		0
ZXDB	A>A/C	X	57618849	het	11	missense_variant	123	E/A	tolerated(1)	benign(0)	rs200267267	0
PPM1F	G>G/A	22	22285632	het	63	missense_variant	260	A/V	tolerated(1)	benign(0.038)	rs61730100	1
TPTE	T>T/G	21	10910311	het	386	missense_variant	482	Y/S	tolerated(1)	benign(0.003)	rs9996	0
HCK	A>A/T	20	30661605	het	101	missense_variant	105	M/L	tolerated(1)	benign(0.005)	rs55722810	1
RFPL4A	G>G/A	19	56274453	het	442	missense_variant	259	G/E	tolerated(1)	benign(0.006)	rs75239380	0
GPR32	A>A/C	19	51274851	het	140	missense_variant	332	T/P	tolerated(1)	benign(0)	rs201404376	0
CYP2A7	C>C/A	19	41386486	het	50	missense_variant	131	A/S	tolerated(1)	benign(0.003)	rs3815708	0
ZNF780B	C>C/T	19	40541039	het	243	missense_variant	576	R/Q	tolerated(1)	benign(0.003)	rs61737024	2
ZNF493	G>G/A	19	21606390	het	31	missense_variant	310	G/E	tolerated(1)	benign(0.001)	rs112511886	1
PBX4	C>C/T	19	19674370	het	26	missense_variant	327	R/Q	tolerated(1)	benign(0.001)	rs141765874	1
PLIN4	T>T/C	19	4511730	het	138	missense_variant	734	I/V	tolerated(1)	benign(0)	rs62115190	0
PLIN4	C>C/T	19	4511283	het	146	missense_variant	883	A/T	tolerated(1)	benign(0.002)	rs80238130	0
PSMA8	A>A/G	18	23731818	het	45	missense_variant	82	I/V	tolerated(1)	benign(0)		0
KRTAP4-6	T>T/A	17	39296412	het	50	missense_variant	110	S/C	tolerated(1)	unknown(0)	rs200470462	0
KRTAP4-6	A>A/G	17	39296361	het	63	missense_variant	127	S/P	tolerated(1)	unknown(0)	rs146882220	0
KRTAP4-11	T>T/G	17	39274364	het	79	missense_variant	68	R/S	tolerated(1)	unknown(0)	rs425784	0
KCNJ12	C>C/A	17	21319860	het	86	missense_variant	402	D/E	tolerated(1)	benign(0.008)	rs2917720	0
KCNJ12	G>G/A	17	21319369	het	445	missense_variant	239	E/K	tolerated(1)	benign(0.232)	rs77048459	0
OR1D5	C>C/G	17	2966032	het	29	missense_variant	290	R/S	tolerated(1)	benign(0)	rs202049082	0
CES1	T>T/C	16	55862791	het	186	missense_variant	50	I/V	tolerated(1)	benign(0.001)	rs114632091	0
SCNN1G	G>G/A	16	23200921	het	143	missense_variant	183	G/S	tolerated(1)	benign(0.003)	rs5736	1
NOMO1	T>T/A	16	14969016	het	50	missense_variant	726	N/K	tolerated(1)	benign(0)	rs79100490	0
SCAPER	T>T/C	15	77067396	het	89	missense_variant	279	T/A	tolerated(1)	benign(0.01)	rs142461932	0.09

EMC7	T>T/A	15	34380331	het	18	missense_variant	167	M/L	tolerated(1)	benign(0.001)	rs140117138	0.37
AHNAK2	T>T/C	14	105415229	het	87	missense_variant	2187	M/V	tolerated(1)	benign(0.058)	rs10134675	0
PAPLN	A>A/G	14	73726151	het	104	missense_variant	601	H/R	tolerated(1)	benign(0.001)	rs17182244	2
SRGAP1	G>G/A	12	64485153	het	108	missense_variant	512	V/I	tolerated(1)	benign(0.001)	rs74691643	2
CNTN5	A>A/G	11	99690461	het	131	missense_variant	81	N/S	tolerated(1)	benign(0.006)	rs10893933	0
CNTN5	T>T/G	11	99690286	het	55	missense_variant	23	S/A	tolerated(1)	benign(0)	rs10790978	0
OR9G1	T>T/C	11	56468720	het	175	missense_variant	286	L/P	tolerated(1)	benign(0)	rs66943455	0
OR9G1	G>G/A	11	56468212	het	134	missense_variant	117	V/M	tolerated(1)	benign(0.001)	rs591369	0
OR4C3	A>A/T	11	48346961	het	181	missense_variant	157	N/Y	tolerated(1)	benign(0)	rs75493089	0
MUC6	T>T/G	11	1018116	het	134	missense_variant	1562	N/T	tolerated(1)	unknown(0)	rs10902269	0
MUC6	A>A/G	11	1017858	het	324	missense_variant	1648	M/T	tolerated(1)	unknown(0)	rs79583615	0
MUC6	C>C/G	11	1017498	het	423	missense_variant	1768	S/T	tolerated(1)	unknown(0)	rs76707565	0
MUC6	G>G/C	11	1017384	het	410	missense_variant	1806	T/S	tolerated(1)	benign(0.05)	rs34095361	0
MUC6	G>G/C	11	1017302	het	297	missense_variant	1833	H/Q	tolerated(1)	benign(0.025)	rs35330958	0
MUC6	C>C/T	11	1016665	het	231	missense_variant	2046	V/I	tolerated(1)	benign(0.217)	rs77438942	0
FRG2B	T>T/G	10	135438929	het	365	missense_variant	171	I/L	tolerated(1)	benign(0)	rs201901202	0
GPRIN2	T>T/C	10	46999151	het	93	missense_variant	91	W/R	tolerated(1)	benign(0)	rs3127820	0
OBP2A	T>T/A	9	138439086	het	23	missense_variant	90	F/Y	tolerated(1)	benign(0.002)	rs492193	2
CBWD6	G>G/T	9	69238234	het	15	missense_variant	220	L/I	tolerated(1)	benign(0.005)	rs114804965	0
PRSS3	G>G/A	9	33798574	het	107	missense_variant	239	S/N	tolerated(1)	benign(0.002)	rs201773718	0
TUSC1	T>T/C	9	25678196	het	22	missense_variant	42	S/G	tolerated(1)	benign(0.336)	rs72631813	0
IFNA14	T>T/C	9	21239504	het	125	missense_variant	144	K/R	tolerated(1)	benign(0.002)	rs140823004	0
IFNA16	C>C/G	9	21216908	het	141	missense_variant	133	A/P	tolerated(1)	benign(0)	rs28368163	2
NOBOX	C>C/T	7	144096158	het	66	missense_variant	452	D/N	tolerated(1)	benign(0)	rs112190116	0.37
OR2A1	C>C/G	7	144015720	het	10	missense_variant	168	S/C	tolerated(1)	benign(0.001)	rs201568948	0
OR2A1	A>A/G	7	144015434	het	84	missense_variant	73	T/A	tolerated(1)	benign(0)	rs141871720	0
STEAP1B	C>C/T	7	22533452	het	120	missense_variant	30	E/K	tolerated(1)	benign(0.002)	rs201338533	0
SERINC1	T>T/C	6	122773119	het	102	missense_variant	225	S/G	tolerated(1)	benign(0)	rs17260829	2
MUC7	T>T/C	4	71347207	het	18	missense_variant	249	L/P	tolerated(1)	unknown(0)	rs78564790	0
C4orf40	G>G/C	4	71024463	het	56	missense_variant	165	G/A	tolerated(1)	unknown(0)	rs142405912	1
HGFAC	A>A/G	4	3449915	het	29	missense_variant	566	K/R	tolerated(1)	benign(0.191)	rs114303452	1
FGFRL1	G>G/A	4	1016254	het	64	missense_variant	115	V/I	tolerated(1)	benign(0.001)		0
MUC20	G>G/C	3	195447886	het	52	missense_variant	3	C/S	tolerated(1)	benign(0)	rs7627924	0
KBTBD12	A>A/C	3	127642561	het	176	missense_variant	219	Q/H	tolerated(1)	benign(0.005)	rs148151101	0.46
ZNF620	C>C/T	3	40553962	het	123	missense_variant	74	P/L	tolerated(1)	benign(0)		0
ABCA12	G>G/C	2	215843127	het	123	missense_variant	1681	Q/E	tolerated(1)	benign(0.026)		0
ALS2	T>T/C	2	202626437	het	101	missense_variant	94	I/V	tolerated(1)	benign(0)	rs3219154	2
COL5A2	A>A/G	2	189931144	het	34	missense_variant	512	V/A	tolerated(1)	benign(0.181)	rs35852101	1
PLA2R1	C>C/T	2	160798408	het	116	missense_variant	1425	G/S	tolerated(1)	benign(0.006)	rs201567578	0.05
RIF1	A>A/G	2	152311612	het	143	missense_variant	850	M/V	tolerated(1)	benign(0.425)	rs61750444	2
CCDC142	G>G/A	2	74701836	het	94	missense_variant	690	P/L	tolerated(1)	benign(0.029)	rs116238920	1
OR2T34	G>G/A	1	248737293	het	89	missense_variant	256	L/F	tolerated(1)	benign(0.002)	rs200427293	0
OR2T2	C>C/T	1	248616408	het	502	missense_variant	104	L/F	tolerated(1)	benign(0.064)	rs73147579	0
IGFN1	A>A/G	1	201179050	het	47	missense_variant	1677	N/D	tolerated(1)	benign(0)	rs1722732	0
NBPF14,PDE4	C>C/T	1	144952207	het	257	missense_variant	171	R/K	tolerated(1)	benign(0.004)	rs3121544	0
HNRNPCL1	C>C/T	1	12907802	het	155	missense_variant	114	G/D	tolerated(1)	benign(0)	rs138087690	0
HNRNPCL1	T>T/C	1	12907284	het	41	missense_variant	287	T/A	tolerated(1)	benign(0.001)	rs149796618	0
N4BP2	C>C/T	4	40122453	het	167	missense_variant	908	P/S	tolerated(0.99)	benign(0.006)	rs78417585	1
RFPL4A	T>T/A	19	56274213	het	109	missense_variant	179	V/E	tolerated(0.97)	benign(0.021)	rs147035425	0
MUC6	C>C/G	11	1018483	het	136	missense_variant	1440	E/Q	tolerated(0.96)	unknown(0)	rs78265558	0
MUC6	G>G/A	11	1018456	het	109	missense_variant	1449	P/S	tolerated(0.96)	unknown(0)	rs79920422	0
MUC6	T>T/C	11	1016713	het	237	missense_variant	2030	T/A	tolerated(0.96)	benign(0.013)	rs76406481	0
NBPF14,PDE4	T>T/C	1	144854581	het	89	missense_variant	2297	T/A	tolerated(0.96)	benign(0)	rs78371650	0
UMODL1	G>G/A	21	43539287	het	87	missense_variant	976	V/I	tolerated(0.95)	benign(0.004)	rs118104890	2
TIMM23	C>C/A	10	51623174	het	23	missense_variant	14	G/V	tolerated(0.94)	unknown(0)	rs141118546	0
LRRC56	C>C/A	11	550192	het	71	missense_variant	182	Q/K	tolerated(0.93)	possibly_damaging(0.473)	rs61736743	1
MEGF6	G>G/T	1	3418370	het	73	missense_variant	768	D/E	tolerated(0.93)	benign(0.019)		0
SMYD4	C>C/T	17	1690118	het	94	missense_variant	624	G/R	tolerated(0.92)	benign(0)	rs202113499	0
IFNA16	A>A/G	9	21216910	het	144	missense_variant	132	I/T	tolerated(0.91)	benign(0.001)	rs28368162	2
NBPF14,NBPF	G>G/C	1	145293498	het	19	missense_variant	31	K/N	tolerated(0.91)	benign(0.007)	rs4409714	0
MUC6	G>G/C	11	1017381	het	425	missense_variant	1807	T/S	tolerated(0.89)	benign(0.05)	rs34912894	0
ARHGEF10L	G>G/A	1	18023444	het	57	missense_variant	1137	E/K	tolerated(0.87)	benign(0.038)	rs142160248	0
CNTN5	T>T/G	11	99690428	het	174	missense_variant	70	L/R	tolerated(0.86)	benign(0)	rs7125822	0
MUC6	C>C/A	11	1016704	het	256	missense_variant	2033	A/S	tolerated(0.86)	benign(0.105)	rs75826443	0
LILRA6	T>T/C	19	54746081	het	233	missense_variant	59	Q/R	tolerated(0.84)	benign(0)	rs28445220	0
KIAA1377	G>G/A	11	101829006	het	50	missense_variant	205	R/K	tolerated(0.83)	benign(0.001)	rs61743062	1
RP1L1	C>C/T	8	10470230	het	36	missense_variant	460	G/S	tolerated(0.82)	benign(0.389)		0
FCGBP	T>T/G	19	40392585	het	64	missense_variant	2640	E/A	tolerated(0.81)	benign(0.032)	rs79253448	0
FAM205A	G>G/A	9	34724059	het	52	missense_variant	1060	H/Y	tolerated(0.81)	possibly_damaging(0.44)	rs117821239	0
MVD	G>G/T	16	88718944	het	67	missense_variant	398	P/T	tolerated(0.8)	benign(0.042)	rs184535367	0.05
MUC6	G>G/C	11	1017112	het	557	missense_variant	1897	P/A	tolerated(0.8)	benign(0.064)	rs77753732	0
TCP10	C>C/A	6	167786750	het	177	missense_variant	296	K/N	tolerated(0.8)	benign(0)	rs143094588	0
LGI2	T>T/C	4	25005356	het	43	missense_variant	452	Q/R	tolerated(0.8)	benign(0.013)	rs2232027	2
RBM43	C>C/G	2	152112161	het	154	missense_variant	34	V/L	tolerated(0.8)	benign(0)	rs147060862	2
ZNF668	G>G/A	16	31072526	het	102	missense_variant	598	P/S	tolerated(0.79)	benign(0)		0
DEAF1	G>G/C	11	644614	het	78	missense_variant	545	A/G	tolerated(0.79)	benign(0.002)	rs34114147	1
ALG5	T>T/C	13	37569134	het	21	missense_variant	93	K/E	tolerated(0.78)	benign(0.003)		0
TRAPPC8	T>T/C	18	29426714	het	42	missense_variant	1268	I/V	tolerated(0.77)	benign(0.002)	rs146392001	0.05
OR9G1	A>A/C	11	56468452	het	132	missense_variant	197	M/L	tolerated(0.77)	benign(0.001)	rs79970235	0
SDK2	G>G/C	17	71418561	het	193	missense_variant	637	T/S	tolerated(0.76)	benign(0.092)		0
CBLB	G>G/C	3	105421095	het	87	missense_variant	601	T/S	tolerated(0.75)	benign(0.003)		0
GNL3L	G>G/T	X	54584923	het	66	missense_variant	501	A/S	tolerated(0.74)	benign(0.019)	rs141269966	0.06
ERCC4	G>G/A	16	14042100	het	125	missense_variant	883	E/K	tolerated(0.74)	benign(0)	rs201652412	0
KIAA0947	A>A/G	5	5476177	het	26	missense_variant	2169	I/V	tolerated(0.74)	benign(0.01)		0
NKX2-2	C>C/G	20	21493018	het	47	missense_variant	122	G/A	tolerated(0.73)	benign(0.057)	rs8192563	1
IGFN1	A>A/G	1	201178904	het	72	missense_variant	1628	E/G	tolerated(0.73)	benign(0)	rs201227267	0
ZNF18	A>A/G	17	11881498	het	87	missense_variant	476	F/L	tolerated(0.72)	benign(0.003)	rs62621364	1
DNAH7	C>C/G	2	196877525	het	28	missense_variant	325	E/D	tolerated(0.72)	benign(0.003)		0
TM9SF4	G>G/A	20	30729619	het	140	missense_variant	150	R/Q	tolerated(0.71)	benign(0.082)	rs117822199	1
FAM186A	T>T/A	12	50745783	het	71	missense_variant	1611	Q/L	tolerated(0.71)	benign(0)		0
SYNE1	C>C/T	6	152711428	het	45	missense_variant	2722	V/I	tolerated(0.71)	benign(0.001)	rs151091241	0.37
PRAMEF22	C>C/T	1	13036587	het	31	missense_variant	220	P/L	tolerated(0.71)	benign(0.003)	rs77788641	0
SAMD7	T>T/C	3	169646266	het	383	missense_variant	314	I/T	tolerated(0.7)	benign(0)	rs75972343	1
ITGB4	G>G/T	17	73728300	het	109	missense_variant	478	Q/H	tolerated(0.69)	benign(0)	rs8079267	0.14
PDPR	A>A/G	16	70154480	het	198	missense_variant	29	T/A	tolerated(0.69)	benign(0.001)	rs200469748	0
AKAP11	C>C/G	13	42875626	het	114	missense_variant	915	T/S	tolerated(0.69)	benign(0.004)		0
PINX1	G>G/T	8	10623031	het	131	missense_variant	289	F/L	tolerated(0.69)	benign(0)		0
CLCA1	A>A/G	1	86954743	het	127	missense_variant	416	N/S	tolerated(0.69)	benign(0.023)		0
MAGEC1	C>C/G	X	140993852	het	85	missense_variant	221	T/S	tolerated(0.68)	benign(0.004)	rs176038	0
GNAL	T>T/C	18	11753853	het	117	missense_variant	178	I/T	tolerated(0.68)	benign(0.009)		0
SYNE2	A>A/G	14	64685212	het	62	missense_variant	6547	I/V	tolerated(0.67)	benign(0.005)	rs45453691	1
OR4C3	T>T/G	11	48346604	het	193	missense_variant	38	F/V	tolerated(0.67)	benign(0.002)	rs75647397	0
MUC6	T>T/A	11	1017981	het	142	missense_variant	1607	H/L	tolerated(0.67)	unknown(0)	rs61869010	0
NBPF14,NOT	T>T/C	1	145273345	het	921	missense_variant	67	S/P	tolerated(0.66)	benign(0.238)	rs75987820	0
CUBN	A>A/G	10	16957036	het	39	missense_variant	2449	M/T	tolerated(0.65)	benign(0.001)	rs41301097	0
ITPR3	A>A/C	6	33652456	het	155	missense_variant	1710	I/L	tolerated(0.65)	benign(0.001)	rs142457410	0.09
PIGK	C>C/A	1	77620300	het	14	missense_variant	274	V/L	tolerated(0.65)	benign(0.039)		0
CEP89	C>C/T	19	33444576	het	265	missense_variant	146	R/Q	tolerated(0.64)	benign(0.002)	rs74504201	0
OR13C5	T>T/C	9	107361052	het	198	missense_variant	215	I/V	tolerated(0.64)	benign(0.006)		0
PRSS1	G>G/A	7	142460369	het	134	missense_variant	181	S/N	tolerated(0.64)	benign(0)	rs201719096	0
TPX2	C>C/T	20	30385310	het	172	missense_variant	646	S/L	tolerated(0.62)	benign(0.007)		0
ZADH2	C>C/A	18	72913537	het	146	missense_variant	323	C/F	tolerated(0.62)	benign(0)	rs17056661	2
MON1B	C>C/A	16	77228967	het	74	missense_variant	404	A/D	tolerated(0.62)	benign(0.026)		0
ATP6V0A2	A>A/G	12	124242557	het	154	missense_variant	850	N/S	tolerated(0.62)	benign(0)	rs75279884	1
PLEKHG4B	G>G/A	5	140622	het	67	missense_variant	67	R/Q	tolerated(0.62)	benign(0)	rs151163016	0
PLEKHH2	C>C/T	2	43924417	het	116	missense_variant	204	P/S	tolerated(0.62)	benign(0.001)	rs142153423	0.23
NBPF14,PDE4	C>C/T	1	144854598	het	71	missense_variant	2291	R/Q	tolerated(0.62)	benign(0.001)	rs3863691	0
MAGEC1	T>T/C	X	140993864	het	72	missense_variant	225	F/S	tolerated(0.61)	benign(0.343)	rs34836042	0
SURF2	C>C/T	9	136228007	het	181	missense_variant	255	P/S	tolerated(0.61)	probably_damaging(0.904)	rs61751475	1
IL7R	G>G/A	5	35875627	het	215	missense_variant	272	V/I	tolerated(0.61)	benign(0.007)		0
PRAMEF1	G>G/A	1	12854414	het	62	missense_variant	213	R/H	tolerated(0.61)	benign(0.003)	rs1063769	0
MUC6	C>C/G	11	1017691	het	497	missense_variant	1704	A/P	tolerated(0.6)	unknown(0)	rs77359624	0
DNA2	T>T/C	10	70229874	het	235	missense_variant	41	T/A	tolerated(0.6)	benign(0)		0
LYZL2	G>G/C	10	30918549	het	147	missense_variant	29	A/G	tolerated(0.6)	benign(0)	rs201069868	0
ZYG11A	A>A/G	1	53347210	het	124	missense_variant	606	N/S	tolerated(0.6)	benign(0.002)	rs74990705	1
TRAPPC9	C>C/T	8	141461333	het	223	missense_variant	145	R/Q	tolerated(0.59)	benign(0.378)	rs147182402	0
PRRT4	G>G/A	7	127991262	het	49	missense_variant	783	P/L	tolerated(0.59)	benign(0.001)	rs75191863	2
UPK1B	G>G/C	3	118906795	het	205	missense_variant	81	M/I	tolerated(0.59)	benign(0.001)		0
ZNF217	T>T/C	20	52198138	het	126	missense_variant	410	M/V	tolerated(0.58)	benign(0.004)	rs6068589	0.14
CCR7	T>T/C	17	38715186	het	58	missense_variant	7	M/V	tolerated(0.58)	benign(0.001)	rs2228015, rs45	2
ADAMTSL1	G>G/A	9	18889685	het	56	missense_variant	1528	A/T	tolerated(0.58)	benign(0.002)		0
IL31RA	G>G/A	5	55192204	het	100	missense_variant	267	V/I	tolerated(0.58)	benign(0.018)		0
ZNF428,SRRM	T>T/C	19	44117582	het	31	missense_variant	437	C/R	tolerated(0.57)	benign(0)	rs10410000	0
GLCE	A>A/G	15	69548338	het	149	missense_variant	65	M/V	tolerated(0.57)	benign(0)	rs12440300	2
DNAH12	G>G/T	3	57494915	het	39	missense_variant	165	P/Q	tolerated(0.57)	unknown(0)	rs115576589	1
AHDC1	C>C/T	1	27874627	het	130	missense_variant	1334	G/S	tolerated(0.57)	benign(0.04)	rs143951781	0.09
PLXNB2	T>T/C	22	50727965	het	45	missense_variant	350	Q/R	tolerated(0.56)	benign(0.074)		0
PFAS	G>G/T	17	8172277	het	186	missense_variant	1238	V/L	tolerated(0.56)	benign(0.003)	rs145272400	0

TPSD1	A>A/G	16	1306971	het	51	missense_variant	143	H/R	tolerated(0.56)	benign(0.02)	rs72775466	0
DNAAF2	C>C/T	14	50100073	het	188	missense_variant	599	A/T	tolerated(0.56)	probably_damaging(0.98)		0
MPHOSPH9	G>G/A	12	123687300	het	227	missense_variant	399	T/I	tolerated(0.56)	probably_damaging(0.996)		0
PCMTD1	T>T/G	8	52733128	het	108	missense_variant	286	N/T	tolerated(0.56)	benign(0.012)	rs62506083	0
SOWAHC	C>C/G	2	110372581	het	32	missense_variant	172	P/R	tolerated(0.56)	benign(0.147)		0
NBPF14,PDE	C>C/T	1	144952220	het	237	missense_variant	167	A/T	tolerated(0.56)	benign(0.029)	rs139438772	0
TNS4	C>C/T	17	38644859	het	110	missense_variant	268	G/S	tolerated(0.55)	benign(0.009)		0
NID2	T>T/C	14	52486783	het	106	missense_variant	930	I/V	tolerated(0.55)	benign(0.002)	rs144774059	0.05
PRSS3	G>G/A	9	33796673	het	119	missense_variant	82	V/I	tolerated(0.55)	benign(0.13)	rs76740888	0
TCP10L2	G>G/A	6	167591954	het	81	missense_variant	194	R/H	tolerated(0.55)	benign(0.001)	rs200583385	0
TGM2	C>C/A	20	36760894	het	71	missense_variant	542	V/F	tolerated(0.54)	probably_damaging(0.945)	rs2229473, rs111	
POM121	T>T/C	7	72413593	het	101	missense_variant	756	Y/H	tolerated(0.54)	benign(0)	rs71554688	0
POM121L2	C>C/G	6	27279021	het	78	missense_variant	310	G/A	tolerated(0.54)	benign(0)	rs61736094	2
SLX4	G>G/T	16	3639699	het	253	missense_variant	1314	Q/K	tolerated(0.53)	benign(0.174)	rs142040192	0
JPH4	C>C/T	14	24040430	het	131	missense_variant	504	A/T	tolerated(0.53)	benign(0.001)	rs147893967	1
LATS2	G>G/A	13	21563311	het	111	missense_variant	203	A/V	tolerated(0.53)	benign(0)	rs77919685	2
TBX3	C>C/T	12	115109855	het	103	missense_variant	675	A/T	tolerated(0.53)	benign(0.003)		0
TIMM23	T>T/C	10	51623190	het	16	missense_variant	9	N/D	tolerated(0.53)	unknown(0)	rs137912947	0
FAM105B	G>G/T	5	14681593	het	194	missense_variant	115	Q/H	tolerated(0.53)	benign(0.015)	rs147790160	0.32
CD6	G>G/A	11	60776039	het	128	missense_variant	168	G/D	tolerated(0.52)	benign(0.097)		0
KMT2C	T>T/A	7	151970856	het	714	missense_variant	316	T/S	tolerated(0.52)	benign(0.378)	rs10454320	0
C1orf87	T>T/C	1	60506673	het	102	missense_variant	158	D/G	tolerated(0.52)	benign(0.003)	rs145230176	0.05
KCNJ12	G>G/A	17	21319171	het	354	missense_variant	173	D/N	tolerated(0.51)	probably_damaging(1)	rs73313922	0
KCNJ12	G>G/A	17	21319007	het	244	missense_variant	118	R/Q	tolerated(0.51)	benign(0.116)	rs1657740	0
RPP30	C>C/T	10	92631775	het	213	missense_variant	11	A/V	tolerated(0.51)	benign(0.071)	rs41286916	0.23
ANGPT1	C>C/T	8	108296964	het	144	missense_variant	384	R/Q	tolerated(0.51)	benign(0.003)	rs146465357	0.23
MUC20	C>C/T	3	195452799	het	83	missense_variant	271	T/I	tolerated(0.51)	benign(0.308)	rs2550232	0
TARBP1	A>A/G	1	234553976	het	8	missense_variant	1187	F/L	tolerated(0.51)	possibly_damaging(0.599)		0
TINAGL1	C>C/A	1	32052529	het	38	missense_variant	451	F/L	tolerated(0.51)	benign(0.077)	rs113639076	1
FAM184B	T>T/C	4	17690072	het	25	missense_variant	524	Q/R	tolerated(0.5)	benign(0.082)	rs61741063	1
NBPF14,PDE	A>A/G	1	144871782	het	42	missense_variant	1727	L/P	tolerated(0.5)	benign(0)	rs1778158	0
ZSWIM1	G>G/A	20	44511533	het	102	missense_variant	101	R/Q	tolerated(0.49)	benign(0.001)	rs3746500	2
FAM173B	A>A/G	5	10236693	het	99	missense_variant	114	V/A	tolerated(0.49)	benign(0.083)	rs17360625	2
A4GNT	C>C/T	3	137849888	het	161	missense_variant	71	V/I	tolerated(0.49)	possibly_damaging(0.679)	rs79791762	2
PRAMEF11	T>T/C	1	12887549	het	40	missense_variant	103	E/G	tolerated(0.49)	benign(0)	rs2994114	0
CLEC18C	T>T/C	16	70211226	het	87	missense_variant	100	L/P	tolerated(0.48)	benign(0)	rs150357848	0
POM121	G>G/A	7	72413581	het	101	missense_variant	752	V/I	tolerated(0.48)	benign(0)	rs71554687	0
KLHL26	G>G/A	19	18778815	het	149	missense_variant	203	R/H	tolerated(0.47)	benign(0.001)		0
OR8U1	T>T/G	11	56143699	het	283	missense_variant	200	F/L	tolerated(0.47)	benign(0.047)	rs4990121	0
WDR34	C>C/T	9	131397448	het	73	missense_variant	302	G/R	tolerated(0.47)	benign(0.002)		0
MUC6	C>C/T	11	1016776	het	423	missense_variant	2009	V/I	tolerated(0.46)	possibly_damaging(0.466)	rs33988517	0
PDGFB	G>G/C	22	39621798	het	129	missense_variant	219	P/R	tolerated(0.45)	benign(0.053)	rs200444646	0.05
AHNAK2	A>A/G	14	105412009	het	44	missense_variant	3260	M/T	tolerated(0.45)	benign(0)	rs28714612	0
CHP2	G>G/A	16	23768529	het	102	missense_variant	141	R/H	tolerated(0.44)	probably_damaging(0.975)	rs112597134	0.05
C15orf39	C>C/A	15	75500586	het	82	missense_variant	733	Q/K	tolerated(0.44)	unknown(0)		0
TSPEAR,KRT	G>G/A	21	45971152	het	59	missense_variant	64	P/S	tolerated(0.43)	possibly_damaging(0.731)	rs199607801	0
VRTN	G>G/A	14	74823701	het	153	missense_variant	72	R/Q	tolerated(0.43)	benign(0.003)	rs45593432	0.18
TESPA1	T>T/C	12	55356823	het	145	missense_variant	287	I/V	tolerated(0.43)	benign(0.106)	rs151024475	1
USP35	C>C/T	11	77911745	het	67	missense_variant	363	S/L	tolerated(0.43)	unknown(0)	rs147270370	0.27
SVEP1	C>C/T	9	113169968	het	187	missense_variant	2638	D/N	tolerated(0.43)	benign(0.019)	rs150389025	0.18
PABPC1	C>C/T	8	101718968	het	43	missense_variant	505	V/I	tolerated(0.43)	benign(0.002)	rs62513922	0
TAB3	A>A/C	X	30852209	het	122	missense_variant	650	V/G	tolerated(0.42)	benign(0.002)		0
DSC3	C>C/T	18	28588084	het	29	missense_variant	521	D/N	tolerated(0.42)	benign(0.214)	rs186116908	0.14
CCDC88C	C>C/G	14	91739108	het	45	missense_variant	1983	G/A	tolerated(0.42)	benign(0.079)	rs45542736	1
CLSTN3	G>G/T	12	7283287	het	29	missense_variant	15	A/S	tolerated(0.42)	unknown(0)	rs145190321	0.46
MUC6	G>G/T	11	1017421	het	353	missense_variant	1794	P/T	tolerated(0.42)	benign(0.336)	rs35549382	0
DOCK8	G>G/A	9	312134	het	86	missense_variant	237	E/K	tolerated(0.42)	benign(0.185)	rs11789099	1
EPPK1	G>G/C	8	144940230	het	137	missense_variant	2398	L/V	tolerated(0.42)	benign(0)	rs7005697	0
C8orf47	G>G/A	8	99101507	het	107	missense_variant	88	V/I	tolerated(0.42)	benign(0.252)	rs118038112	1
PHTF2	C>C/A	7	77549673	het	127	missense_variant	251	T/N	tolerated(0.42)	benign(0.061)	rs201907528	0.05
GOLGB1	A>A/G	3	121415642	het	190	missense_variant	1243	I/T	tolerated(0.42)	benign(0.009)	rs139646586	0.05
ROBO2	C>C/A	3	75986663	het	42	missense_variant	7	R/S	tolerated(0.42)	unknown(0)	rs12171318	0
CYP2D6	C>C/T	22	42523505	het	110	missense_variant	373	G/S	tolerated(0.41)	benign(0.164)	rs150552908	2
CHRNA4	G>G/A	20	61981411	het	28	missense_variant	451	P/L	tolerated(0.41)	benign(0.006)	rs55915440	0.05
CHAC1	A>A/G	15	41247629	het	251	missense_variant	151	K/R	tolerated(0.41)	benign(0.104)	rs147487857	1
MUC20	C>C/T	3	195452783	het	70	missense_variant	266	P/S	tolerated(0.41)	probably_damaging(0.991)	rs199620417	0
WFDC3	C>C/A	20	44417593	het	135	missense_variant	63	R/L	tolerated(0.4)	benign(0.02)	rs73122754	3
SPAG5	G>G/T	17	26911215	het	104	missense_variant	789	Q/K	tolerated(0.4)	benign(0.013)	rs150901596	0.09
SLC15A1	G>G/T	13	99356607	het	68	missense_variant	451	T/N	tolerated(0.4)	benign(0.01)	rs8187838	1
DOCK5	G>G/A	8	25203071	het	147	missense_variant	900	E/K	tolerated(0.4)	benign(0.02)	rs141362225	0
ZNF787	C>C/G	19	56599455	het	16	missense_variant	362	E/D	tolerated(0.39)	unknown(0)	rs202243737	0
CEP89	T>T/C	19	33444556	het	345	missense_variant	153	S/G	tolerated(0.39)	benign(0)	rs73579706	0
PRH1	G>G/T	12	11035274	het	80	missense_variant	42	L/I	tolerated(0.39)	benign(0.035)	rs28607516	0
OGDHL	C>C/G	10	50952101	het	66	missense_variant	600	M/I	tolerated(0.39)	benign(0)	rs36032010	1
ZP3	A>A/G	7	76069881	het	149	missense_variant	338	H/R	tolerated(0.39)	benign(0.004)	rs2906998	0
ECEL1	C>C/G	2	233348851	het	57	missense_variant	423	E/Q	tolerated(0.39)	possibly_damaging(0.787)	rs41265123	1
CERS3	T>T/C	15	100943045	het	109	missense_variant	342	D/G	tolerated(0.38)	benign(0.021)	rs1023783	1
TBX3	T>T/G	12	115120788	het	86	missense_variant	73	E/A	tolerated(0.38)	possibly_damaging(0.85)		0
CACNA1B	A>A/G	9	140943730	het	185	missense_variant	1225	I/V	tolerated(0.38)	benign(0.425)	rs200164302	0
YIPF5	G>G/A	5	143545052	het	92	missense_variant	76	A/V	tolerated(0.38)	benign(0.002)	rs35429531	1
IL6ST	C>C/T	5	55248135	het	64	missense_variant	499	V/I	tolerated(0.38)	benign(0.007)	rs34417936	1
ATR	C>C/A	3	142253996	het	76	missense_variant	1291	A/S	tolerated(0.38)	possibly_damaging(0.735)		0
CLEC18A	C>C/G	16	69988463	het	49	missense_variant	148	T/S	tolerated(0.37)	benign(0.01)	rs78531533	0
ZP3	A>A/G	7	76069886	het	147	missense_variant	340	M/V	tolerated(0.37)	benign(0)	rs2906997	0
DCBLD2	G>G/C	3	98619974	het	26	missense_variant	66	A/G	tolerated(0.37)	possibly_damaging(0.51)	rs202016309	0
C1orf87	G>G/C	1	60505771	het	135	missense_variant	189	P/A	tolerated(0.37)	benign(0.011)	rs11585228	0.05
CILP2	C>C/G	19	19655657	het	124	missense_variant	768	A/G	tolerated(0.36)	benign(0.097)	rs183579609	0.27
MUC6	A>A/G	11	1017514	het	453	missense_variant	1763	S/P	tolerated(0.36)	unknown(0)	rs79037833	0
ZBTB24	C>C/T	6	109787476	het	119	missense_variant	558	D/N	tolerated(0.36)	benign(0.005)	rs61731736	0.23
PCDHB11	G>G/A	5	140580941	het	15	missense_variant	532	G/S	tolerated(0.36)	possibly_damaging(0.528)	rs138686663	0
C1QA	C>C/T	1	22964156	het	71	missense_variant	16	S/L	tolerated(0.36)	benign(0.019)		0
SPINT4	C>C/A	20	44351095	het	76	missense_variant	30	A/E	tolerated(0.35)	benign(0.136)	rs16990631	2
MADCAM1	C>C/A	19	501738	het	14	missense_variant	246	P/Q	tolerated(0.35)	unknown(0)	rs1063736	0
ATP9B	C>C/T	18	76856499	het	231	missense_variant	48	A/V	tolerated(0.35)	benign(0.09)	rs181100780	0.05
RPAP1	G>G/A	15	41829178	het	118	missense_variant	49	P/L	tolerated(0.35)	benign(0.001)	rs112536229	1
KMT2C	G>G/A	7	151945204	het	248	missense_variant	772	S/L	tolerated(0.35)	benign(0.34)	rs4024453	0
ETAA1	C>C/T	2	67631958	het	84	missense_variant	715	P/L	tolerated(0.35)	possibly_damaging(0.44)	rs3770656	1
CTSE	T>T/C	1	206318378	het	146	missense_variant	46	W/R	tolerated(0.35)	benign(0.171)		0
HMCN1	G>G/A	1	186024643	het	196	missense_variant	2327	M/I	tolerated(0.35)	benign(0.006)	rs12067376	1
ZNF630	C>C/T	X	47919855	het	99	missense_variant	71	S/N	tolerated(0.34)	possibly_damaging(0.51)	rs41297342	2
KRT40	A>A/G	17	39140507	het	50	missense_variant	7	S/P	tolerated(0.34)	benign(0)	rs201719050	0
PDGFA	G>G/C	7	552059	het	71	missense_variant	65	A/G	tolerated(0.34)	benign(0.071)		0
TBL2	C>C/G	7	72992858	het	57	missense_variant	8	E/Q	tolerated(0.33)	probably_damaging(0.933)	rs76029572	2
OTUD4	G>G/A	4	146059189	het	260	missense_variant	848	P/L	tolerated(0.33)	benign(0.097)	rs149518451	0.05
CPZ	G>G/A	4	8621172	het	110	missense_variant	596	R/Q	tolerated(0.33)	benign(0.002)	rs61734020	2
SH3BP1	C>C/T	22	38039746	het	84	missense_variant	190	T/M	tolerated(0.32)	benign(0.098)	rs149928566	0.27
CES1	C>C/T	16	55862824	het	240	missense_variant	39	V/I	tolerated(0.32)	benign(0.009)	rs3826192	0
IGDCC3	C>C/T	15	65621828	het	59	missense_variant	702	R/Q	tolerated(0.32)	benign(0.003)	rs140821348	0
TBX3	A>A/G	12	115109777	het	164	missense_variant	701	S/P	tolerated(0.32)	benign(0.412)		0
DAB2	G>G/A	5	39382877	het	124	missense_variant	395	P/L	tolerated(0.32)	benign(0.006)	rs186305041	0
PLXNB1	G>G/A	3	48463856	het	37	missense_variant	435	P/S	tolerated(0.32)	benign(0.002)	rs142527941	1
SPZ1	A>A/G	5	79616278	het	11	missense_variant	82	N/D	tolerated(0.31)	possibly_damaging(0.767)	rs139471643	1
FAM208A	C>C/T	3	56667640	het	177	missense_variant	1060	R/Q	tolerated(0.31)	probably_damaging(0.947)	rs139788090	0.05
PTCH2	C>C/T	1	45294013	het	85	missense_variant	555	R/Q	tolerated(0.31)	benign(0.012)		0
RSPO4	T>T/G	20	944649	het	41	missense_variant	175	H/P	tolerated(0.3)	benign(0)	rs61740632	2
GTF3C6	T>T/C	6	111288941	het	132	missense_variant	197	I/T	tolerated(0.3)	benign(0.001)	rs41289884	2
SDHA	A>A/T	5	223646	het	93	missense_variant	38	D/V	tolerated(0.3)	benign(0.001)	rs34635677	1
URB2	A>A/G	1	229770671	het	97	missense_variant	104	N/S	tolerated(0.3)	probably_damaging(0.966)		0
ZNF407	A>A/G	18	72343156	het	220	missense_variant	61	S/G	tolerated(0.29)	benign(0.023)	rs75994611	1
GGA3,MRPS	C>C/T	17	73258028	het	100	missense_variant	16	A/V	tolerated(0.29)	benign(0.001)	rs148590649	0.32
AK9	T>T/C	6	109835565	het	8	missense_variant	1381	I/V	tolerated(0.29)	benign(0.044)		0
ARHGEF38	A>A/C	4	106510408	het	71	missense_variant	67	K/T	tolerated(0.29)	probably_damaging(0.994)		0
PADI4	G>G/C	1	17662705	het	80	missense_variant	131	R/T	tolerated(0.29)	benign(0.364)	rs12733102	1
CYLC1	G>G/C	X	83116204	het	71	missense_variant	5	R/T	tolerated(0.28)	probably_damaging(0.991)	rs142474490	1
PCK2	C>C/A	14	24566228	het	297	missense_variant	53	R/S	tolerated(0.28)	benign(0.011)	rs201059299	0
FRG2B	C>C/T	10	135438967	het	328	missense_variant	158	R/Q	tolerated(0.28)	benign(0.004)	rs145018822	0
ARHGEF28	G>G/A	5	73128166	het	130	missense_variant	343	R/H	tolerated(0.28)	benign(0.001)	rs187897891	0.09
ITM2C	C>C/T	2	231729802	het	152	missense_variant	21	A/V	tolerated(0.28)	benign(0.232)	rs149334809	1
IRF2BP2	G>G/A	1	234744889	het	12	missense_variant	118	P/S	tolerated(0.28)	possibly_damaging(0.731)	rs148187914	2
PRELP	A>A/C	1	203455902	het	132	missense_variant	348	N/H	tolerated(0.28)	benign(0.004)	rs9439	1
F8	T>T/C	X	154182223	het	172	missense_variant	616	N/S	tolerated(0.27)	benign(0.005)		0
SEH1L	G>G/A	18	12951878	het	14	missense_variant	46	D/N	tolerated(0.27)	benign(0.017)	rs117978838	1
CSPG4	G>G/A	15	75982072	het	91	missense_variant	445	A/V	tolerated(0.27)	possibly_damaging(0.514)	rs77681934	0
SLIT1	A>A/T	10	98761926	het	8	missense_variant	1452	L/Q	tolerated(0.27)	benign(0.013)		0
C1orf87	C>C/T	1	60505677	het	130	missense_variant	220	R/H	tolerated(0.27)	benign(0.001)	rs150454796	0.05
ZYG11A	A>A/G	1	53326421	het	152	missense_variant	343	M/V	tolerated(0.27)	benign(0.002)	rs61744306	1
CRELD2	G>G/C	22	50318061	het	87	missense_variant	325	E/Q	tolerated(0.26)	possibly_damaging(0.446)	rs113168785	1
BMP7	C>C/T	20	55758936	het	94	missense_variant	267	R/Q	tolerated(0.26)	benign(0.228)	rs143311015	0

SLFN14	G>G/T	17	33880458	het	92	missense_variant	399	Q/K	tolerated(0.26)	benign(0.018)		0
GPD1	G>G/A	12	50500080	het	62	missense_variant	124	E/K	tolerated(0.26)	benign(0.103)	rs34783513	0.05
OR4C3	A>A/C	11	48346588	het	179	missense_variant	32	Q/H	tolerated(0.26)	benign(0.002)	rs75900655	0
IFNA16	G>G/A	9	21216934	het	181	missense_variant	124	T/I	tolerated(0.26)	benign(0.001)	rs28368161	3
PABPC1	C>C/G	8	101718932	het	24	missense_variant	517	V/L	tolerated(0.26)	benign(0.002)	rs62513920	0
GLIS1	C>C/T	1	54060428	het	37	missense_variant	50	G/S	tolerated(0.26)	benign(0.166)	rs143379552	1
SIGLEC14	A>A/G	19	52149716	het	5	missense_variant	72	V/A	tolerated(0.25)	benign(0.005)	rs201390621	0
ACTN4	G>G/A	19	39207742	het	91	missense_variant	310	R/Q	tolerated(0.25)	benign(0.017)	rs112545413	1
MATN2	C>C/T	8	98943205	het	61	missense_variant	56	A/V	tolerated(0.25)	benign(0.233)	rs138635803	0.23
DLX5	G>G/T	7	96650216	het	56	missense_variant	234	S/R	tolerated(0.25)	benign(0.159)	rs35273378	0
DNASE1L2	T>T/C	16	2287496	het	84	missense_variant	146	L/P	tolerated(0.24)	benign(0)		0
PLEKHA1	G>G/A	10	124175436	het	108	missense_variant	177	S/N	tolerated(0.24)	benign(0.031)	rs142473166	0.09
FSD1L	G>G/T	9	108230553	het	27	missense_variant	96	S/I	tolerated(0.24)	possibly_damaging(0.676)	rs41316532	1
ZNF107	G>G/T	7	64166802	het	134	missense_variant	40	E/D	tolerated(0.24)	benign(0.001)	rs73138709	2
NEK1	G>G/C	4	170359267	het	83	missense_variant	911	Q/E	tolerated(0.24)	benign(0.004)	rs6828134	0.41
USPL1	G>G/C	13	31231778	het	165	missense_variant	522	A/P	tolerated(0.23)	probably_damaging(0.915)	rs17609459	2
TMTC1	T>T/G	12	29936444	het	12	missense_variant	81	K/Q	tolerated(0.23)	benign(0.008)		0
PTCHD3	C>C/T	10	27687638	het	109	missense_variant	630	R/Q	tolerated(0.23)	possibly_damaging(0.487)	rs142594066	0
ZP3	G>G/C	7	76069902	het	151	missense_variant	345	R/T	tolerated(0.23)	benign(0.064)	rs1804905	0
RAB23	T>T/C	6	57061300	het	78	missense_variant	116	T/A	tolerated(0.23)	benign(0.206)	rs138803099	0
ZNF141	C>C/G	4	367206	het	74	missense_variant	327	T/S	tolerated(0.23)	benign(0.051)	rs113884485	0
GAL3ST1	C>C/T	22	30953280	het	80	missense_variant	34	V/M	tolerated(0.22)	possibly_damaging(0.534)	rs55674628	1
KIAA1755	G>G/A	20	36868106	het	38	missense_variant	524	T/I	tolerated(0.22)	benign(0.103)	rs11699859	0.27
TRAPPC8	G>G/A	18	29437668	het	150	missense_variant	1008	T/I	tolerated(0.22)	benign(0.003)	rs150390631	0.05
CNOT1	C>C/T	16	58616997	het	171	missense_variant	299	R/Q	tolerated(0.22)	benign(0.404)	rs34830321	1
TBX3	A>A/T	12	115114160	het	75	missense_variant	353	S/T	tolerated(0.22)	benign(0.019)		0
DDX54	G>G/C	12	113623217	het	34	missense_variant	14	R/G	tolerated(0.22)	benign(0.065)	rs141235407	1
PRB4	G>G/T	12	11461769	het	79	missense_variant	50	P/T	tolerated(0.22)	unknown(0)	rs144658455	0
MUC6	A>A/G	11	1017783	het	524	missense_variant	1673	L/P	tolerated(0.22)	unknown(0)	rs36101709	0
SHOX2	G>G/T	3	157816035	het	114	missense_variant	283	H/Q	tolerated(0.22)	possibly_damaging(0.881)		0
TLR5	G>G/A	1	223286129	het	100	missense_variant	82	T/I	tolerated(0.22)	benign(0.041)	rs764535	0.32
CAMTA1	A>A/C	1	7798270	het	87	missense_variant	1304	T/P	tolerated(0.22)	benign(0.054)		0
MADCAM1	C>C/A	19	501714	het	9	missense_variant	238	P/Q	tolerated(0.21)	unknown(0)	rs78071082	0
MAP1A	A>A/G	15	43819450	het	119	missense_variant	1927	K/E	tolerated(0.21)	benign(0.001)	rs147141458	1
ADAMTSL1	C>C/A	9	18777293	het	107	missense_variant	1022	D/E	tolerated(0.21)	probably_damaging(0.98)		0
CDHR3	C>C/A	7	105665004	het	40	missense_variant	752	P/T	tolerated(0.21)	benign(0.006)	rs117406926	0.32
TNS1	G>G/A	2	218713469	het	105	missense_variant	466	R/C	tolerated(0.21)	benign(0.418)	rs3815849	1
POLR1A	C>C/G	2	86317012	het	81	missense_variant	158	R/P	tolerated(0.21)	benign(0.217)	rs146078741	1
USH2A	C>C/T	1	216051157	het	165	missense_variant	2875	R/Q	tolerated(0.21)	benign(0.267)	rs12118814	2
PRAME	A>A/T	22	22893394	het	53	missense_variant	47	L/M	tolerated(0.2)	possibly_damaging(0.721)	rs41310248	1
LRRC37A3	G>G/C	17	62856340	het	165	missense_variant	1308	H/Q	tolerated(0.2)	benign(0.084)	rs148835942	1
OR4C46	G>G/A	11	51516191	het	145	missense_variant	304	D/N	tolerated(0.2)	benign(0.002)	rs140000110	1
HMX3	C>C/G	10	124895700	het	34	missense_variant	45	P/R	tolerated(0.2)	benign(0.021)		0
DNAJC13	G>G/A	3	132218621	het	164	missense_variant	1462	R/H	tolerated(0.2)	benign(0.001)	rs61748103	1
ANKRD36	C>C/T	2	97833466	het	57	missense_variant	532	A/V	tolerated(0.2)	possibly_damaging(0.51)	rs112133169	0
IGFN1	G>G/A	1	201179068	het	40	missense_variant	1683	G/R	tolerated(0.2)	probably_damaging(0.98)	rs199816935	0
NPHS1	G>G/A	19	36337014	het	128	missense_variant	508	T/I	tolerated(0.19)	benign(0.008)		0
KCNJ12	C>C/T	17	21319285	het	389	missense_variant	211	L/F	tolerated(0.19)	possibly_damaging(0.66)	rs72846667	0
DNAH2	G>G/A	17	7669761	het	60	missense_variant	1213	E/K	tolerated(0.19)	benign(0.258)	rs35788701	1
MUC6	A>A/C	11	1017325	het	388	missense_variant	1826	Y/D	tolerated(0.19)	probably_damaging(0.943)	rs55903826	0
TOR4A	C>C/T	9	140174307	het	159	missense_variant	389	A/V	tolerated(0.19)	benign(0.126)	rs199638778	0
MUSK	T>T/C	9	113459618	het	183	missense_variant	167	I/T	tolerated(0.19)	benign(0.021)	rs202045225	0
BMP2K	G>G/C	4	79792163	het	69	missense_variant	486	Q/H	tolerated(0.19)	unknown(0)	rs2114202	2
NOP14-AS1,N	G>G/A	4	2944051	het	141	missense_variant	640	A/V	tolerated(0.19)	possibly_damaging(0.47)		0
CPO	A>A/G	2	207814400	het	126	missense_variant	43	E/G	tolerated(0.19)	benign(0.002)		0
LILRA4	C>C/T	19	54848121	het	79	missense_variant	416	V/M	tolerated(0.18)	benign(0.06)	rs141881690	0
SORCS1	T>T/C	10	108439488	het	92	missense_variant	522	Y/C	tolerated(0.18)	probably_damaging(0.949)		0
IPPK	G>G/A	9	95396712	het	183	missense_variant	376	L/F	tolerated(0.18)	probably_damaging(0.986)	rs2277170	3
GREB1	G>G/A	2	11758429	het	57	missense_variant	1143	G/D	tolerated(0.18)	benign(0.151)	rs145454387	2
NBPF14	G>G/A	1	148010911	het	2	missense_variant	571	R/C	tolerated(0.18)	unknown(0)	rs201061774	0
HNRNPCL1	C>C/T	1	12907803	het	158	missense_variant	114	G/S	tolerated(0.18)	benign(0.002)	rs141460546	0
RGAG1	G>G/C	X	109696568	het	126	missense_variant	908	G/A	tolerated(0.17)	benign(0.189)		0
KCNJ12	G>G/T	17	21319868	het	78	missense_variant	405	S/I	tolerated(0.17)	benign(0.029)	rs73979902	0
STARD9	A>A/G	15	42953372	het	232	missense_variant	240	N/S	tolerated(0.17)	benign(0)	rs148862329	0.18
LEMD2	A>A/G	6	33752201	het	21	missense_variant	261	C/R	tolerated(0.17)	benign(0.04)	rs140495067	0
IFT122	T>T/C	3	129214358	het	135	missense_variant	757	F/L	tolerated(0.17)	probably_damaging(0.925)	rs73204230	0
NBPF14,PDE4	A>A/T	1	144871755	het	28	missense_variant	1736	V/E	tolerated(0.17)	benign(0.378)	rs1778159	0
LSS	C>C/G	21	47648461	het	76	missense_variant	23	G/R	tolerated(0.16)	benign(0)		0
TRIM47	C>C/A	17	73874367	het	8	missense_variant	88	G/V	tolerated(0.16)	benign(0.029)		0
PITPNM1	C>C/T	11	67265774	het	82	missense_variant	502	D/N	tolerated(0.16)	possibly_damaging(0.598)		0
NCR3LG1	C>C/T	11	17393569	het	7	missense_variant	292	S/F	tolerated(0.16)	benign(0.008)		0
MUC6	A>A/C	11	1017789	het	520	missense_variant	1671	M/R	tolerated(0.16)	unknown(0)	rs78819924	0
SEC24C	A>A/G	10	75523634	het	115	missense_variant	374	I/V	tolerated(0.16)	benign(0.195)	rs35528438	1
ST8SIA6	T>T/A	10	17495619	het	85	missense_variant	47	T/S	tolerated(0.16)	benign(0.006)	rs45462798	2
PREX2	C>C/T	8	69020558	het	79	missense_variant	977	S/L	tolerated(0.16)	benign(0)	rs61753700	1
CYTH3	T>T/C	7	6226685	het	182	missense_variant	82	K/R	tolerated(0.16)	benign(0.008)	rs61753121	0
ZSCAN16	G>G/A	6	28097495	het	50	missense_variant	272	A/T	tolerated(0.16)	benign(0.079)	rs1383394	0.18
JAGN1	A>A/G	3	9934753	het	135	missense_variant	82	I/V	tolerated(0.16)	benign(0.048)	rs35365817	2
NBPF14,NOT	C>C/A	1	145281613	het	523	missense_variant	181	S/R	tolerated(0.16)	possibly_damaging(0.705)	rs12120756	0
CEP89	T>T/G	19	33444588	het	212	missense_variant	142	D/A	tolerated(0.15)	benign(0.041)	rs73035551	0
GPRIN2	G>G/A	10	46999596	het	232	missense_variant	239	R/K	tolerated(0.15)	benign(0.219)	rs7895979	0
PKD1L1	T>T/C	7	47880159	het	78	missense_variant	1818	I/V	tolerated(0.15)	possibly_damaging(0.714)		0
PRR18	G>G/T	6	166721108	het	38	missense_variant	175	L/I	tolerated(0.15)	possibly_damaging(0.596)	rs200995730	0
AKAP12	T>T/C	6	151670656	het	34	missense_variant	377	V/A	tolerated(0.15)	benign(0.005)	rs142810400	0.27
MRPS27	G>G/A	5	71521964	het	73	missense_variant	253	P/S	tolerated(0.15)	probably_damaging(0.999)	rs114360965	1
DNER	T>T/A	2	230450646	het	91	missense_variant	259	T/S	tolerated(0.15)	benign(0.085)	rs72987941	1
ADAM17	G>G/A	2	9630541	het	83	missense_variant	747	S/L	tolerated(0.15)	benign(0.005)	rs55796712	0.46
FAM47C	C>C/T	X	37027219	het	47	missense_variant	246	L/F	tolerated(0.14)	possibly_damaging(0.795)	rs148119466	0.18
TTC3	G>G/C	21	38563686	het	52	missense_variant	1692	L/F	tolerated(0.14)	benign(0.334)	rs61748840	1
SNRNP70	C>C/T	19	49611690	het	55	missense_variant	435	A/V	tolerated(0.14)	unknown(0)		0
AACS	G>G/A	12	125621219	het	28	missense_variant	564	E/K	tolerated(0.14)	benign(0.004)	rs77320648	0
PRB4	G>G/T	12	11461706	het	68	missense_variant	71	P/T	tolerated(0.14)	unknown(0)	rs12308381	0
MAML2	G>G/C	11	95825755	het	75	missense_variant	480	I/M	tolerated(0.14)	unknown(0)	rs61749251	1
C2orf71	C>C/T	2	29294253	het	73	missense_variant	959	A/T	tolerated(0.14)	benign(0.089)	rs192350796	0.05
SRMS	C>C/T	20	62173916	het	89	missense_variant	222	V/M	tolerated(0.13)	benign(0.015)	rs117606095	1
LILRA6	G>G/A	19	54744794	het	49	missense_variant	290	H/Y	tolerated(0.13)	benign(0.154)	rs76869766	0
KCNJ12	C>C/A	17	21319079	het	277	missense_variant	142	T/N	tolerated(0.13)	probably_damaging(0.959)	rs76518282	0
WDR16	C>C/T	17	9497560	het	78	missense_variant	153	A/V	tolerated(0.13)	possibly_damaging(0.859)	rs77839011	0.32
PATE1	C>C/G	11	125617691	het	93	missense_variant	74	A/G	tolerated(0.13)	benign(0.018)	rs12286611	0.32
MUC6	G>G/A	11	1017307	het	314	missense_variant	1832	P/S	tolerated(0.13)	possibly_damaging(0.62)		0
MUC6	G>G/T	11	1017183	het	334	missense_variant	1873	P/Q	tolerated(0.13)	probably_damaging(0.981)	rs34844844	0
MUC6	G>G/A	11	1017084	het	640	missense_variant	1906	P/L	tolerated(0.13)	benign(0.046)	rs34649796	0
TIMM23	C>C/T	10	51623147	het	51	missense_variant	23	G/E	tolerated(0.13)	unknown(0)		0
NPY4R	G>G/A	10	47087609	het	453	missense_variant	276	V/M	tolerated(0.13)	probably_damaging(0.906)	rs79871698	0
WDR34	C>C/T	9	131397116	het	54	missense_variant	356	G/S	tolerated(0.13)	possibly_damaging(0.665)	rs17849504	0.32
PABPC1	G>G/A	8	101718965	het	45	missense_variant	506	R/C	tolerated(0.13)	benign(0.012)	rs62513921	0
C8orf58	C>C/T	8	22458760	het	57	missense_variant	136	R/C	tolerated(0.13)	benign(0.005)	rs202142296	0
FAT1	G>G/C	4	187538194	het	352	missense_variant	3014	L/V	tolerated(0.13)	possibly_damaging(0.654)		0
SLC39A8	C>C/T	4	103265765	het	49	missense_variant	19	G/R	tolerated(0.13)	benign(0.229)		0
KALRN	C>C/G	3	124374484	het	141	missense_variant	1943	D/E	tolerated(0.13)	benign(0.302)		0
VCX3A	C>C/T	X	6451869	het	5	missense_variant	160	V/M	tolerated(0.12)	unknown(0)	rs35874450	0
CCDC155	G>G/A	19	49898404	het	26	missense_variant	64	V/M	tolerated(0.12)	probably_damaging(0.998)	rs191973091	1
ABHD15	C>C/T	17	27889643	het	78	missense_variant	448	R/Q	tolerated(0.12)	probably_damaging(0.992)	rs62070804	1
SDHD	A>A/G	11	111958677	het	147	missense_variant	50	H/R	tolerated(0.12)	possibly_damaging(0.797)	rs11214077	1
OR9G1	A>A/T	11	56468449	het	135	missense_variant	196	I/F	tolerated(0.12)	benign(0.004)	rs78119083	0
GPRIN2	A>A/C	10	46999178	het	102	missense_variant	100	T/P	tolerated(0.12)	benign(0.434)	rs7090312	0
PTPLA	C>C/G	10	17659149	het	50	missense_variant	64	E/Q	tolerated(0.12)	benign(0)	rs7895850	0
SNAPC4	T>T/G	9	139273128	het	34	missense_variant	1051	T/P	tolerated(0.12)	possibly_damaging(0.747)		0
POM121	C>C/T	7	72413423	het	5	missense_variant	699	P/L	tolerated(0.12)	probably_damaging(0.974)	rs71554686	0
PCDHGA1,PC	G>G/T	5	140730384	het	116	missense_variant	186	S/I	tolerated(0.12)	possibly_damaging(0.699)	rs62378414	2
CDH9	T>T/C	5	26885797	het	62	missense_variant	603	E/G	tolerated(0.12)	possibly_damaging(0.5)	rs34490509	1
IRS1	C>C/G	2	227661003	het	116	missense_variant	818	G/R	tolerated(0.12)	probably_damaging(0.998)	rs41265094	1
TPO	C>C/T	2	1440117	het	222	missense_variant	148	A/V	tolerated(0.12)	probably_damaging(0.905)	rs138509145	0
SUSD2	G>G/A	22	24579049	het	58	missense_variant	34	R/H	tolerated(0.11)	benign(0.188)	rs62231981	0
SLC12A5	G>G/A	20	44684856	het	106	missense_variant	975	R/H	tolerated(0.11)	benign(0.004)	rs142740233	0.09
ADRA1D	G>G/T	20	4229375	het	31	missense_variant	77	A/E	tolerated(0.11)	benign(0.002)	rs61759853	1
GRIK5	T>T/C	19	42557803	het	43	missense_variant	407	N/S	tolerated(0.11)	benign(0.056)		0
SF3B2	C>C/G	11	65827260	het	189	missense_variant	470	A/G	tolerated(0.11)	benign(0.11)		0
OR9G1	C>C/T	11	56468111	het	48	missense_variant	83	T/I	tolerated(0.11)	benign(0.047)	rs602224	0
OR4C3	G>G/C	11	48346916	het	223	missense_variant	142	V/L	tolerated(0.11)	benign(0.101)	rs77069283	0
MUC6	C>C/A	11	1017338	het	418	missense_variant	1821	Q/H	tolerated(0.11)	benign(0.064)	rs78943453	0
MUC6	G>G/A	11	1017240	het	205	missense_variant	1854	T/M	tolerated(0.11)	probably_damaging(0.988)	rs62891761	0
FRG2B	C>C/T	10	135438955	het	347	missense_variant	162	R/Q	tolerated(0.11)	benign(0.002)	rs201563622	0
C10orf32	C>C/T	10	104614123	het	73	missense_variant	27	T/I	tolerated(0.11)	benign(0.01)		0
MRPL50	C>C/T	9	104153052	het	153	missense_variant	58	R/Q	tolerated(0.11)	benign(0.234)	rs150907336	0.09
GDA	A>A/G	9	74817636	het	84	missense_variant	121	E/G	tolerated(0.11)	benign(0.085)		0
CCDC171	T>T/G	9	15874585	het	48	missense_variant	1175	L/R	tolerated(0.11)	possibly_damaging(0.87)	rs149814894	1
DPY19L1	T>T/C	7	34978952	het	47	missense_variant	561	S/G	tolerated(0.11)	probably_damaging(0.971)		0

CHCHD6	C>C/T	3	126571529	het	70	missense_variant	151	R/C	tolerated(0.11)	benign(0.032)	rs137859632	0
ANAPC1	C>C/G	2	112615888	het	129	missense_variant	451	Q/H	tolerated(0.11)	benign(0.007)	rs79100806	0
SCARF2	G>G/A	22	20780296	het	77	missense_variant	661	P/L	tolerated(0.1)	probably_damaging(0.998)	rs9680797	2
KRT31	G>G/A	17	39551763	het	158	missense_variant	234	A/V	tolerated(0.1)	benign(0.257)	rs112544857	2
CDH1	G>G/A	16	68863674	het	71	missense_variant	805	D/N	tolerated(0.1)	probably_damaging(1)	rs200894246	0
PTX4	C>C/T	16	1536466	het	129	missense_variant	299	R/H	tolerated(0.1)	benign(0.12)	rs61751878	0.14
PARP4	G>G/A	13	25021201	het	59	missense_variant	1080	L/F	tolerated(0.1)	benign(0.028)	rs201405094	0
DCHS1	G>G/T	11	6649927	het	128	missense_variant	1766	P/T	tolerated(0.1)	benign(0.301)		0
PCMTD1	G>G/A	8	52733228	het	24	missense_variant	253	R/C	tolerated(0.1)	possibly_damaging(0.852)	rs73592211	0
PCM1	G>G/A	8	17830005	het	61	missense_variant	1251	R/H	tolerated(0.1)	benign(0.063)	rs17514547	1
AEBP1	C>C/T	7	44152266	het	52	missense_variant	776	A/V	tolerated(0.1)	possibly_damaging(0.534)	rs144799697	0
KIAA2022	G>G/A	X	73960146	het	427	missense_variant	1416	P/S	tolerated(0.09)	benign(0.002)	rs143577015	0.12
ZXDB	G>G/A	X	57618845	het	12	missense_variant	122	E/K	tolerated(0.09)	benign(0.001)	rs199513692	0
PRR14L	C>C/T	22	32108475	het	83	missense_variant	1784	V/I	tolerated(0.09)	benign(0.001)	rs16989427	2
CHD6	G>G/C	20	40162062	het	102	missense_variant	61	L/V	tolerated(0.09)	benign(0.028)	rs61753666	1
CDH5	G>G/A	16	66434776	het	133	missense_variant	565	R/H	tolerated(0.09)	possibly_damaging(0.781)	rs140269853	0.27
SNX29	C>C/T	16	12162979	het	98	missense_variant	437	L/F	tolerated(0.09)	benign(0.001)		0
ZFYVE26	C>C/T	14	68219200	het	49	missense_variant	2411	R/H	tolerated(0.09)	possibly_damaging(0.74)	rs34373049	1
TBX3	C>C/T	12	115109681	het	54	missense_variant	733	A/T	tolerated(0.09)	benign(0.007)		0
KRT6C	A>A/G	12	52867401	het	9	missense_variant	41	S/P	tolerated(0.09)	unknown(0)		0
C1QTNF5,MF	C>C/A	11	119213319	het	91	missense_variant	458	L/F	tolerated(0.09)	benign(0.363)	rs145881139	0.41
MUC6	T>T/C	11	1017337	het	417	missense_variant	1822	T/A	tolerated(0.09)	benign(0.259)	rs76686156	0
PSD3	C>C/A	8	18730030	het	186	missense_variant	115	R/I	tolerated(0.09)	benign(0.08)	rs118013117	1
FRG1	G>G/A	4	190876242	het	197	missense_variant	123	G/E	tolerated(0.09)	benign(0.422)	rs1061653	0
NAF1	A>A/G	4	164069540	het	32	missense_variant	196	I/T	tolerated(0.09)	benign(0.349)		0
MUC20	G>G/A	3	195451841	het	188	missense_variant	123	G/R	tolerated(0.09)	probably_damaging(0.993)	rs201228126	0
OR2T5	G>G/A	1	248652023	het	228	missense_variant	45	G/E	tolerated(0.09)	benign(0.09)	rs139516854	0
HMCN1	C>C/T	1	186092103	het	19	missense_variant	4084	H/Y	tolerated(0.09)	possibly_damaging(0.658)	rs41317489	1
GFAP	C>C/G	17	42990750	het	82	missense_variant	223	E/Q	tolerated(0.08)	possibly_damaging(0.872)	rs56679084	0
KCNJ12	C>C/T	17	21319208	het	373	missense_variant	185	A/V	tolerated(0.08)	possibly_damaging(0.481)	rs73979896	0
ADAM21	T>T/G	14	70924602	het	58	missense_variant	129	F/C	tolerated(0.08)	benign(0.016)	rs72735759	0
TXNDC16	G>G/A	14	52955155	het	34	missense_variant	345	S/F	tolerated(0.08)	benign(0.005)	rs199524742	0
GAS2L3	G>G/A	12	101016071	het	43	missense_variant	223	D/N	tolerated(0.08)	possibly_damaging(0.658)	rs117065230	0.14
MUS81	G>G/A	11	65632507	het	86	missense_variant	431	R/H	tolerated(0.08)	probably_damaging(0.981)	rs61754785	1
FSD1L	G>G/T	9	108234242	het	18	missense_variant	133	A/S	tolerated(0.08)	probably_damaging(0.908)	rs117347201	0.32
SMAP1	C>C/A	6	71377781	het	67	missense_variant	19	L/I	tolerated(0.08)	benign(0.136)	rs112439957	0.37
ADAMTS2	C>C/T	5	178555097	het	145	missense_variant	827	R/Q	tolerated(0.08)	benign(0.102)	rs35445112	2
GEMIN5	G>G/C	5	154307070	het	187	missense_variant	319	R/G	tolerated(0.08)	possibly_damaging(0.8)	rs35522740	1
TACC3	G>G/A	4	1729910	het	136	missense_variant	261	G/R	tolerated(0.08)	benign(0.005)		0
OR5H1	A>A/G	3	97852347	het	29	missense_variant	269	D/G	tolerated(0.08)	benign(0.002)	rs111392904	0
C3orf14	A>A/G	3	62317005	het	111	missense_variant	61	I/M	tolerated(0.08)	benign(0.036)	rs35261777	1
RP2	C>C/T	X	46719498	het	231	missense_variant	282	R/W	tolerated(0.07)	benign(0.112)	rs1805147	1
RHBDD3	G>G/A	22	29656812	het	15	missense_variant	192	R/W	tolerated(0.07)	benign(0.411)	rs148811620	0.23
LRFN3	C>C/T	19	36435631	het	115	missense_variant	533	P/S	tolerated(0.07)	benign(0.158)	rs115982229	0.18
EVPL	G>G/T	17	74017554	het	62	missense_variant	336	R/S	tolerated(0.07)	benign(0.01)	rs74955334	0
OSGIN1	T>T/C	16	83999548	het	62	missense_variant	457	V/A	tolerated(0.07)	possibly_damaging(0.716)	rs62640905	1
NHLRC3	T>T/C	13	39621897	het	104	missense_variant	293	I/T	tolerated(0.07)	probably_damaging(0.948)	rs200432148	0.05
B4GALNT4	T>T/A	11	375764	het	97	missense_variant	326	F/I	tolerated(0.07)	benign(0.343)		0
CHAT	G>G/A	10	50854637	het	91	missense_variant	400	D/N	tolerated(0.07)	benign(0.224)	rs8178991	1
SVIL	T>T/C	10	29822358	het	82	missense_variant	313	E/G	tolerated(0.07)	probably_damaging(0.918)	rs138539716	0.14
KIAA0368	T>T/G	9	114202704	het	148	missense_variant	341	T/P	tolerated(0.07)	probably_damaging(0.934)		0
DPYS	C>C/T	8	105441890	het	88	missense_variant	278	G/D	tolerated(0.07)	probably_damaging(0.996)		0
RP1L1	C>C/T	8	10467503	het	317	missense_variant	1369	E/K	tolerated(0.07)	unknown(0)	rs116242305	1
VGF	C>C/T	7	100806639	het	7	missense_variant	496	A/T	tolerated(0.07)	benign(0.198)		0
ROBO2	G>G/A	3	75986717	het	178	missense_variant	25	V/M	tolerated(0.07)	benign(0.003)	rs78834776	0
OBSCN	A>A/C	1	228528268	het	39	missense_variant	5826	Q/P	tolerated(0.07)	probably_damaging(0.996)		0
TSPEAR,KRTA	C>C/G	21	46020742	het	6	missense_variant	74	P/R	tolerated(0.06)	benign(0.012)	rs62220888	0
CHD6	T>T/A	20	40049450	het	163	missense_variant	1942	H/L	tolerated(0.06)	benign(0.092)	rs140143834	0.37
ZNF814	G>G/A	19	58385748	het	14	missense_variant	337	A/V	tolerated(0.06)	unknown(0)	rs145250945	0
PPP1R15A	G>G/T	19	49377288	het	46	missense_variant	266	E/D	tolerated(0.06)	probably_damaging(0.946)		0
CSPG4	C>C/T	15	75982085	het	77	missense_variant	441	E/K	tolerated(0.06)	probably_damaging(0.938)	rs79463888	0
FREM2	C>C/T	13	39266472	het	124	missense_variant	1664	A/V	tolerated(0.06)	benign(0.392)	rs149779771	0.09
OR5A1	C>C/T	11	59210646	het	53	missense_variant	2	S/F	tolerated(0.06)	benign(0.001)	rs138097107	0
PEX16	C>C/T	11	45937306	het	168	missense_variant	103	V/M	tolerated(0.06)	benign(0.126)	rs11553094	2
LMO2	C>C/G	11	33891012	het	12	missense_variant	43	G/A	tolerated(0.06)	unknown(0)		0
TPH1	T>T/A	11	18045484	het	109	missense_variant	326	Q/L	tolerated(0.06)	benign(0.314)		0
MUC6	G>G/A	11	1016640	het	199	missense_variant	2054	A/V	tolerated(0.06)	benign(0.33)	rs111704427	0
ADAM8	G>G/A	10	135086766	het	14	missense_variant	189	R/W	tolerated(0.06)	possibly_damaging(0.705)	rs45451297	2
MYPN	C>C/T	10	69959174	het	76	missense_variant	1112	P/L	tolerated(0.06)	probably_damaging(0.988)	rs71534278	0.23
C9orf96	C>C/T	9	136266918	het	121	missense_variant	417	S/F	tolerated(0.06)	possibly_damaging(0.572)		0
DOCK8	G>G/A	9	312030	het	79	missense_variant	202	R/H	tolerated(0.06)	probably_damaging(0.98)		0
RSPH10B2	C>C/T	7	6803602	het	66	missense_variant	148	T/M	tolerated(0.06)	probably_damaging(0.996)	rs200943820	0
RANBP17	G>G/A	5	170640676	het	241	missense_variant	758	R/Q	tolerated(0.06)	benign(0)	rs149025321	0
PCDHB5	C>C/G	5	140517034	het	19	missense_variant	673	P/R	tolerated(0.06)	probably_damaging(0.977)	rs139801121	2
ESPNL	C>C/T	2	239033974	het	40	missense_variant	351	R/C	tolerated(0.06)	possibly_damaging(0.542)	rs73100390	1
POTEJ	A>A/C	2	131414878	het	1	missense_variant	849	T/P	tolerated(0.06)	possibly_damaging(0.587)	rs201354416	0
CLHC1	C>C/A	2	55449412	het	107	missense_variant	46	A/S	tolerated(0.06)	possibly_damaging(0.835)	rs74785222	2
CEP170	G>G/A	1	243329049	het	295	missense_variant	738	T/I	tolerated(0.06)	benign(0.018)	rs3766664	0
ARID4B	C>C/T	1	235331875	het	117	missense_variant	1302	A/T	tolerated(0.06)	benign(0.152)		0
NBPF14,NOT	A>A/T	1	145281656	het	387	missense_variant	196	T/S	tolerated(0.06)	probably_damaging(0.984)	rs4649852	0
PRAMEF1	C>C/G	1	12854479	het	190	missense_variant	235	R/G	tolerated(0.06)	benign(0.001)	rs1063775	0
CHD5	G>G/A	1	6215713	het	142	missense_variant	151	S/L	tolerated(0.06)	benign(0.411)	rs150062924	0.14
CDC27	T>T/C	17	45214605	het	46	missense_variant	615	H/R	tolerated(0.05)	possibly_damaging(0.763)	rs76926116	0
DIS3L	G>G/A	15	66618297	het	202	missense_variant	599	G/E	tolerated(0.05)	probably_damaging(0.908)		0
KL	C>C/T	13	33635161	het	147	missense_variant	649	R/C	tolerated(0.05)	benign(0.128)	rs199843173	0.05
TBX3	T>T/C	12	115110001	het	122	missense_variant	626	H/R	tolerated(0.05)	possibly_damaging(0.83)		0
KLRF1	G>G/A	12	9984965	het	40	missense_variant	47	V/M	tolerated(0.05)	benign(0.005)	rs146460124	0.27
ANGPTL5	G>G/A	11	101765654	het	58	missense_variant	268	T/M	tolerated(0.05)	possibly_damaging(0.772)	rs77315074	2
OR9G1	T>T/C	11	56468704	het	194	missense_variant	281	F/L	tolerated(0.05)	benign(0.072)	rs79288825	0
MUC6	G>G/A	11	1017963	het	151	missense_variant	1613	P/L	tolerated(0.05)	unknown(0)	rs61869008	0
TTC40	G>G/A	10	134682781	het	20	missense_variant	1536	A/V	tolerated(0.05)	unknown(0)	rs139662727	0.23
AGAP9	C>C/T	10	48235892	het	6	missense_variant	220	P/L	tolerated(0.05)	benign(0.001)	rs200283865	0
BCLAF1	G>G/A	6	136582417	het	28	missense_variant	915	R/C	tolerated(0.05)	unknown(0)	rs62431283	0
FAM153B	C>C/T	5	175533585	het	21	missense_variant	208	P/L	tolerated(0.05)	benign(0.02)	rs199655223	0
PCDHGA1,PC	A>A/G	5	140731022	het	185	missense_variant	399	K/E	tolerated(0.05)	possibly_damaging(0.753)	rs77250251	2
ZNF141	G>G/A	4	367169	het	84	missense_variant	315	E/K	tolerated(0.05)	benign(0.098)	rs145966198	0
NEB	C>C/T	2	152499143	het	103	missense_variant	2773	R/Q	tolerated(0.05)	probably_damaging(0.991)	rs35974308	2
TEKT4	C>C/T	2	95537568	het	88	missense_variant	82	R/C	tolerated(0.05)	benign(0.101)	rs80243548	1
IGFN1	A>A/G	1	201178965	het	75	missense_variant	1648	I/M	tolerated(0.05)	benign(0.046)	rs12758143	0
NBPF14,NOT	C>C/T	1	145281543	het	842	missense_variant	158	T/I	tolerated(0.05)	possibly_damaging(0.815)	rs8002	0
CLCNKA	G>G/A	1	16349137	het	26	missense_variant	8	R/H	tolerated(0.05)	benign(0.038)	rs9442189	1
MYO18A	G>G/A	17	27437032	het	126	missense_variant	1059	R/C	deleterious(0.05)	probably_damaging(0.937)	rs200969774	0
C17orf74	C>C/T	17	7330328	het	90	missense_variant	340	R/W	deleterious(0.05)	probably_damaging(0.958)		0
ATP2A3	C>C/G	17	3844394	het	158	missense_variant	657	E/D	deleterious(0.05)	benign(0.075)		0
CNTNAP4	A>A/G	16	76482001	het	88	missense_variant	186	K/E	deleterious(0.05)	probably_damaging(0.962)	rs35363016	1
USP31	C>C/T	16	23096952	het	56	missense_variant	552	A/T	deleterious(0.05)	probably_damaging(0.968)	rs9932912	1
GAS2L3	G>G/A	12	101016068	het	43	missense_variant	222	E/K	deleterious(0.05)	possibly_damaging(0.827)	rs117541078	0.14
KDM4E	A>A/G	11	94758981	het	87	missense_variant	87	H/R	deleterious(0.05)	benign(0.016)	rs62617142	1
MUC6	G>G/A	11	1018419	het	82	missense_variant	1461	T/I	deleterious(0.05)	unknown(0)	rs148581741	0
ZC3H3	T>T/G	8	144547913	het	66	missense_variant	761	K/Q	deleterious(0.05)	probably_damaging(0.964)		0
CDKL2	A>A/G	4	76551117	het	83	missense_variant	19	M/T	deleterious(0.05)	benign(0.401)		0
DNPEP	T>T/G	2	220251685	het	72	missense_variant	50	N/H	deleterious(0.05)	benign(0.037)	rs11539909	2
ZDBF2	G>G/T	2	207175476	het	120	missense_variant	2075	R/M	deleterious(0.05)	possibly_damaging(0.737)	rs36095066	0.46
SLC3A1	A>A/T	2	44531310	het	113	missense_variant	389	S/C	deleterious(0.05)	possibly_damaging(0.795)		0
OBSCN	C>C/T	1	228538635	het	86	missense_variant	6137	P/L	deleterious(0.05)	possibly_damaging(0.883)	rs140996408	0.18
NBPF14,NBPI	C>C/A	1	145293425	het	16	missense_variant	7	P/H	deleterious(0.05)	probably_damaging(0.968)	rs7366490	0
CDC42EP1	G>G/A	22	37962646	het	73	missense_variant	97	R/Q	deleterious(0.04)	unknown(0)	rs139353065	1
C22orf15	G>G/A	22	24106506	het	82	missense_variant	58	E/K	deleterious(0.04)	probably_damaging(0.985)	rs143257161	0.18
IGLL1	C>C/T	22	23915620	het	122	missense_variant	159	G/S	deleterious(0.04)	possibly_damaging(0.869)	rs139571703	4
KIR3DL1	G>G/A	19	55331384	het	202	missense_variant	191	G/E	deleterious(0.04)	probably_damaging(1)		0
KLK13	G>G/A	19	51563265	het	160	missense_variant	109	H/Y	deleterious(0.04)	probably_damaging(0.959)	rs34089525	1
CILP2	A>A/G	19	19656556	het	104	missense_variant	1068	S/G	deleterious(0.04)	benign(0.006)	rs147526697	0.09
CTAGE1	T>T/C	18	19996805	het	5	missense_variant	324	I/V	deleterious(0.04)	benign(0.054)	rs12961009	2
ITGA2B	C>C/T	17	42455780	het	40	missense_variant	682	V/M	deleterious(0.04)	probably_damaging(0.981)	rs13306473	0
KCNJ12	G>G/A	17	21319786	het	154	missense_variant	378	E/K	deleterious(0.04)	probably_damaging(0.932)	rs78547883	0
KCNJ12	C>C/T	17	21319682	het	251	missense_variant	343	S/L	deleterious(0.04)	benign(0.101)	rs80203231	0
TROAP	C>C/T	12	49721015	het	149	missense_variant	265	R/C	deleterious(0.04)	benign(0.306)	rs78961476	2
ZC3H12C	A>A/C	11	110036365	het	136	missense_variant	852	D/A	deleterious(0.04)	benign(0.269)	rs61756697	1
OR9G1	A>A/G	11	56468198	het	111	missense_variant	112	Y/C	deleterious(0.04)	probably_damaging(1)	rs4990194	0
PHF21A	G>G/T	11	45955558	het	48	missense_variant	668	S/R	deleterious(0.04)	probably_damaging(0.971)		0
ZDHHC13	A>A/G	11	19184916	het	31	missense_variant	392	Y/C	deleterious(0.04)	probably_damaging(0.999)	rs12798330	1
PPRC1	C>C/G	10	103900766	het	210	missense_variant	834	P/R	deleterious(0.04)	possibly_damaging(0.882)	rs17855877	0.09
UNC5B	A>A/G	10	73051273	het	119	missense_variant	460	Q/R	deleterious(0.04)	benign(0.272)	rs201117786	0
HKDC1	A>A/G	10	70987060	het	208	missense_variant	54	D/G	deleterious(0.04)	possibly_damaging(0.803)	rs10823320	2
PBX3	C>C/T	9	128678097	het	53	missense_variant	136	A/V	deleterious(0.04)	possibly_damaging(0.707)	rs145687528	0.32
GPR63	C>C/T	6	97247547	het	199	missense_variant	21	V/M	deleterious(0.04)	benign(0.079)	rs35358396	2
DCDC2	G>G/C	6	24302046	het	154	missense_variant	152	P/A	deleterious(0.04)	possibly_damaging(0.836)	rs33914824	3
DMXL1	A>A/G	5	118500264	het	120	missense_variant	1589	M/V	deleterious(0.04)	benign(0.019)	rs116379331	1

SENP5	A>A/C	3	196613072	het	119	missense_variant	340	L/F	deleterious(0.04)	benign(0.292)	rs34251880	1
MAP4	T>T/C	3	47913443	het	154	missense_variant	824	R/G	deleterious(0.04)	possibly_damaging(0.725)		0
DNAH6	A>A/G	2	84897501	het	33	missense_variant	2119	Y/C	deleterious(0.04)	probably_damaging(0.99)	rs17025409	2
HNRNPCL1	A>A/C	1	12907798	het	149	missense_variant	115	F/L	deleterious(0.04)	benign(0.006)	rs150096254	0
SLC13A3	C>C/G	20	45221055	het	69	missense_variant	303	G/A	deleterious(0.03)	possibly_damaging(0.661)	rs141554970	1
ZNF665	G>G/A	19	53668650	het	73	missense_variant	365	R/W	deleterious(0.03)	probably_damaging(0.972)		0
ZNF506	T>T/A	19	19905661	het	57	missense_variant	345	E/D	deleterious(0.03)	benign(0.402)	rs146885601	0.37
DNAH3	C>C/T	16	20963832	het	52	missense_variant	3704	R/K	deleterious(0.03)	benign(0.063)	rs147732992	1
ATP4B	C>C/T	13	114309237	het	158	missense_variant	45	V/M	deleterious(0.03)	probably_damaging(0.92)	rs142885298	0.05
SLC2A3	T>T/C	12	8074055	het	200	missense_variant	482	E/G	deleterious(0.03)	benign(0.245)	rs199523896	0
AKAP3	AGA>AGA/CAG	12	4735968	het	88	missense_variant	700	S/L	deleterious(0.03)	benign(0.298)		0
OR4C3	G>G/A	11	48346932	het	190	missense_variant	147	R/H	deleterious(0.03)	benign(0.069)	rs80285195	0
MUC6	G>G/A	11	1016910	het	573	missense_variant	1964	T/I	deleterious(0.03)	probably_damaging(0.992)	rs113559934	0
AIF1L	A>A/G	9	133995629	het	57	missense_variant	151	M/V	deleterious(0.03)	benign(0.021)	rs112844133	1
CDK5RAP2	T>T/C	9	123216045	het	165	missense_variant	828	K/E	deleterious(0.03)	benign(0.312)		0
KBTBD11	G>G/T	8	1951027	het	32	missense_variant	557	A/S	deleterious(0.03)	possibly_damaging(0.735)		0
KMT2C	G>G/A	7	151970931	het	228	missense_variant	291	L/F	deleterious(0.03)	probably_damaging(0.999)	rs56850341	0
BCLAF1	G>G/T	6	136582497	het	168	missense_variant	888	T/N	deleterious(0.03)	possibly_damaging(0.705)	rs62431284	0
MDN1	T>T/C	6	90371215	het	75	missense_variant	4883	D/G	deleterious(0.03)	benign(0.043)	rs114646660	1
RREB1	G>G/A	6	7248990	het	58	missense_variant	1673	R/Q	deleterious(0.03)	probably_damaging(0.996)	rs116683035	1
AGGF1	C>C/T	5	76355564	het	163	missense_variant	614	H/Y	deleterious(0.03)	benign(0.368)		0
ERCC8	T>T/C	5	60224715	het	16	missense_variant	50	D/G	deleterious(0.03)	probably_damaging(0.998)		0
OR5K4	A>A/T	3	98073065	het	104	missense_variant	123	Y/F	deleterious(0.03)	benign(0.13)	rs140109788	0.05
OR5H1	G>G/A	3	97852352	het	31	missense_variant	271	D/N	deleterious(0.03)	benign(0.127)		0
ZCWPW2	A>A/G	3	28454627	het	68	missense_variant	23	N/S	deleterious(0.03)	benign(0.005)	rs148504648	0.09
MPHOSPH10	G>G/A	2	71376471	het	33	missense_variant	595	R/Q	deleterious(0.03)	probably_damaging(0.98)	rs115361918	0.14
PPIAL4G	C>C/T	1	143767628	het	17	missense_variant	74	G/D	deleterious(0.03)	possibly_damaging(0.868)	rs6604514	0
WDR4	C>C/A	21	44299536	het	41	missense_variant	24	A/S	deleterious(0.02)	benign(0.388)	rs150449975	0.05
ADAMTS5	C>C/T	21	28305324	het	106	missense_variant	577	G/S	deleterious(0.02)	probably_damaging(0.999)	rs146717299	1
SIGLEC12	T>T/A	19	52000151	het	39	missense_variant	528	R/W	deleterious(0.02)	probably_damaging(0.951)	rs61743147	2
GRAMD1A	G>G/A	19	35512685	het	92	missense_variant	557	R/Q	deleterious(0.02)	possibly_damaging(0.643)	rs200473202	0.09
RHPN2	T>T/C	19	33490566	het	203	missense_variant	384	Q/R	deleterious(0.02)	probably_damaging(0.981)	rs201801079	0
MPND	G>G/T	19	4343738	het	8	missense_variant	14	G/V	deleterious(0.02)	unknown(0)		0
MGAT5B	C>C/T	17	74868887	het	162	missense_variant	19	T/M	deleterious(0.02)	unknown(0)	rs191831149	0.23
DCAKD	G>G/A	17	43111558	het	98	missense_variant	105	R/W	deleterious(0.02)	possibly_damaging(0.885)	rs117298907	2
KCNJ12	G>G/C	17	21319230	het	370	missense_variant	192	Q/H	deleterious(0.02)	benign(0.01)	rs1657742	0
DBNDD1	C>C/A	16	90075750	het	85	missense_variant	60	E/D	deleterious(0.02)	probably_damaging(0.952)	rs199893398	0.09
ZNF469	G>G/A	16	88495913	het	43	missense_variant	679	E/K	deleterious(0.02)	possibly_damaging(0.641)		0
PDPR	T>T/G	16	70182390	het	167	missense_variant	662	N/K	deleterious(0.02)	benign(0.066)	rs62050978	0
NDE1	C>C/T	16	15771722	het	94	missense_variant	101	A/V	deleterious(0.02)	benign(0.225)	rs201587506	0
ANKRD10	T>T/C	13	111532073	het	95	missense_variant	392	S/G	deleterious(0.02)	probably_damaging(0.984)		0
MMAB	G>G/C	12	109999620	het	94	missense_variant	129	A/G	deleterious(0.02)	probably_damaging(0.923)	rs201189970	0
H1FNT	C>C/T	12	48723142	het	72	missense_variant	23	A/V	deleterious(0.02)	possibly_damaging(0.705)	rs117292373	1
OR9G1	G>G/A	11	56468021	het	112	missense_variant	53	C/Y	deleterious(0.02)	benign(0)	rs532635	0
OR4C3	A>A/G	11	48347124	het	219	missense_variant	211	E/G	deleterious(0.02)	benign(0.002)	rs79019124	0
LAMC3	C>C/T	9	133901752	het	155	missense_variant	152	R/C	deleterious(0.02)	benign(0.065)	rs45628035	1
CDK5RAP2	C>C/T	9	123171544	het	57	missense_variant	1489	V/M	deleterious(0.02)	possibly_damaging(0.608)		0
FKTN	A>A/G	9	108397495	het	124	missense_variant	446	N/D	deleterious(0.02)	probably_damaging(0.991)	rs41313301	0.32
IFNA14	T>T/C	9	21239468	het	66	missense_variant	156	E/G	deleterious(0.02)	benign(0.216)		0
DLGAP2	A>A/T	8	1616572	het	48	missense_variant	550	T/S	deleterious(0.02)	benign(0.414)		0
PDIA4	G>G/A	7	148712092	het	79	missense_variant	173	T/M	deleterious(0.02)	benign(0.096)	rs2290971	2
ICA1	G>G/A	7	8258042	het	82	missense_variant	158	R/C	deleterious(0.02)	probably_damaging(0.996)		0
MDN1	C>C/T	6	90382002	het	135	missense_variant	4571	V/M	deleterious(0.02)	probably_damaging(0.937)	rs138617275	0
FRG1	C>C/G	4	190878654	het	638	missense_variant	178	I/M	deleterious(0.02)	possibly_damaging(0.888)	rs78653319	0
PROS1	C>C/T	3	93595837	het	90	missense_variant	615	V/M	deleterious(0.02)	benign(0.264)		0
TRABD2A	C>C/T	2	85059227	het	85	missense_variant	296	R/H	deleterious(0.02)	benign(0.018)	rs61744273	3
EXO1	A>A/G	1	242023898	het	130	missense_variant	279	N/S	deleterious(0.02)	possibly_damaging(0.867)	rs4149909	2
PPIAL4G	G>G/A	1	143767547	het	55	missense_variant	101	A/V	deleterious(0.02)	possibly_damaging(0.62)	rs2490183	0
PRAMEF12	C>C/A	1	12836101	het	193	missense_variant	235	L/I	deleterious(0.02)	probably_damaging(0.998)	rs41303871	0.41
SPIN2A	C>C/T	X	57162805	het	9	missense_variant	76	V/M	deleterious(0.01)	probably_damaging(0.977)		0
SUN2	C>C/T	22	39138517	het	92	missense_variant	307	R/H	deleterious(0.01)	probably_damaging(0.987)		0
SEC14L4	C>C/G	22	30888492	het	84	missense_variant	211	E/D	deleterious(0.01)	possibly_damaging(0.642)	rs61744139	1
DSCAM	C>C/T	21	41457598	het	111	missense_variant	1355	G/S	deleterious(0.01)	probably_damaging(0.999)	rs200670025	0
NFATC1	G>G/A	18	77170979	het	191	missense_variant	222	R/Q	deleterious(0.01)	probably_damaging(0.953)		0
CDC27	A>A/T	17	45214604	het	46	missense_variant	615	H/Q	deleterious(0.01)	probably_damaging(0.995)	rs75661039	0
KCNJ12	G>G/A	17	21319087	het	298	missense_variant	145	G/S	deleterious(0.01)	probably_damaging(1)	rs75029097	0
KCNJ12	G>G/A	17	21319069	het	279	missense_variant	139	E/K	deleterious(0.01)	probably_damaging(1)	rs76265595	0
DNAH2	C>C/G	17	7736242	het	104	missense_variant	4325	P/R	deleterious(0.01)	probably_damaging(0.995)	rs116930996	1
OR1D5	G>G/A	17	2966273	het	304	missense_variant	210	P/L	deleterious(0.01)	probably_damaging(0.979)	rs2676567	0
PKD1L2	C>C/G	16	81219131	het	27	missense_variant	655	V/L	deleterious(0.01)	probably_damaging(0.929)	rs201060208	0
DCTPP1	C>C/G	16	30435601	het	147	missense_variant	156	G/R	deleterious(0.01)	benign(0.107)	rs146094440	1
GTF3C1	C>C/T	16	27480797	het	246	missense_variant	1630	R/H	deleterious(0.01)	probably_damaging(0.975)	rs61739285	2
DUOXA1	G>G/A	15	45412435	het	139	missense_variant	213	T/M	deleterious(0.01)	probably_damaging(0.996)	rs149960164	0.32
SYNE2	G>G/A	14	64633984	het	99	missense_variant	5547	D/N	deleterious(0.01)	possibly_damaging(0.564)	rs17179194	1
AKAP11	G>G/C	13	42875097	het	309	missense_variant	739	A/P	deleterious(0.01)	benign(0.176)		0
PARP4	T>T/C	13	25021263	het	67	missense_variant	1059	Q/R	deleterious(0.01)	probably_damaging(0.991)	rs77269056	0
TBX3	T>T/C	12	115120915	het	61	missense_variant	31	S/G	deleterious(0.01)	probably_damaging(0.932)		0
ESPL1	T>T/G	12	53680011	het	124	missense_variant	1164	V/G	deleterious(0.01)	benign(0.039)	rs111320105	1
CLEC6A	A>A/C	12	8608730	het	35	missense_variant	8	Q/P	deleterious(0.01)	benign(0.412)	rs118084065	1
VWA5A	C>C/T	11	124016058	het	148	missense_variant	757	R/C	deleterious(0.01)	benign(0.043)	rs117689747	1
KRTAP5-9	C>C/T	11	71259816	het	40	missense_variant	38	P/L	deleterious(0.01)	unknown(0)	rs12791610	0
OR9G1	T>T/A	11	56468699	het	198	missense_variant	279	V/E	deleterious(0.01)	probably_damaging(0.988)	rs79251113	0
OR4C3	C>C/G	11	48347130	het	222	missense_variant	213	A/G	deleterious(0.01)	benign(0.018)	rs77395846	0
USP47	G>G/A	11	11971472	het	107	missense_variant	1061	G/R	deleterious(0.01)	probably_damaging(0.999)		0
OR52K2	C>C/T	11	4471026	het	238	missense_variant	153	R/W	deleterious(0.01)	benign(0.135)	rs143862015	0
CYP2C8	C>C/T	10	96797033	het	20	missense_variant	442	R/H	deleterious(0.01)	possibly_damaging(0.879)	rs138495387	0
SVIL	A>A/G	10	29783908	het	115	missense_variant	1259	M/T	deleterious(0.01)	possibly_damaging(0.752)	rs78773460	0
COL5A1	C>C/T	9	137708884	het	64	missense_variant	1379	P/S	deleterious(0.01)	unknown(0)	rs61739195	0.41
AMBP	T>T/C	9	116832007	het	45	missense_variant	192	E/G	deleterious(0.01)	possibly_damaging(0.472)	rs141738059	1
FGL1	C>C/A	8	17726069	het	147	missense_variant	256	W/L	deleterious(0.01)	probably_damaging(0.999)	rs2653414	1
FAM86B1	G>G/A	8	12042924	het	57	missense_variant	251	R/C	deleterious(0.01)	possibly_damaging(0.705)	rs200255336	0
KMT2C	C>C/T	7	151945007	het	227	missense_variant	838	G/S	deleterious(0.01)	probably_damaging(0.997)	rs137949498	0
BCLAF1	C>C/T	6	136590698	het	156	missense_variant	699	R/H	deleterious(0.01)	benign(0.408)	rs62431287	0
THEMIS	A>A/G	6	128176318	het	34	missense_variant	36	M/T	deleterious(0.01)	possibly_damaging(0.479)	rs148069630	0.14
GSTA3	A>A/C	6	52762730	het	173	missense_variant	147	Y/D	deleterious(0.01)	probably_damaging(0.962)	rs144126679	0
POM121L2	G>G/T	6	27279805	het	203	missense_variant	49	P/T	deleterious(0.01)	possibly_damaging(0.866)	rs61736098	2
POM121L2	G>G/T	6	27278179	het	162	missense_variant	591	P/T	deleterious(0.01)	possibly_damaging(0.811)	rs41269261	2
SPINK6	A>A/C	5	147593565	het	136	missense_variant	58	K/N	deleterious(0.01)	probably_damaging(0.992)		0
BRD8	G>G/A	5	137504975	het	76	missense_variant	193	S/F	deleterious(0.01)	probably_damaging(0.987)	rs139423469	0
TGFBI	C>C/T	5	135385161	het	152	missense_variant	269	L/F	deleterious(0.01)	probably_damaging(1)	rs199852470	0
VCAN	G>G/T	5	82786034	het	242	missense_variant	63	R/L	deleterious(0.01)	probably_damaging(0.989)	rs142148754	0
MSH3	G>G/T	5	80109547	het	163	missense_variant	934	G/C	deleterious(0.01)	probably_damaging(0.979)		0
CYP4V2	G>G/A	4	187130349	het	228	missense_variant	443	R/Q	deleterious(0.01)	probably_damaging(0.998)	rs72646291	0.18
FAT4	C>C/T	4	126373018	het	179	missense_variant	3616	T/M	deleterious(0.01)	possibly_damaging(0.817)	rs111423173	0.27
PPM1K	C>C/T	4	89199348	het	97	missense_variant	130	G/S	deleterious(0.01)	possibly_damaging(0.836)		0
SORCS2	C>C/A	4	7666180	het	72	missense_variant	351	D/E	deleterious(0.01)	benign(0.022)	rs35935435	1
PAK2	G>G/C	3	196529902	het	63	missense_variant	101	Q/H	deleterious(0.01)	possibly_damaging(0.887)	rs201465227	0
TOPBP1	G>G/A	3	133320203	het	14	missense_variant	1487	P/L	deleterious(0.01)	benign(0.252)		0
KALRN	C>C/A	3	124103800	het	297	missense_variant	625	R/S	deleterious(0.01)	benign(0.399)		0
ZNF717	C>C/T	3	75790860	het	5	missense_variant	29	V/M	deleterious(0.01)	probably_damaging(0.988)	rs150203551, rs	0
CDCP1	C>C/T	3	45127531	het	87	missense_variant	704	V/M	deleterious(0.01)	probably_damaging(0.999)		0
ANKRD36	G>G/A	2	97868100	het	56	missense_variant	977	E/K	deleterious(0.01)	possibly_damaging(0.853)		0
PROKR1	A>A/T	2	68882588	het	152	missense_variant	354	K/N	deleterious(0.01)	probably_damaging(0.949)		0
ITSN2	C>C/T	2	24440869	het	148	missense_variant	1238	R/H	deleterious(0.01)	unknown(0)		0
OR2T27	A>A/T	1	248813785	het	31	missense_variant	134	V/D	deleterious(0.01)	possibly_damaging(0.76)	rs200036975	0
KIF26B	C>C/G	1	245851609	het	111	missense_variant	1775	P/R	deleterious(0.01)	unknown(0)	rs150834033	2
IGFN1	G>G/A	1	201178819	het	148	missense_variant	1600	A/T	deleterious(0.01)	possibly_damaging(0.713)	rs72468019	0
RPF1	C>C/T	1	84963115	het	59	missense_variant	338	R/W	deleterious(0.01)	possibly_damaging(0.864)		0
SCMH1	C>C/T	1	41608646	het	146	missense_variant	96	D/N	deleterious(0.01)	probably_damaging(0.945)		0
MAGEC1	T>T/A	X	140993859	het	76	missense_variant	223	S/R	deleterious(0)	benign(0.182)	rs6634333	0
MST4	C>C/T	X	131188749	het	115	missense_variant	45	R/C	deleterious(0)	benign(0.14)	rs56044451	0.3
PRR5-ARHGA	C>C/T	22	45198034	het	130	missense_variant	53	R/W	deleterious(0)	probably_damaging(0.986)	rs41278883	1
SEC14L6	G>G/C	22	30927957	het	99	missense_variant	151	A/G	deleterious(0)	benign(0.23)	rs61742115	1
SUSD2	G>G/T	22	24579157	het	110	missense_variant	70	G/V	deleterious(0)	probably_damaging(0.999)	rs200945728	0
GNB1L,C22or	C>C/A	22	19838961	het	80	missense_variant	275	S/I	deleterious(0)	possibly_damaging(0.87)		0
RSPH1	A>A/C	21	43897478	het	122	missense_variant	217	L/W	deleterious(0)	probably_damaging(0.999)	rs138007679	1
TPTE	A>A/G	21	10941966	het	138	missense_variant	246	I/T	deleterious(0)	possibly_damaging(0.876)	rs149843545	0
CSE1L	T>T/C	20	47700600	het	100	missense_variant	507	L/S	deleterious(0)	probably_damaging(0.955)		0
BPIFB4	T>T/G	20	31671575	het	43	missense_variant	191	L/R	deleterious(0)	probably_damaging(0.982)	rs144272671	0
CSRP2BP	C>C/G	20	18162481	het	80	missense_variant	600	P/R	deleterious(0)	probably_damaging(0.997)	rs11557577	2
ZNF528	A>A/G	19	52919490	het	28	missense_variant	462	E/G	deleterious(0)	probably_damaging(0.949)		0
HSD17B14	G>G/A	19	49339590	het	142	missense_variant	27	R/C	deleterious(0)	probably_damaging(0.966)	rs141119542	0.14
PHLDB3	G>G/A	19	43982213	het	39	missense_variant	592	R/C	deleterious(0)	probably_damaging(0.999)		0
FCGBP	C>C/T	19	40392496	het	28	missense_variant	2670	G/R	deleterious(0)	probably_damaging(1)	rs143680639	0
PLEKHG2	G>G/C	19	39914225	het	47	missense_variant	844	R/P	deleterious(0)	probably_damaging(0.993)	rs201914405	0
PAK4	C>C/G	19	39669106	het	37	missense_variant	555	R/G	deleterious(0)	probably_damaging(0.995)	rs56084716	0.23
GGN	G>G/C	19	38875072	het	85	missense_variant	647	Q/E	deleterious(0)	probably_damaging(0.967)	rs62123481	0
ZNF30	G>G/T	19	35434582	het	97	missense_variant	239	G/W	deleterious(0)	possibly_damaging(0.763)	rs142299823	1

GTPBP3	C>C/T	19	17450287	het	124	missense_variant	317	R/C	deleterious(0)	probably_damaging(0.999)		0
NFATC1	G>G/A	18	77170903	het	229	missense_variant	197	V/M	deleterious(0)	probably_damaging(0.999)	rs62096875	0
PGS1	G>G/T	17	76399861	het	271	missense_variant	365	D/Y	deleterious(0)	probably_damaging(0.999)		0
GNA13	C>C/G	17	63010848	het	217	missense_variant	221	V/L	deleterious(0)	benign(0.184)	rs1062597	0.05
FMNL1	G>G/A	17	43322628	het	331	missense_variant	913	D/N	deleterious(0)	probably_damaging(1)	rs141887433	0.05
TMEM132E	C>C/T	17	32961937	het	130	missense_variant	513	T/M	deleterious(0)	possibly_damaging(0.892)		0
KCNJ12	A>A/G	17	21319943	het	33	missense_variant	430	E/G	deleterious(0)	probably_damaging(0.999)	rs5021699	0
CNTROB	C>C/T	17	7852724	het	68	missense_variant	892	R/C	deleterious(0)	probably_damaging(0.996)	rs151174639	0
GP1BA	T>T/C	17	4835991	het	211	missense_variant	31	V/A	deleterious(0)	possibly_damaging(0.502)	rs201827537	0
HSD17B2	T>T/C	16	82104685	het	110	missense_variant	206	L/P	deleterious(0)	probably_damaging(0.989)		0
PDPR	C>C/T	16	70162749	het	104	missense_variant	142	R/C	deleterious(0)	probably_damaging(1)	rs199978223	0
ZNF747	C>C/A	16	30545994	het	76	missense_variant	3	D/Y	deleterious(0)	probably_damaging(0.951)		0
PSTPIP1	A>A/G	15	77329417	het	99	missense_variant	384	D/G	deleterious(0)	possibly_damaging(0.887)	rs200771233	0
PLEKHO2	G>G/T	15	65134221	het	17	missense_variant	2	E/D	deleterious(0)	possibly_damaging(0.772)	rs201633413	0
CCDC88C	G>G/A	14	91780275	het	77	missense_variant	629	R/W	deleterious(0)	probably_damaging(0.943)		0
ABCD4	G>G/A	14	74756738	het	106	missense_variant	471	R/W	deleterious(0)	probably_damaging(0.966)	rs45568335	0.14
POTEG	G>G/A	14	19553852	het	10	missense_variant	146	A/T	deleterious(0)	probably_damaging(0.998)	rs138358738	0
POTEG	C>C/T	14	19553436	het	0	missense_variant	7	S/L	deleterious(0)	probably_damaging(0.951)	rs201747513	0
FAM124A	G>G/A	13	51825750	het	69	missense_variant	119	E/K	deleterious(0)	probably_damaging(0.998)	rs146836694	0.32
N4BP2L2	C>C/A	13	33018121	het	157	missense_variant	185	D/Y	deleterious(0)	probably_damaging(0.986)	rs201479580	0
TBX3	T>T/A	12	115109957	het	121	missense_variant	641	S/C	deleterious(0)	probably_damaging(0.94)		0
TMTC1	A>A/G	12	29936515	het	28	missense_variant	57	I/T	deleterious(0)	possibly_damaging(0.806)		0
TMTC1	C>C/T	12	29936501	het	27	missense_variant	62	D/N	deleterious(0)	benign(0.207)	rs76424334	0
TMTC1	C>C/A	12	29936449	het	12	missense_variant	79	W/L	deleterious(0)	possibly_damaging(0.868)		0
PLBD1	G>G/T	12	14720554	het	24	missense_variant	26	P/Q	deleterious(0)	unknown(0)	rs1141509	0
ACAD8	C>C/G	11	134128923	het	60	missense_variant	171	S/C	deleterious(0)	probably_damaging(0.986)	rs113488591	1
SLC22A10	G>G/T	11	63064887	het	139	missense_variant	207	G/C	deleterious(0)	probably_damaging(1)	rs117447942	1
GLYAT	G>G/A	11	58477418	het	144	missense_variant	238	R/W	deleterious(0)	possibly_damaging(0.673)		0
OR4C3	G>G/T	11	48347306	het	231	missense_variant	272	V/F	deleterious(0)	benign(0.43)	rs73465911	0
OR4C3	A>A/G	11	48346962	het	180	missense_variant	157	N/S	deleterious(0)	benign(0.002)	rs74589050	0
IGSF22	A>A/G	11	18745723	het	60	missense_variant	21	S/P	deleterious(0)	probably_damaging(0.937)		0
TPP1	G>G/A	11	6636488	het	108	missense_variant	447	R/C	deleterious(0)	probably_damaging(1)		0
TRPM5	G>G/C	11	2442361	het	65	missense_variant	122	D/E	deleterious(0)	probably_damaging(0.993)		0
MUC6	C>C/T	11	1028379	het	402	missense_variant	534	G/S	deleterious(0)	unknown(0)	rs116256283	0.27
MUC6	G>G/A	11	1017069	het	661	missense_variant	1911	T/M	deleterious(0)	probably_damaging(0.996)	rs80333708	0
LRRC56	G>G/A	11	551161	het	44	missense_variant	219	V/M	deleterious(0)	probably_damaging(0.994)	rs138291757	0
LIPN	C>C/T	10	90524335	het	136	missense_variant	132	S/L	deleterious(0)	benign(0.279)	rs41284088	1
ZNF365	T>T/G	10	64425927	het	102	missense_variant	420	L/R	deleterious(0)	possibly_damaging(0.87)	rs142270485	0
CACNA1B	C>C/G	9	140777306	het	224	missense_variant	167	N/K	deleterious(0)	benign(0.223)	rs4422842	0
ALDH1A1	C>C/A	9	75545852	het	69	missense_variant	85	R/S	deleterious(0)	probably_damaging(1)	rs202024021	0
CNTNAP3	G>G/T	9	39132949	het	8	missense_variant	687	A/E	deleterious(0)	benign(0.283)		0
WDR67	G>G/A	8	124164118	het	83	missense_variant	1047	R/H	deleterious(0)	probably_damaging(0.999)	rs181753396	0.05
TRPS1	C>C/G	8	116631392	het	122	missense_variant	311	R/S	deleterious(0)	probably_damaging(0.968)		0
TMEM67	T>T/C	8	94807731	het	134	missense_variant	590	F/S	deleterious(0)	probably_damaging(0.996)	rs267607115	0
TPD52	C>C/A	8	80976814	het	118	missense_variant	52	D/Y	deleterious(0)	probably_damaging(0.979)	rs35099105	1
PREX2	C>C/T	8	69058536	het	39	missense_variant	1394	R/W	deleterious(0)	probably_damaging(0.995)	rs61753703	1
RAB11FIP1	A>A/G	8	37720486	het	45	missense_variant	1260	V/A	deleterious(0)	probably_damaging(0.985)	rs151011205	1
MIOX	C>C/T	22	50927956	het	52	downstream_gene_variant	0				rs41280565	1
KIF13B	G>G/A	8	29024943	het	27	missense_variant	369	R/W	deleterious(0)	probably_damaging(1)	rs117139027	1
RRP7B	T>T/TA	22	42972515	het	11	downstream_gene_variant	0				rs34360734, rs1	0
POLR2F	G>G/C	22	38363693	het	74	downstream_gene_variant	0					0
FBXO16	A>A/G	8	28309846	het	64	missense_variant	219	W/R	deleterious(0)	probably_damaging(0.971)		0
ZAN	C>C/T	7	100371473	het	109	missense_variant	1922	R/C	deleterious(0)	probably_damaging(0.958)	rs314299	0
ASL	C>C/T	7	65547912	het	79	missense_variant	113	R/W	deleterious(0)	probably_damaging(1)		0
ROS1	G>G/A	6	117708999	het	105	missense_variant	653	S/F	deleterious(0)	probably_damaging(0.948)	rs34203286	0.32
UBE3D	G>G/T	6	83667038	het	122	missense_variant	381	S/Y	deleterious(0)	probably_damaging(0.995)	rs34566948	0.14
C6orf57	C>C/T	6	71298323	het	56	missense_variant	75	P/S	deleterious(0)	probably_damaging(0.999)	rs146446063	1
KIF6	C>C/T	6	39554152	het	191	missense_variant	292	R/H	deleterious(0)	probably_damaging(0.916)		0
TSPEAR,KRTA	ATC>ATC/TGA	21	45959557	het	145	intron_variant	0					0
DDX41	A>A/G	5	176939508	het	131	missense_variant	513	I/T	deleterious(0)	probably_damaging(0.991)		0
FAM134B	G>G/C	5	16477814	het	72	missense_variant	319	F/L	deleterious(0)	probably_damaging(0.984)		0
UMODL1	AGGTGGGTGCGGAGTGGGGTGGGAGGTGCAGGCTG	21	43557750	het	24	splice_donor_variant, 3_prime_UTR_variant, intron_variant, featu	0					0
TMEM144	A>A/T	4	159161483	het	152	missense_variant	239	I/F	deleterious(0)	probably_damaging(0.996)	rs62335898	0.41
LRIT3	C>C/A	4	110773048	het	155	missense_variant	169	P/T	deleterious(0)	possibly_damaging(0.862)	rs61745483	2
SH3TC1	C>C/A	4	8235220	het	23	missense_variant	1088	L/M	deleterious(0)	probably_damaging(0.998)	rs111285143	1
SH3BP2	C>C/T	4	2834080	het	107	missense_variant	534	R/W	deleterious(0)	possibly_damaging(0.609)	rs148761331	0.27
BCHE	C>C/T	3	165548238	het	208	missense_variant	195	G/D	deleterious(0)	probably_damaging(0.997)		0
VEPH1	C>C/T	3	157099020	het	46	missense_variant	351	R/H	deleterious(0)	probably_damaging(0.929)		0
COL6A6	C>C/A	3	130282383	het	163	missense_variant	179	T/K	deleterious(0)	probably_damaging(0.999)	rs114511272	1
ZNF717	C>C/T	3	75786753	het	52	missense_variant	674	R/H	deleterious(0)	benign(0.01)	rs3009020, rs74	0
ZNF717	G>G/T	3	75786684	het	26	missense_variant	697	T/K	deleterious(0)	benign(0.004)	rs201345045	0
ZNF717	G>G/A	3	75786681	het	26	missense_variant	698	P/L	deleterious(0)	benign(0.006)	rs111677009, rs	0
COL7A1	G>G/A	3	48630087	het	173	missense_variant	298	R/W	deleterious(0)	unknown(0)		0
FKBP1A-SDC	C>C/T	20	1293141	het	196	downstream_gene_variant	0				rs35367003	1
COL4A4	C>C/G	2	227946893	het	149	missense_variant	545	G/A	deleterious(0)	probably_damaging(0.996)	rs1800516	3
TNS1	T>T/G	2	218758200	het	80	missense_variant	102	N/H	deleterious(0)	possibly_damaging(0.845)	rs61745748	1
POTEE	G>G/A	2	132021946	het	0	missense_variant	973	G/D	deleterious(0)	probably_damaging(0.999)		0
ACOXL	C>C/T	2	111562875	het	119	missense_variant	219	S/L	deleterious(0)	possibly_damaging(0.711)	rs77331476	1
APLF	C>C/A	2	68794483	het	34	missense_variant	433	Q/K	deleterious(0)	possibly_damaging(0.877)	rs36021078	2
FBXO48	A>A/C	2	68691366	het	181	missense_variant	148	I/S	deleterious(0)	probably_damaging(0.991)		0
GPR113	T>T/C	2	26534250	het	187	missense_variant	782	I/M	deleterious(0)	probably_damaging(0.941)	rs114354727	2
C1orf27	A>A/C	1	186368092	het	22	missense_variant	306	K/Q	deleterious(0)	probably_damaging(0.999)	rs76602544	1
NBPF14,NBP	G>G/C	1	145293490	het	20	missense_variant	29	A/P	deleterious(0)	benign(0.339)	rs6671335	0
NBPF14,NOT	C>C/A	1	145281633	het	475	missense_variant	188	P/H	deleterious(0)	possibly_damaging(0.89)	rs28576333	0
NBPF14,NOT	C>C/T	1	145281408	het	899	missense_variant	113	P/L	deleterious(0)	probably_damaging(0.999)	rs201164506	0
PPIAL4G	C>C/T	1	143767629	het	15	missense_variant	74	G/S	deleterious(0)	possibly_damaging(0.876)		0
UBXN11	T>T/C	1	26620806	het	143	missense_variant	150	Y/C	deleterious(0)	probably_damaging(0.999)	rs56039743	1
MYOM3	G>G/T	1	24384114	het	91	missense_variant	1352	L/M	deleterious(0)	probably_damaging(0.999)	rs201441408	0
PADI4	A>A/G	1	17668888	het	58	missense_variant	309	Y/C	deleterious(0)	possibly_damaging(0.865)	rs33981382	1
HNRNPCL1	G>G/C	1	12907285	het	43	missense_variant	286	S/R	deleterious(0)	benign(0.002)	rs148930640	0
PRAMEF1	A>A/C	1	12854474	het	184	missense_variant	233	N/T	deleterious(0)	benign(0.06)	rs1063774	0
UBE4B	G>G/C	1	10228220	het	76	missense_variant	1075	Q/H	deleterious(0)	probably_damaging(0.999)	rs147961171	1
PRDM16	C>C/T	1	3348603	het	46	missense_variant	1199	R/C	deleterious(0)	probably_damaging(0.938)		0
MORN1	G>G/A	1	2290143	het	63	missense_variant	253	R/W	deleterious(0)	probably_damaging(0.996)	rs34587196	1
LOC1009964	C>C/T	X	153154020	het	94	missense_variant	154	T/M		benign(0.024)		0
C22orf26	G>G/C	22	46449891	het	32	missense_variant	28	P/R		unknown(0)	rs12159707	0
BAGE2	T>T/C	21	11058229	het	327	missense_variant	71	T/A			rs55883018	0
BAGE2	G>G/C	21	11058226	het	328	missense_variant	72	P/A			rs4913558	0
CD177	G>G/A	19	43865333	het	2	missense_variant	267	A/T			rs201266439	0
PSG7	C>C/T	19	43430060	het	142	missense_variant	248	G/R			rs201797371	0
MYO9B	G>G/A	19	17311226	het	18	missense_variant	1455	G/S			rs117099942	2
MUC16	T>T/C	19	9002504	het	84	missense_variant	13438	N/D		probably_damaging(0.962)	rs78327556	0
KATNAL2,TCE	G>G/C	18	44555312	het	61	intron_variant	0				rs76539063	0
FLJ22184	G>G/T	19	7935863	het	20	missense_variant	756	L/I			rs5027409	0
SLC35G4	A>A/G	18	11610024	het	99	missense_variant	144	I/V			rs80224205	0
SLC35G4	A>A/G	18	11610001	het	148	missense_variant	136	H/R			rs79348909	0
SLC35G4	C>C/T	18	11609994	het	159	missense_variant	134	R/C			rs75154847	0
DNAH17	C>C/T	17	76437163	het	117	missense_variant	3883	V/I			rs150140927	0
NPIPB15	G>G/A	16	74425358	het	24	missense_variant	160	A/T			rs6564065	0
MRPL12	C>C/G	17	79673994	het	184	downstream_gene_variant	0					0
AATK	G>G/A	17	79094875	het	151	downstream_gene_variant	0					0
SETD1A	T>T/A	16	30976129	het	129	missense_variant	356	S/T		benign(0.192)	rs117891062	2
RECQL5	G>G/T	17	73626923	het	59	downstream_gene_variant	0				rs74948442	0
RECQL5	G>G/GTGT	17	73626922	het	57	downstream_gene_variant	0					0
RECQL5	T>T/TGT	17	73626921	het	54	downstream_gene_variant	0					0
PRR25	A>A/G	16	855885	het	26	missense_variant	148	K/R		possibly_damaging(0.627)	rs147922685	1
PCSK6	C>C/T	15	101924576	het	59	missense_variant	455	G/S			rs74032814	1
C15orf32	C>C/G	15	93043627	het	119	missense_variant	178	S/R		possibly_damaging(0.66)		0
SCRN2	T>T/C	17	45917605	het	111	downstream_gene_variant	0				rs17856536	1
LOC440292	C>C/G	15	78234093	het	108	missense_variant	219	G/R			rs112736682	0.41
LOC642131	T>T/C	15	22473167	het	89	missense_variant	35	E/G			rs201318253	0
LOC642131	A>A/G	15	22473146	het	118	missense_variant	42	V/A			rs75509601	0
LOC642131	G>G/T	15	22473042	het	139	missense_variant	77	P/T			rs72687775	0
GPR179	G>G/T	17	36483522	het	94	downstream_gene_variant	0				rs62073368	1
ZFP36L1	C>C/T	14	69259732	het	21	missense_variant	44	G/E				0
LOC1001322	T>T/C	14	19118308	het	64	missense_variant	493	L/P			rs201813899	0
LOC1001322	T>T/A	14	19118254	het	181	missense_variant	475	V/E			rs140014336	0
LOC1001322	T>T/A	14	19118237	het	206	missense_variant	469	N/K			rs28526835	0
LOC1001322	C>C/G	14	19118232	het	204	missense_variant	468	Q/E			rs147013435	0
LOC1001322	A>A/G	14	19118192	het	202	missense_variant	454	I/M			rs77434589	0
CCDC168	T>T/C	13	103388880	het	284	missense_variant	4723	M/V			rs17592459	2
CCDC168	C>C/G	13	103388015	het	195	missense_variant	5011	G/A			rs17507841	2
CCDC168	G>G/A	13	103384712	het	75	missense_variant	6112	T/M			rs17507827	1
CD68	C>C/T	17	7484716	het	209	downstream_gene_variant	0				rs200380131	0
SENP3	C>C/T	17	7468277	het	111	downstream_gene_variant	0				rs35596387	2
SPNS2	T>T/TGG	17	4439727	het	38	downstream_gene_variant	0				rs3053652	0
DOCK9	G>G/A	13	99554621	het	108	missense_variant	435	A/V			rs62620184	2
CCER1	C>C/T	12	91347643	het	99	missense_variant	293	E/K		unknown(0)	rs78240650	2

IL17C	G>G/A	16	88706385	het	107	downstream_gene_variant	0			rs199990283	0.23
KMT2D	G>G/A	12	49433883	het	116	missense_variant	2557	P/L	unknown(0)	rs189888707	1
MUC19	G>G/A	12	40928524	het	16	missense_variant	7255	R/Q			0
OVOS2	A>A/T	12	31282765	het	58	missense_variant	1040	F/Y		rs2536839	0
OVOS	G>G/A	12	9723336	het	24	missense_variant	1226	D/N		rs200842139	0
SAA2-SAA4,S	G>G/A	11	18267478	het	173	missense_variant	70	A/V		rs79620496	0
SAA2-SAA4,S	A>A/G	11	18267463	het	172	missense_variant	75	V/A		rs74872559	0
OR56A5	C>C/A	11	5989415	het	65	missense_variant	104	V/L		rs7114672	0
MUC5B	C>C/T	11	1272800	het	72	missense_variant	4897	S/L	unknown(0)	rs55693520	2
MUC5B	C>C/G	11	1271321	het	50	missense_variant	4404	A/G	unknown(0)	rs2943517	0
MUC5B	A>A/G	11	1253980	het	91	missense_variant	682	D/G	unknown(0)	rs202127660	0
MUC5B	A>A/G	11	1253976	het	102	missense_variant	681	S/G	unknown(0)	rs76956995	0
MUC5AC	A>A/G	11	1213424	het	570	missense_variant	774	N/S		rs61867531	0
MUC5AC	G>G/A	11	1213405	het	629	missense_variant	768	E/K		rs67262263	0
MUC5AC	C>C/G	11	1213204	het	276	missense_variant	701	L/V		rs72479396	0
MUC2	G>G/C	11	1093582	het	72	missense_variant	1800	A/P		rs55641679	0
MUC2	A>A/G	11	1093057	het	82	missense_variant	1625	T/A		rs11245947	0
MUC2	C>C/G	11	1092872	het	192	missense_variant	1563	T/S		rs113722672	0
FAHD1,MEIO	C>C/A	16	1884292	het	146	intron_variant	0				0
LOC1009966	T>T/C	9	66457076	het	51	missense_variant	164	K/R		rs76994164	0
LOC1009966	G>G/C	9	66455678	het	27	missense_variant	199	T/R		rs79224684	0
LOC1009966	T>T/G	9	66455668	het	33	missense_variant	202	K/N		rs78797086	0
ANKRD18B	G>G/C	9	33541216	het	61	missense_variant	294	G/R		rs111814125	0
SSPO	C>C/T	7	149502657	het	41	missense_variant	2825	P/L		rs199913741	0
MUC17	A>A/G	7	100683053	het	332	missense_variant	2786	T/A	possibly_damaging(0.626)	rs200821451	0
MUC17	T>T/C	7	100683036	het	334	missense_variant	2780	I/T	benign(0.003)	rs78879527	0
MUC17	T>T/C	7	100680117	het	401	missense_variant	1807	M/T	unknown(0)	rs147353603	0
MUC17	G>G/A	7	100679760	het	174	missense_variant	1688	G/E	unknown(0)	rs73168394	0
MUC17	C>C/G	7	100679754	het	176	missense_variant	1686	T/S	unknown(0)	rs4992073	0
MUC17	A>A/G	7	100679254	het	394	missense_variant	1519	I/M	unknown(0)	rs199808245	0
MUC17	G>G/T	7	100678977	het	273	missense_variant	1427	G/V	unknown(0)	rs148743807	0
MUC17	A>A/G	7	100678932	het	262	missense_variant	1412	E/G	unknown(0)	rs114941002	0
MUC17	G>G/A	7	100678622	het	332	missense_variant	1309	V/M	unknown(0)	rs77299546	0
MUC17	G>G/A	7	100678616	het	328	missense_variant	1307	G/S	unknown(0)	rs113959201	0
ELL3	C>C/T	15	44069096	het	133	downstream_gene_variant	0				0
MUC17	A>A/T	7	100678610	het	336	missense_variant	1305	T/S	unknown(0)	rs78010183	0
MUC17	G>G/A	7	100678013	het	1144	missense_variant	1106	V/M	unknown(0)	rs75312831	0
MUC17	C>C/T	7	100677704	het	808	missense_variant	1003	L/F	unknown(0)	rs78330257	0
MUC17	C>C/A	7	100677645	het	798	missense_variant	983	T/N	unknown(0)	rs114262718	0
MUC17	A>A/G	7	100677572	het	721	missense_variant	959	T/A	unknown(0)	rs60940057	0
MUC17	C>C/A	7	100677378	het	379	missense_variant	894	T/K	unknown(0)	rs143956720	0
MUC17	G>G/A	7	100677285	het	375	missense_variant	863	G/E	unknown(0)	rs74852422	0
MUC12	C>C/T	7	100647676	het	5	missense_variant	4611	S/L	unknown(0)	rs113138639	0
MUC12	C>C/T	7	100646824	het	353	missense_variant	4327	T/I	unknown(0)	rs201628729	0
MUC12	G>G/A	7	100639054	het	26	missense_variant	1737	R/H	unknown(0)	rs56353590	0
METTL21D	T>T/A	14	50583200	het	189	downstream_gene_variant	0				0
MUC12	C>C/A	7	100636207	het	21	missense_variant	788	T/N	unknown(0)	rs147681181	0
MUC3A	C>C/T	7	100607871	het	79	missense_variant	1283	A/V		rs6960868	0
MUC3A	A>A/G	7	100552739	het	379	missense_variant	1107	Q/R		rs75517157	0
MUC3A	G>G/C	7	100552727	het	382	missense_variant	1103	R/P		rs74529310	0
MUC3A	T>T/A	7	100552711	het	360	missense_variant	1098	S/T		rs75799835	0
MUC3A	C>C/T	7	100552675	het	374	missense_variant	1086	P/S		rs73163764	0
MUC3A	A>A/G	7	100552657	het	369	missense_variant	1080	T/A		rs75471530	0
MUC3A	T>T/C	7	100552550	het	296	missense_variant	1044	L/P		rs73163762	0
MUC3A	C>C/A	7	100552549	het	297	missense_variant	1044	L/I		rs73163760	0
MUC3A	G>G/A	7	100552535	het	267	missense_variant	1039	S/N		rs73163758	0
MUC3A	C>C/T	7	100552436	het	184	missense_variant	1006	S/L		rs78685360	0
MUC3A	T>T/A	7	100552435	het	186	missense_variant	1006	S/T		rs76244845	0
MUC3A	T>T/C	7	100552412	het	186	missense_variant	998	M/T		rs73714259	0
MUC3A	T>T/C	7	100552390	het	175	missense_variant	991	Y/H		rs73714257	0
MUC3A	A>A/G	7	100552384	het	179	missense_variant	989	T/A		rs75597914	0
MUC3A	C>C/G	7	100552371	het	191	missense_variant	984	I/M		rs73714255	0
MUC3A	C>C/G	7	100552358	het	195	missense_variant	980	T/S		rs73714254	0
MUC3A	T>T/C	7	100552135	het	262	missense_variant	906	S/P		rs62483700	0
MUC3A	G>G/T	7	100552117	het	296	missense_variant	900	A/S		rs62483699	0
MUC3A	T>T/G	7	100552018	het	581	missense_variant	867	S/A		rs76249962	0
MUC3A	C>C/T	7	100551842	het	477	missense_variant	808	T/I		rs75365561	0
MUC3A	G>G/T	7	100551793	het	397	missense_variant	792	V/F		rs73398735	0
MUC3A	G>G/A	7	100551778	het	338	missense_variant	787	V/M		rs73714247	0
MUC3A	C>C/A	7	100551773	het	329	missense_variant	785	T/K		rs73398734	0
MUC3A	C>C/T	7	100551719	het	231	missense_variant	767	T/M		rs73714243	0
MUC3A	T>T/C	7	100551626	het	218	missense_variant	736	I/T		rs73714238	0
MUC3A	T>T/G	7	100551578	het	233	missense_variant	720	I/S		rs78584246	0
MUC3A	T>T/C	7	100551566	het	252	missense_variant	716	M/T		rs78538898	0
MUC3A	A>A/G	7	100551565	het	256	missense_variant	716	M/V		rs73163757	0
MUC3A	C>C/T	7	100551476	het	260	missense_variant	686	T/I		rs74460367	0
MUC3A	G>G/C	7	100551473	het	254	missense_variant	685	R/P		rs79233494	0
MUC3A	C>C/G	7	100551461	het	246	missense_variant	681	T/S		rs78826835	0
MUC3A	T>T/C	7	100551433	het	220	missense_variant	672	F/L		rs73398732	0
MUC3A	G>G/A	7	100550873	het	85	missense_variant	485	R/H		rs74836112	0
MUC3A	T>T/C	7	100550786	het	34	missense_variant	456	L/S		rs79714278	0
MUC3A	C>C/A	7	100550428	het	89	missense_variant	337	P/T		rs4729627	0
MUC3A	A>A/G	7	100550252	het	137	missense_variant	278	Q/R		rs67587735	0
MUC3A	G>G/A	7	100550245	het	143	missense_variant	276	A/T		rs66732041	0
MUC3A	C>C/T	7	100550138	het	252	missense_variant	240	T/M		rs73714233	0
MUC3A	C>C/T	7	100549979	het	354	missense_variant	187	T/I		rs73398717	0
MUC3A	T>T/C	7	100549942	het	347	missense_variant	175	S/P		rs73714230	0
MUC3A	T>T/G	7	100549935	het	339	missense_variant	172	S/R		rs73714229	0
MUC3A	G>G/A	7	100549873	het	337	missense_variant	152	A/T		rs74193966	0
MUC3A	C>C/T	7	100549787	het	482	missense_variant	123	S/L		rs73714227	0
MUC3A	C>C/T	7	100549703	het	502	missense_variant	95	P/L		rs78164286	0
MUC3A	A>A/G	7	100549697	het	478	missense_variant	93	N/S		rs77036391	0
DNAH11	G>G/A	7	21775369	het	257	missense_variant	2525	S/N		rs68023059	2
DNAH11	C>C/G	7	21640393	het	131	missense_variant	1034	L/V			0
OFCC1	C>C/T	6	9769187	het	132	missense_variant	645	G/R		rs147413800	1
TAS2R14	T>T/C	12	11091551	het	135	downstream_gene_variant	0			rs16925868	2
MCC	C>C/T	5	112824054	het	55	missense_variant	20	G/S	unknown(0)	rs199741976	0
ZDHHC11B	G>G/A	5	767588	het	23	missense_variant	61	A/V		rs507953	0
ZDHHC11B	G>G/T	5	741736	het	32	missense_variant	548	A/D		rs61128505	0
GIMD1	C>C/T	4	107288385	het	160	missense_variant	70	V/I		rs189805569	0.05
SPP1	A>A/G	4	88903825	het	179	missense_variant	254	K/R		rs149833253	0.09
DSPP	A>A/G	4	88537035	het	45	missense_variant	1074	D/G	unknown(0)	rs202210195	0
DSPP	G>G/A	4	88533867	het	183	missense_variant	177	A/T	benign(0.008)		0
CRIPAK	T>T/C	4	1389156	het	104	missense_variant	286	M/T	benign(0)	rs71614972	0
PDZD3	T>T/C	11	119058354	het	21	downstream_gene_variant	0				0
MUC4	A>A/T	3	195515449	het	36	missense_variant	1001	V/E	possibly_damaging(0.469)	rs200672669	0
MUC4	T>T/G	3	195513502	het	28	missense_variant	1650	H/P	benign(0.365)	rs74664428	0
MUC4	T>T/C	3	195512468	het	14	missense_variant	1995	T/A	probably_damaging(0.939)		0
MUC4	G>G/A	3	195512287	het	14	missense_variant	2055	S/F	probably_damaging(0.98)	rs113602668	0
MUC4	G>G/T	3	195512212	het	19	missense_variant	2080	P/H	probably_damaging(0.94)	rs75588776	0
C11orf48	A>A/T	11	62435162	het	47	downstream_gene_variant	0				0
MUC4	T>T/C	3	195511814	het	35	missense_variant	2213	S/G	benign(0.187)	rs72499650	0
MUC4	T>T/C	3	195511142	het	14	missense_variant	2437	N/D	benign(0.322)	rs430037	0
MUC4	C>C/T	3	195510827	het	20	missense_variant	2542	A/T	possibly_damaging(0.521)	rs413807	0
MUC4	A>A/G	3	195510773	het	17	missense_variant	2560	S/P	benign(0.353)	rs2911272	0
MUC4	C>C/A	3	195507242	het	16	missense_variant	3737	A/S	possibly_damaging(0.574)	rs112808291	0
ART5	G>G/A	11	3660238	het	35	downstream_gene_variant	0			rs142998376	0.23
MUC4	A>A/G	3	195507226	het	19	missense_variant	3742	V/A	benign(0.012)	rs201269328	0
MUC4	A>A/G	3	195507166	het	20	missense_variant	3762	L/P	benign(0.001)	rs79358488	0
MUC4	C>C/T	3	195507107	het	18	missense_variant	3782	A/T	possibly_damaging(0.509)	rs201895754	0
MUC4	C>C/T	3	195507062	het	13	missense_variant	3797	D/N	possibly_damaging(0.636)	rs199822551	0
MUC4	G>G/C	3	195505788	het	40	missense_variant	4221	H/Q	benign(0.262)	rs2550253	0
RYK	T>T/C	3	133896860	het	18	missense_variant	413	I/V			0
CRYBG3	C>C/T	3	97634570	het	63	missense_variant	2744	P/L			0
ARIH2OS	A>A/G	3	48956257	het	79	missense_variant	109	V/A	benign(0.336)	rs150858838	1
CIDEC	G>G/A	3	9918811	het	38	missense_variant	62	T/M		rs61742367	2
TTN	C>C/T	2	179606172	het	167	missense_variant	3930	E/K		rs186624523	0.18
ZNF806	A>A/G	2	133076229	het	267	missense_variant	617	K/E		rs2598806	0
SIGIRR	C>C/A	11	407163	het	13	downstream_gene_variant	0				0
RIC8A	C>C/G	11	210660	het	87	downstream_gene_variant	0			rs34925440	1
ZNF806	G>G/A	2	133076221	het	291	missense_variant	614	R/H		rs7340213	0
ZNF806	C>C/T	2	133076206	het	283	missense_variant	609	A/V		rs2598807	0
ZNF806	G>G/A	2	133076155	het	302	missense_variant	592	C/Y		rs2677622	0
ZNF806	T>T/G	2	133076032	het	206	missense_variant	551	F/C		rs2598809	0
ZNF806	A>A/G	2	133075726	het	60	missense_variant	449	E/G		rs2598810	0
ZNF806	G>G/C	2	133075674	het	107	missense_variant	432	G/R		rs7340192	0
ZNF806	T>T/G	2	133075664	het	132	missense_variant	428	D/E		rs7340499	0
ZNF806	G>G/T	2	133075639	het	159	missense_variant	420	G/V		rs7340191	0

ZNF806	G>G/C	2	133075615	het	197	missense_variant	412	C/S			rs76315261	0
ZNF806	C>C/T	2	133075612	het	207	missense_variant	411	P/L			rs11491243	0
ZNF806	G>G/A	2	133075386	het	110	missense_variant	336	G/R			rs7349216	0
ZNF806	G>G/A	2	133075365	het	98	missense_variant	329	V/I			rs7349215	0
ZNF806	T>T/C	2	133075282	het	174	missense_variant	301	V/A			rs7349446	0
ZNF806	C>C/T	2	133075207	het	179	missense_variant	276	A/V			rs7349365	0
ZNF806	C>C/T	2	133075104	het	172	missense_variant	242	R/C			rs7349364	0
ZNF806	A>A/G	2	133075069	het	185	missense_variant	230	H/R			rs72991565	0
ZNF806	G>G/A	2	133075027	het	204	missense_variant	216	G/E			rs7349198	0
ZNF806	A>A/G	2	133074876	het	39	missense_variant	166	M/V			rs7355689	0
ZNF806	A>A/C	2	133074759	het	141	missense_variant	127	K/Q			rs62169564	0
ZNF806	A>A/T	2	133074745	het	158	missense_variant	122	E/V			rs7355686	0
ZNF806	T>T/G	2	133074727	het	178	missense_variant	116	I/S			rs74763985	0
ZNF806	A>A/C	2	133074718	het	178	missense_variant	113	K/T			rs4953961	0
ZNF806	T>T/C	2	133070556	het	160	missense_variant	97	F/L			rs7355766	0
ZNF806	G>G/T	2	133070508	het	265	missense_variant	81	A/S			rs7340197	0
ZNF806	T>T/C	2	133070449	het	294	missense_variant	61	V/A			rs2459647	0
ZNF806	G>G/A	2	133066879	het	71	missense_variant	53	G/S			rs2677610	0
ANKRD36C	C>C/T	2	96619734	het	198	missense_variant	352	A/T			rs62154618	0
ANKRD36C	G>G/T	2	96610420	het	173	missense_variant	448	D/E			rs78473403	0
ANKRD36C	C>C/T	2	96610413	het	181	missense_variant	451	E/K			rs74491979	0
ANKRD36C	T>T/A	2	96610410	het	181	missense_variant	452	N/Y			rs77220524	0
ANKRD36C	C>C/A	2	96610399	het	190	missense_variant	455	L/F			rs116594293	0
ANKRD36C	A>A/T	2	96604632	het	500	missense_variant	492	S/R			rs62154562	0
ANKRD36C	C>C/A	2	96604606	het	624	missense_variant	501	G/V			rs80350011	0
ANKRD36C	A>A/G	2	96604591	het	636	missense_variant	506	L/P			rs62154561	0
SRGAP2	T>T/C	1	206579798	het	3	missense_variant	322	L/S			rs200703951	0
SRGAP2	C>C/T	1	206566195	het	202	missense_variant	127	T/M			rs200978831	0
FAM58BP	A>A/G	1	200182732	het	26	missense_variant	14	D/G			rs116503230	0
PRG4	A>A/T	1	186275756	het	97	missense_variant	302	E/V		unknown(0)		0
TOR1AIP1	C>C/T	1	179852065	het	87	missense_variant	143	S/F			rs141391430	0
HRNR	C>C/T	1	152188998	het	90	missense_variant	1703	G/S		unknown(0)	rs80018286	0
HRNR	C>C/T	1	152188964	het	92	missense_variant	1714	S/N		unknown(0)	rs78582525	0
RHOBTB2	C>C/T	8	22874927	het	47	downstream_gene_variant	0				rs146723409	0.14
HRNR	G>G/A	1	152188176	het	259	missense_variant	1977	R/C		unknown(0)	rs147618491	0
HRNR	T>T/A	1	152188109	het	55	missense_variant	1999	Q/L		unknown(0)	rs61814940	0
HRNR	C>C/T	1	152187588	het	73	missense_variant	2173	G/S		unknown(0)	rs201071455	0
HRNR	G>G/A	1	152186766	het	161	missense_variant	2447	R/C		unknown(0)	rs4845747	0
LOC1009963	T>T/G	1	120926044	het	12	missense_variant	250	L/R				0
WDR65	G>G/A	1	43708928	het	53	missense_variant	1179	E/K			rs185084915	0.05
AIM1L	A>A/G	1	26671690	het	63	missense_variant	487	S/P			rs112674559	0
VWA5B1	C>C/A	1	20662944	het	76	missense_variant	636	A/D			rs147563081	0.05
NBPF1	G>G/A	1	16918498	het	27	missense_variant	7	P/S			rs11583018	0
NBPF1	C>C/T	1	16907303	het	113	missense_variant	510	A/T			rs201805881	0
NBPF1	T>T/C	1	16890671	het	14	missense_variant	1046	M/V			rs2419526	0
POLR2J2,UPK	C>C/T	7	102279621	het	186	3_prime_UTR_variant	0				rs201558735	0
NBPF1	C>C/T	1	16890644	het	26	missense_variant	1055	V/I			rs2992339	0
NBPF1	T>T/A	1	16890598	het	48	missense_variant	1070	Y/F			rs2990550	0
NBPF1	G>G/C	1	16890484	het	39	missense_variant	1108	T/R			rs12117084	0
LOC1005065	T>T/G	1	16863328	het	224	missense_variant	134	V/G			rs61769543	0
LOC1005065	A>A/G	1	16862502	het	817	missense_variant	75	N/D			rs141629643	0
LOC1005065	T>T/A	1	16862461	het	798	missense_variant	61	V/D			rs145386412	0
LOC1005065	T>T/G	1	16862454	het	731	missense_variant	59	S/A			rs71248115	0
HNRNPCP5	C>C/T	1	13183533	het	180	missense_variant	114	G/S			rs77556803	0
HNRNPCP5	C>C/T	1	13183532	het	180	missense_variant	114	G/D			rs77505369	0
HNRNPCP5	A>A/C	1	13183528	het	182	missense_variant	115	F/L			rs76937665	0
HNRNPCP5	T>T/C	1	13183511	het	185	missense_variant	121	D/G			rs72864546	0
RP1L1	C>C/CTCTCTTCTTGCAGCCCTTCTATTACTTTAGTCCC	8	10467604	het	241	missense_variant, feature_elongation	1335	G/VVQLEGTKETEEGLQEEGVQLEGTKVIEGLQEER				0
PRSS3	A>A/T	9	33796799	het	148	missense_variant, splice_region_variant	124	T/S	tolerated(1)	benign(0.002)	rs200709040	0
MACF1	G>G/T	1	39758439	het	41	missense_variant, splice_region_variant	644	G/V	tolerated(0.93)	possibly_damaging(0.78)	rs139995582	1
GYPE	C>C/G	4	144801662	het	346	missense_variant, splice_region_variant	13	G/A	tolerated(0.77)	benign(0)	rs1132785, rs2	0
CROCC	C>C/A	1	17266586	het	11	missense_variant, splice_region_variant	602	S/R	tolerated(0.41)	unknown(0)	rs45452999	1
CLCN7	A>A/G	16	1505251	het	64	missense_variant, splice_region_variant	328	F/L	tolerated(0.24)	benign(0.146)		0
UBR1	T>T/C	15	43250212	het	44	missense_variant, splice_region_variant	1612	R/G	tolerated(0.11)	benign(0.28)	rs78948790	0.32
KIAA0226	G>G/A	3	197409341	het	83	missense_variant, splice_region_variant	709	T/M	tolerated(0.1)	possibly_damaging(0.846)	rs35071969	1
PARP4	A>A/G	13	25021323	het	27	missense_variant, splice_region_variant	1039	I/T	deleterious(0.01)	possibly_damaging(0.78)	rs73172125	0
AQP7	G>G/T	9	33385863	het	12	missense_variant, splice_region_variant	176	A/E	deleterious(0.01)	benign(0.048)	rs62542746	0
ANKRD36	G>G/A	2	97877478	het	179	missense_variant, splice_region_variant	1157	V/M	deleterious(0.01)	probably_damaging(0.997)	rs10194525	0
ATP12A	G>G/A	13	25266567	het	8	missense_variant, splice_region_variant	363	V/M	deleterious(0)	probably_damaging(0.921)		0
ZNF705G	C>C/T	8	7218632	het	11	missense_variant, splice_region_variant	47	G/R	deleterious(0)	benign(0.038)	rs3989698	0
COL5A2	G>G/A	2	189918622	het	61	missense_variant, splice_region_variant	833	P/L	deleterious(0)	unknown(0)	rs116298748	1
SSFA2	G>G/A	2	182763839	het	88	missense_variant, splice_region_variant	168	S/N	deleterious(0)	probably_damaging(0.996)		0
ANKRD36C	T>T/G	2	96619688	het	133	missense_variant, splice_region_variant	367	E/A			rs74946480	0
ANKRD36C	T>T/G	2	96610921	het	636	missense_variant, splice_region_variant	421	N/T			rs62154594	0
SRGAP2	T>T/C	1	206566904	het	180	missense_variant, splice_region_variant	150	V/A			rs2919105	0
RFPL3,RFPL3	C>C/T	22	32756703	het	165	non_coding_exon_variant, nc_transcript_variant	0				rs73166367	0
RFPL3,RFPL3	A>A/G	22	32756652	het	113	non_coding_exon_variant, nc_transcript_variant	0				rs201161881	0
FEZF1,FEZF1-	T>T/C	7	121943941	het	91	non_coding_exon_variant, nc_transcript_variant	0					0
RINT1,EFCAB	C>C/A	7	105205815	het	85	non_coding_exon_variant, nc_transcript_variant	0				rs35531972	1
HYDIN	C>C/A	16	70935106	het	26	splice_acceptor_variant	0				rs113561245	0
PARP4	C>C/T	13	25009613	het	34	splice_acceptor_variant	0				rs201251739	0
KIF18A	C>C/T	11	28045398	het	23	splice_acceptor_variant	0				rs148846163	0.05
TTC40	T>T/C	10	134627760	het	139	splice_acceptor_variant	0				rs41306834	0.27
CNGA3	G>G/C	2	98986401	het	75	splice_acceptor_variant	0					0
ANKRD36C	C>C/G	2	96610923	het	618	splice_acceptor_variant	0				rs75080810	0
CROCCP2	T>T/C	1	16946535	het	13	splice_acceptor_variant, nc_transcript_variant	0				rs437905	0
IFI27L1	G>G/A	14	94567118	het	37	splice_donor_variant	0				rs144379529	0.14
CLEC12A	G>G/A	12	10124287	het	12	splice_donor_variant	0				rs148336258	0.05
RMDN2	G>G/A	2	38179345	het	104	splice_donor_variant	0				rs201107537	0
ALOX5AP	G>G/GTA	13	31287979	het	110	splice_donor_variant, feature_elongation	0				rs147763360	0
HMCN2	GTACGGGGACACCCACCCTCTGGCCACACCGCTGCA	9	133305073	het	20	splice_donor_variant, intron_variant, feature_truncation	0					0
MST1L	AC>AC/A	1	17087244	het	160	splice_donor_variant, nc_transcript_variant, feature_truncation	0				rs58502471	0
FLNA	C>C/T	X	153583083	het	54	splice_region_variant, intron_variant	0					0
IDH3G	G>G/A	X	153052407	het	138	splice_region_variant, intron_variant	0				rs185021909	0.06
SLC6A8	C>C/T	X	152960526	het	63	splice_region_variant, intron_variant	0				rs150207268	0.42
RPGR	C>C/T	X	38160494	het	23	splice_region_variant, intron_variant	0					0
MAP3K15	A>A/G	X	19418784	het	134	splice_region_variant, intron_variant	0					0
PES1	C>C/A	22	30975119	het	79	splice_region_variant, intron_variant	0				rs4395340	0.32
UBASH3A	G>G/A	21	43846802	het	89	splice_region_variant, intron_variant	0				rs2212836	2
UBASH3A	T>T/C	21	43846801	het	89	splice_region_variant, intron_variant	0				rs973344	2
BAGE2	C>C/A	21	11038722	het	161	splice_region_variant, intron_variant	0				rs62209718	0
WDR62	C>C/T	19	36575654	het	56	splice_region_variant, intron_variant	0				rs143309981	1
HAMP	G>G/A	19	35775758	het	81	splice_region_variant, intron_variant	0				rs201587159	0
LSR	C>C/T	19	35740038	het	10	splice_region_variant, intron_variant	0				rs149370311	0.37
RHPN2	A>A/T	19	33490616	het	175	splice_region_variant, intron_variant	0				rs199764566	0
MUC16	T>T/C	19	9005556	het	34	splice_region_variant, intron_variant	0				rs77817869	0
MUC16	T>T/C	19	9002493	het	75	splice_region_variant, intron_variant	0				rs77972891	0
RNF165	C>C/T	18	44013476	het	23	splice_region_variant, intron_variant	0					0
RBBP8	A>A/G	18	20516929	het	51	splice_region_variant, intron_variant	0				rs149189755	0.05
CCL23	C>C/T	17	34340777	het	28	splice_region_variant, intron_variant	0				rs113634985	0.46
KCNJ12	G>G/C	17	21318595	het	68	splice_region_variant, intron_variant	0				rs58780973	0
MAP2K3	G>G/T	17	21204308	het	357	splice_region_variant, intron_variant	0				rs55777930	0
FANCA	C>C/T	16	89815060	het	184	splice_region_variant, intron_variant	0				rs185527578	0
GLG1	A>A/G	16	74566056	het	10	splice_region_variant, intron_variant	0				rs192367831	0.09
IRX6	A>A/G	16	55360244	het	25	splice_region_variant, intron_variant	0				rs201683143	0.14
ANKDD1A	T>T/C	15	65236843	het	38	splice_region_variant, intron_variant	0					0
CATSPER2	T>T/G	15	43925135	het	22	splice_region_variant, intron_variant	0				rs199882327	0
HERC2	G>G/A	15	28517539	het	24	splice_region_variant, intron_variant	0				rs80340439	0
GALNT16	C>C/T	14	69787589	het	69	splice_region_variant, intron_variant	0				rs7147076	1
C14orf37	T>T/C	14	58478834	het	11	splice_region_variant, intron_variant	0				rs150566056	1
MIS18BP1	A>A/C	14	45700336	het	111	splice_region_variant, intron_variant	0				rs200622251	0.09
LOC1001322	C>C/T	14	19114436	het	80	splice_region_variant, intron_variant	0				rs200286503	0
SLC15A1	T>T/C	13	99354731	het	12	splice_region_variant, intron_variant	0				rs8187827	1
INTS6	A>A/G	13	51961679	het	69	splice_region_variant, intron_variant	0				rs138030207	1
ALOX5AP	G>G/A	13	31287981	het	108	splice_region_variant, intron_variant	0				rs73163047	0
ANAPC7	A>A/G	12	110819761	het	11	splice_region_variant, intron_variant	0				rs78217750	0
DDX11	T>T/C	12	31247682	het	137	splice_region_variant, intron_variant	0				rs61917225	0
GYS2	C>C/T	12	21695425	het	116	splice_region_variant, intron_variant	0					0
PTPRO	C>C/A	12	15699498	het	41	splice_region_variant, intron_variant	0				rs76233597	1
PITPNM1	G>G/A	11	67262701	het	31	splice_region_variant, intron_variant	0					0
DPP3	T>T/C	11	66259233	het	158	splice_region_variant, intron_variant	0					0
KIF18A	G>G/A	11	28116352	het	142	splice_region_variant, intron_variant	0				rs11607351	2
ABLIM1	A>A/C	10	116199837	het	60	splice_region_variant, intron_variant	0					0
COL13A1	C>C/T	10	71716753	het	117	splice_region_variant, intron_variant	0				rs78160146	1
BICC1	T>T/C	10	60556290	het	55	splice_region_variant, intron_variant	0				rs3740453	1
TIMM23	A>A/G	10	51620317	het	18	splice_region_variant, intron_variant	0				rs200147310	0
SVIL	G>G/A	10	29760178	het	35	splice_region_variant, intron_variant	0					0
ANKRD26	T>T/C	10	27335276	het	59	splice_region_variant, intron_variant	0					0

WDR5	A>A/T	9	137007451	het	35	splice_region_variant, intron_variant	0		rs28562287	2
LRSAM1	C>C/T	9	130258380	het	71	splice_region_variant, intron_variant	0		rs75171318	1
FKTN	A>A/G	9	108363420	het	6	splice_region_variant, intron_variant	0		rs41277795	0.32
MSANTD3,M	C>C/T	9	103204644	het	63	splice_region_variant, intron_variant	0		rs117582277	1
TEK	C>C/T	9	27209111	het	180	splice_region_variant, intron_variant	0			0
GSDMD	G>G/A	8	144642944	het	41	splice_region_variant, intron_variant	0		rs141790883	0.46
ZFAND1	G>G/A	8	82626146	het	29	splice_region_variant, intron_variant	0		rs75348453	2
RPL7	C>C/T	8	74205826	het	18	splice_region_variant, intron_variant	0		rs191975568	0.09
C8orf34	T>T/C	8	69699672	het	66	splice_region_variant, intron_variant	0		rs183203857	0.23
PCDHA1,PCD	C>C/T	5	140256508	het	75	intron_variant	0			0
LAMB1	G>G/A	7	107564539	het	27	splice_region_variant, intron_variant	0		rs3213673	0
PCDHA1,PCD	C>C/G	5	140188354	het	26	intron_variant	0		rs142480630	0
WDR55	G>G/C	5	140048941	het	123	downstream_gene_variant	0			0
SRA1	GGT>GGT/A	5	139931777	het	39	downstream_gene_variant	0			0
SRA1	C>C/GTCG	5	139931629	het	57	downstream_gene_variant	0			0
FAM185A	T>T/C	7	102412901	het	308	splice_region_variant, intron_variant	0		rs2539288	0
TMED7-TICAI	T>T/A	5	114952011	het	130	synonymous_variant	190	S	rs1058047	1
MUC3A	T>T/A	7	100553073	het	265	splice_region_variant, intron_variant	0		rs73714269	0
PON3	G>G/A	7	94991666	het	204	splice_region_variant, intron_variant	0			0
DGKB	GA>GA/C	7	14775823	het	25	splice_region_variant, intron_variant	0		rs72291444	0
RSPH10B	T>T/C	7	5998617	het	327	splice_region_variant, intron_variant	0		rs201508579	0
STX7	G>G/A	6	132791083	het	193	splice_region_variant, intron_variant	0		rs78468014	1
SEC63	AAAAAC>AAAAAC/GGGG	6	108243120	het	23	splice_region_variant, intron_variant	0			0
PKHD1	A>A/C	6	51712773	het	44	splice_region_variant, intron_variant	0			0
LYAR	T>T/G	4	4275302	het	46	splice_region_variant, intron_variant	0			0
MUC4	C>C/T	3	195490909	het	29	splice_region_variant, intron_variant	0		rs201624504	0
CD96	A>A/G	3	111366520	het	72	splice_region_variant, intron_variant	0			0
SPINK8	T>T/C	3	48351391	het	20	splice_region_variant, intron_variant	0			0
ZFYVE20	A>A/G	3	15119671	het	92	splice_region_variant, intron_variant	0		rs149381129	0.09
CAND2	C>C/T	3	12861579	het	160	splice_region_variant, intron_variant	0			0
ALPP	C>C/T	2	233245210	het	180	splice_region_variant, intron_variant	0		rs13034594	0
LRP2	C>C/T	2	170145661	het	109	splice_region_variant, intron_variant	0		rs144147038	0.46
WDR1	C>C/T	4	10079377	het	49	downstream_gene_variant	0		rs34193855	0.23
NEB	C>C/T	2	152536427	het	118	splice_region_variant, intron_variant	0		rs74859201	1
ANAPC1	A>A/C	2	112615875	het	104	splice_region_variant, intron_variant	0		rs200985332	0
10-Sep	A>A/C	2	110332350	het	30	splice_region_variant, intron_variant	0			0
MFI2	A>A/G	3	196736575	het	109	downstream_gene_variant	0		rs41284047	1
ANKRD36C	T>T/C	2	96604730	het	19	splice_region_variant, intron_variant	0		rs113311459	0
DNAH6	A>A/T	2	84924854	het	115	splice_region_variant, intron_variant	0			0
AFTPH	A>A/G	2	64796816	het	115	splice_region_variant, intron_variant	0		rs137907854	0.09
PAPOLG	T>T/A	2	61009813	het	7	splice_region_variant, intron_variant	0		rs74492547	0
CAPN13	T>T/C	2	30959366	het	104	splice_region_variant, intron_variant	0		rs11889549	2
LYST	G>G/T	1	235840495	het	55	splice_region_variant, intron_variant	0		rs72761794	0.18
SRGAP2	A>A/G	1	206579936	het	30	splice_region_variant, intron_variant	0		rs201644221	0
ARL8A	G>G/T	1	202104352	het	59	splice_region_variant, intron_variant	0		rs41310917	0
KIF21B	G>G/A	1	200944044	het	31	splice_region_variant, intron_variant	0		rs200282109	0.05
ACOT11,FAM	AG>AG/C	1	55085686	het	85	splice_region_variant, intron_variant	0			0
WAS	A>A/AC	X	48542816	het	143	splice_region_variant, intron_variant, feature_elongation	0		rs193922413	0
C20orf96	T>T/TTA	20	271225	het	37	splice_region_variant, intron_variant, feature_elongation	0			0
CD226	T>T/TA	18	67614674	het	19	splice_region_variant, intron_variant, feature_elongation	0		rs35995527, rs3	0
RIT2	G>G/GA	18	40503735	het	23	splice_region_variant, intron_variant, feature_elongation	0		rs201023157	0
HAP1	T>T/TG	17	39888648	het	53	splice_region_variant, intron_variant, feature_elongation	0			0
SLC28A1	A>A/AG	15	85488102	het	76	splice_region_variant, intron_variant, feature_elongation	0			0
CEP128	G>G/GA	14	80993330	het	18	splice_region_variant, intron_variant, feature_elongation	0		rs34838656, rs6	0
DIAPH3	A>A/ATTAC	13	60385060	het	14	splice_region_variant, intron_variant, feature_elongation	0		rs10665848	0
SKA3	T>T/TA	13	21729952	het	21	splice_region_variant, intron_variant, feature_elongation	0		rs11446085, rs5	0
VWA5B2	G>G/A	3	183959528	het	31	downstream_gene_variant	0			0
BBOX1	A>A/AG	11	27137108	het	347	splice_region_variant, intron_variant, feature_elongation	0		rs3214719, rs113	
IGSF10	C>C/T	3	151156366	het	251	downstream_gene_variant	0		rs141250073	0.05
NRAP	G>G/GAA	10	115405703	het	46	splice_region_variant, intron_variant, feature_elongation	0		rs35741231, rs10	
SYT15	T>T/TG	10	46965887	het	134	splice_region_variant, intron_variant, feature_elongation	0		rs112965082, rs	0
SETX	T>T/TA	9	135152543	het	79	splice_region_variant, intron_variant, feature_elongation	0		rs201575546	0
FAM184A	T>T/TA	6	119296378	het	20	splice_region_variant, intron_variant, feature_elongation	0		rs78125835, rs2	0
SEC63	T>T/TGGG	6	108243115	het	65	splice_region_variant, intron_variant, feature_elongation	0		rs5878948, rs14	0
CELSR3	C>C/T	3	48680470	het	85	downstream_gene_variant	0		rs112843572	1
CELSR3	G>G/A	3	48677191	het	191	downstream_gene_variant	0		rs61729242	1
SLC25A36	T>T/TA	3	140678384	het	21	splice_region_variant, intron_variant, feature_elongation	0			0
HACL1	T>T/TA	3	15613279	het	31	splice_region_variant, intron_variant, feature_elongation	0		rs200924343	0
TRMT1L	G>G/GA	1	185121070	het	34	splice_region_variant, intron_variant, feature_elongation	0			0
TAB3	TAAA>TAAA/T	X	30877801	het	8	splice_region_variant, intron_variant, feature_truncation	0		rs58473340	0
RTDR1	GA>GA/G	22	23481133	het	50	splice_region_variant, intron_variant, feature_truncation	0		rs10568631	0
TMEM43	A>A/G	3	14183204	het	69	downstream_gene_variant	0			0
SLC39A11	CAA>CAA/C	17	70845949	het	36	splice_region_variant, intron_variant, feature_truncation	0		rs34998299	0
MYO15A	AGT>AGT/A	17	18034131	het	55	splice_region_variant, intron_variant, feature_truncation	0			0
RBM26	TA>TA/T	13	79946071	het	19	splice_region_variant, intron_variant, feature_truncation	0			0
HSPH1	TA>TA/T	13	31722620	het	31	splice_region_variant, intron_variant, feature_truncation	0		rs35594388	0
POC1B	TAGAAAGAAGA>TAGAAAGAAGA/T	12	89866054	het	50	splice_region_variant, intron_variant, feature_truncation	0		rs59139895	0
LDHB	TA>TA/T	12	21791410	het	10	splice_region_variant, intron_variant, feature_truncation	0		rs71053332	0
ASPH	GA>GA/G	8	62555482	het	15	splice_region_variant, intron_variant, feature_truncation	0			0
EIF2AK1	GA>GA/G	7	6080853	het	31	splice_region_variant, intron_variant, feature_truncation	0		rs34743814	0
PMS2	GA>GA/G	7	6037057	het	34	splice_region_variant, intron_variant, feature_truncation	0		rs60794673	0
SYNE1	GA>GA/G	6	152629772	het	13	splice_region_variant, intron_variant, feature_truncation	0		rs55633181	0
KCTD20	TA>TA/T	6	36452603	het	36	splice_region_variant, intron_variant, feature_truncation	0		rs201092661	0
CDS1	GA>GA/G	4	85556511	het	47	splice_region_variant, intron_variant, feature_truncation	0			0
MUC20	AC>AC/A	3	195452663	het	44	splice_region_variant, intron_variant, feature_truncation	0			0
FGF12	GA>GA/G	3	191888452	het	29	splice_region_variant, intron_variant, feature_truncation	0		rs34265167	0
TBX19	AGT>AGT/A	1	168262522	het	72	splice_region_variant, intron_variant, feature_truncation	0			0
NBPF14,NOT	TC>TC/T	1	145281361	het	555	splice_region_variant, intron_variant, feature_truncation	0		rs147539840	0
TUBA3D	C>C/T	2	132237643	het	5	downstream_gene_variant	0		rs72992288	0
DDX20	TA>TA/T	1	112305406	het	82	splice_region_variant, intron_variant, feature_truncation	0		rs78248378	0
SYS1-DBNDD	C>C/T	20	44037095	het	97	splice_region_variant, intron_variant, nc_transcript_variant	0			0
FRG1B	A>A/T	20	29628335	het	170	splice_region_variant, intron_variant, nc_transcript_variant	0		rs11152455	0
CNNM4	C>C/T	2	97465384	het	29	downstream_gene_variant	0		rs41286594	1
AQP7P1	T>T/G	9	67281836	het	161	splice_region_variant, intron_variant, nc_transcript_variant	0		rs75812522	0
NBPF22P	T>T/C	5	85582712	het	56	splice_region_variant, intron_variant, nc_transcript_variant	0		rs2964837	0
SDHAP3	C>C/T	5	1572408	het	181	splice_region_variant, intron_variant, nc_transcript_variant	0			0
MST1L	C>C/G	1	17085671	het	96	splice_region_variant, intron_variant, nc_transcript_variant	0		rs3863806	0
MST1L	C>C/T	1	17084006	het	64	splice_region_variant, intron_variant, nc_transcript_variant	0		rs2446544	0
ESPNP	T>T/C	1	17046456	het	197	splice_region_variant, intron_variant, nc_transcript_variant	0		rs3856292	0
MST1P2	T>T/A	1	16975868	het	49	splice_region_variant, intron_variant, nc_transcript_variant	0		rs200822664	0
CROCCP2	T>T/C	1	16952267	het	15	splice_region_variant, intron_variant, nc_transcript_variant	0		rs1765552	0
CROCCP2	G>G/A	1	16946336	het	31	splice_region_variant, intron_variant, nc_transcript_variant	0		rs11586784	0
MST1L	A>A/AT	1	17090902	het	80	splice_region_variant, intron_variant, nc_transcript_variant, featu	0			0
FRG1B	CTATT>CTATT/C	20	29632726	het	66	splice_region_variant, intron_variant, nc_transcript_variant, featu	0		rs138922778	0
ZNF890P	GA>GA/G	7	5161858	het	35	splice_region_variant, intron_variant, nc_transcript_variant, featu	0		rs11289360	0
RNF103-CHM	C>C/A	2	86831051	het	131	intron_variant	0		rs61760874	0.41
DDX12P	A>A/T	12	9578197	het	268	splice_region_variant, non_coding_exon_variant, nc_transcript_va	0		rs76671194	0
MRPL53	G>G/T	2	74699595	het	168	downstream_gene_variant	0		rs78834087	1
PRSS3P2	C>C/T	7	142480067	het	71	splice_region_variant, non_coding_exon_variant, nc_transcript_va	0		rs201659809	0
DTX2P1-UPK	G>G/A	7	76629626	het	55	splice_region_variant, non_coding_exon_variant, nc_transcript_va	0		rs145082715	0
PLEKHH2,LO	C>C/G	2	43902757	het	156	intron_variant	0		rs143787127	2
MST1L	C>C/T	1	17083888	het	18	splice_region_variant, non_coding_exon_variant, nc_transcript_va	0		rs75141545	0
MST1P2	G>G/A	1	16976569	het	87	splice_region_variant, non_coding_exon_variant, nc_transcript_va	0		rs78288272	0
CROCCP2	C>C/G	1	16952993	het	160	splice_region_variant, non_coding_exon_variant, nc_transcript_va	0		rs942268	0
FOXP3	G>G/A	X	49113312	het	129	splice_region_variant, synonymous_variant	181	S	rs2232367	2
OR2L13,OR2L	G>G/T	1	248224569	het	493	intron_variant	0		rs75239130	0
OR2L13,OR2L	T>T/A	1	248224520	het	506	intron_variant	0			0
GATSL3	A>A/G	22	30682006	het	62	splice_region_variant, synonymous_variant	275	F	rs188064023	0.05
DGCR6	C>C/G	22	18893995	het	82	splice_region_variant, synonymous_variant	36	P	rs409155	3
KIR3DL1	C>C/T	19	55341556	het	154	splice_region_variant, synonymous_variant	387	D	rs1130472	1
SAMD1	C>C/G	19	14200891	het	6	splice_region_variant, synonymous_variant	114	P		0
SNAP47	C>C/G	1	227923184	het	47	downstream_gene_variant	0			0
RAB3GAP2	TA>TA/T	1	220369745	het	22	downstream_gene_variant	0		rs35396665	0
HYDIN	G>G/A	16	71019225	het	34	splice_region_variant, synonymous_variant	1398	L	rs201708128	0
SNX29	C>C/G	16	12223613	het	12	splice_region_variant, synonymous_variant	531	A	rs201093247	0.23
TMEM114	G>G/A	16	8620007	het	67	splice_region_variant, synonymous_variant	47	S	rs141472774	0.46
LRRC43	A>A/G	12	122677549	het	16	splice_region_variant, synonymous_variant	449	P		0
TTC40	C>C/G	10	134674286	het	12	splice_region_variant, synonymous_variant	1697	T	rs78376398	1
KMT2C	G>G/A	7	151896365	het	28	splice_region_variant, synonymous_variant	1424	H	rs200156200	0
SLC26A3	G>G/A	7	107420206	het	256	splice_region_variant, synonymous_variant	438	S	rs117703371	0.46
ZNF806	T>T/C	2	133074683	het	137	splice_region_variant, synonymous_variant	101	D	rs7355726	0
ANKRD36	G>G/A	2	97833342	het	21	splice_region_variant, synonymous_variant	520	T	rs5008284	0
ANKRD36C	C>C/T	2	96619687	het	130	splice_region_variant, synonymous_variant	367	E	rs199947548	0
ZNF135	C>C/T	19	58578804	het	102	stop_gained	342	R/*	rs200526463	0
LTBP4	G>G/A	19	41128415	het	55	stop_gained	1176	W/*	rs35079932	1
CDC27	G>G/A	17	45214558	het	78	stop_gained	631	R/*	rs77685276	0
KRT40	C>C/A	17	39135066	het	14	stop_gained	396	E/*	rs189066032	0
GALK2	C>C/T	15	49611854	het	44	stop_gained	341	R/*	rs144322038	0

TMTC1	C>C/T	12	29936472	het	24	stop_gained	71	W/*	rs112603144	0
LOC1009965	G>G/A	11	69472557	het	126	stop_gained	43	R/*	rs117640087	1
ENKUR,THNS	C>C/T	10	25312168	het	63	stop_gained	6	R/*	rs145170378	0
RP1L1	C>C/A	8	10465490	het	87	stop_gained	2040	E/*	rs201774530	0
MUC3A	C>C/T	7	100552738	het	384	stop_gained	1107	Q/*	rs79874934	0
POLM	C>C/T	7	44116182	het	80	stop_gained	254	W/*		0
STPG2	G>G/A	4	99027142	het	39	stop_gained	192	R/*	rs141732256	0
CHIT1	C>C/CAGACCATGGCCCCGCCCAGTCCCT	1	203186950	het	39	stop_gained, inframe_insertion	358	W/*GLGGAMVW	rs3831317, rs15	0
C15orf32	T>T/G	15	93043628	het	121	stop_lost	179	*/G		0
MAGT1	G>G/A	X	77150892	het	115	upstream_gene_variant	0		rs140854076	0.12
LOC644634	A>A/G	1	149676401	het	141	downstream_gene_variant	0		rs146138154	0
E2F1	C>C/T	20	32266134	het	18	upstream_gene_variant	0		rs35385772	2
NECAB3	G>G/A	20	32248092	het	80	upstream_gene_variant	0		rs201272547	0.09
NOP56	G>G/T	20	2634006	het	156	upstream_gene_variant	0			0
ADCK4	C>C/A	19	41197980	het	58	upstream_gene_variant	0			0
SLC12A4	C>C/T	16	67981697	het	32	upstream_gene_variant	0		rs117213038	1
EXOC3L1	G>G/C	16	67221444	het	78	upstream_gene_variant	0		rs34746889	1
SETD1A	G>G/C	16	30991965	het	145	upstream_gene_variant	0			0
HIRIP3	C>C/T	16	30004819	het	90	upstream_gene_variant	0		rs150030843	0.18
TPSB2	C>C/G	16	1279574	het	30	upstream_gene_variant	0		rs199887053	0
TPSB2	C>C/T	16	1278767	het	11	upstream_gene_variant	0		rs201725275	0
SZT2,HYI	T>T/C	1	43919081	het	57	3_prime_UTR_variant	0		rs142369206	1
CHTF18	C>C/T	16	842518	het	93	upstream_gene_variant	0		rs35387463	1
BMP8B,OXCT	C>C/T	1	40235448	het	102	intron_variant	0		rs150795467	0
BMP8B	C>C/T	1	40230421	het	49	downstream_gene_variant	0			0
XKR8	C>C/T	1	28286666	het	51	downstream_gene_variant	0		rs201643190	0
MSLN	G>G/A	16	818452	het	88	upstream_gene_variant	0		rs150425699	1
GOLGA8B	T>T/A	15	34825091	het	8	upstream_gene_variant	0		rs200439797	0
HYLS1,PUS3	G>G/T	11	125769536	het	118	upstream_gene_variant	0		rs78786765	1
KLC2	T>T/TGGGGC	11	66032712	het	29	upstream_gene_variant	0			0
SCT	G>G/A	11	626442	het	40	upstream_gene_variant	0			0
C11orf35	C>C/T	11	558232	het	82	upstream_gene_variant	0		rs117038322	0.05
C9orf173	A>A/G	9	140146324	het	109	upstream_gene_variant	0			0
ENTPD2	G>G/T	9	139944816	het	79	upstream_gene_variant	0			0
ENTPD2	C>C/G	9	139944736	het	36	upstream_gene_variant	0		rs4880083	0
PMPCA	C>C/A	9	139305140	het	144	upstream_gene_variant	0		rs149055087	1
IFNA10	G>G/T	9	21206743	het	41	upstream_gene_variant	0		rs112963053	0
CPSF1	G>G/A	8	145620764	het	27	upstream_gene_variant	0		rs11556134	2
MUC3A	C>C/A	7	100610069	het	100	upstream_gene_variant	0		rs10258821	0
MUC3A	G>G/A	7	100608884	het	94	upstream_gene_variant	0		rs73163797	0
MUC3A	G>G/A	7	100608375	het	211	upstream_gene_variant	0		rs75592954	0
MUC3A	A>A/C	7	100608370	het	214	upstream_gene_variant	0		rs73714276	0
ERV3-1	C>C/T	7	64451709	het	123	upstream_gene_variant	0		rs71534243	1
TARP	T>T/C	7	38299727	het	93	upstream_gene_variant	0		rs138027161	0
PCDHB12	G>G/C	5	140590325	het	11	upstream_gene_variant	0			0
MST1	G>G/A	3	49726070	het	49	upstream_gene_variant	0		rs62262686	0
MST1	T>T/C	3	49726028	het	60	upstream_gene_variant	0		rs62262685	0
CEBPZ	C>C/T	2	37454811	het	109	upstream_gene_variant	0		rs76819627	0.41
THBS3	T>T/G	1	155167457	het	48	upstream_gene_variant	0			0
SCNN1D	C>C/T	1	1222958	het	180	downstream_gene_variant	0		rs111819661	1
