# Supplementary material for: A Novel Role for CSRP1 in a Lebanese Family with Congenital Cardiac Defects
Source: Front Genet. 2017 Dec 18;8:217. doi: 10.3389/fgene.2017.00217 (PMC5741687; doi:10.3389/fgene.2017.00217)
Supplement: Supplementary file 3 [file Table3.PDF]

Supplementary Table 3: Known Polydactyly Genes Filtered In

Gene	Location	Syndrome	Type of polydactyly
PHS	7p13	Pallister-Hall	postaxial
SLOS	11q13.4	Smith-Lemli-Opitz	postaxial
GLI3	7p14.1		postaxial A/B Preaxial IV
BBS2 1-15	multiple	Bardet-Biedl	postaxial
DYNC2H1	11q22.3	Short rib-polydactyly	preaxial
PITX1	5q31.1		preaxial
SHH	7q36.3		Pre/post axial
GLI2	2q14.2		Pre/post axial
EVC2	4p16.2	Ellis-van Creveld	postaxial
MKKS	20p12.2	Bardet-Biedl	postaxial
BOFS	6p24.3	Branchiooculofacial	preaxial
HOXD13	2q31.1	Brachydactyly/ Syndactyly	synpolydactyly/brachydactylyl
TMEM67	8q22.1	Meckel/ Joubert/COACH	postaxial
HOXA13	7p15.2	Hand-foot-genital / Guttmacher	post / synpolydactyly
CC2D2A	4p15.32	Meckel/ Joubert	
RPGRIP1L	16q12.2	Meckel/ Joubert/COACH	postaxial
MKS2	11q12.2	Meckel	postaxial
ROR2	9q22.31	Robinow	brachy-syn-polydactyly
TWIST1	7p21.1	Robinow-soruf/saethre-chotzen	syndactyly / polydactyly
OFD1	Xp22.2	Joubert/oral-facial-digital/simpson-golabi	postaxial
HOXD10	2q31.1	Charcot-Marie-Tooth/vertical talus	
BMP4	14q22.2		postaxial
ALX4	11p11.2	Parietal foramina/frontonasal dysplasia	preaxial
PIK3CA	3q26.32	multiple	
BBS12	4q27	Bardet-Biedl	postaxial
CEP290	12q21.32	Bardet-Biedl/Meckel/Joubert/Leber/Senior loke	postaxial
MKS1	17q22	Meckel/Barrdet-Biedl	
SALL1	16q12.1	Townes-Brocks	preaxial
GDF6	8q22.1	Klippel-Feil/Microphthalmia	postaxial
TFAP2B	6p12.3	Char	
FGFR2	10q26.13	multiple	preaxial
ZFP57	6p22.1	transient neonatal diabetes mellitus	postaxial
IFT80	3q25.33	asphyxiating thoracic dystrophy	postaxial
NPHP3	3q22.1	Meckel/nephronophthisis/Renal-hepatic-pancre	postaxia l
RAB23	6p11.2	Carpenter	
TP63	3q28	multiple	
DHCR7	11q13.4	Smith-Lemli-Opitz	postaxial
TRIM32	9q33.1	Bardet-Biedl	
CDKN1C	11p15.4	Beckwith-Wiedemann/IMAGE	
LBR	1q42.12	HEM skeletal/Pelger-Huet/Reynolds	postaxial
EBP	Xp11.23	Chondrodysplasia punctata	postaxial
EFNB1	Xq13.1	Craniofrontonasal	preaxial (only in mouse model)
PAX	10q24.31	Papillorenal/isolated renal hypoplasia	
COL2A1	12q13.11	multiple	
TFAP2A	6p24.3	Branchiooculofacial	
PAPA4	7q22	postaxial polydactyly	postaxial
PAPA3	19p13.2		postaxial
PAPA2	13q21		postaxial
MIPOL1	14q13.3	Mirror-image polydactyly	postaxial
