# Supplementary material for: A Novel Role for CSRP1 in a Lebanese Family with Congenital Cardiac Defects
Source: Front Genet. 2017 Dec 18;8:217. doi: 10.3389/fgene.2017.00217 (PMC5741687; doi:10.3389/fgene.2017.00217)
Supplement: Supplementary file 4 [file Table4.PDF]

Supplementary Table 4: Variants inherited from the mother with a MAF < 5%

Gene	Variant	Chr	Coordinate	Genotype	Inherited From	Read Depth	Consequence	Protein Position	Amino Acids	Sift	PolyPhen	Allele Freq
AATK	G>G/A	17	79094875	het	mother	151	downstream_gene_variant	0				0
ELL3	C>C/T	15	44069096	het	mother	133	downstream_gene_variant	0				0
FAHD1,MEIO	C>C/A	16	1884292	het	mother	146	intron_variant	0				0
DNAH12	G>G/T	3	57494915	het	mother	39	missense_variant	165	P/Q	tolerated(0.57)	unknown(0)	1
DUOXA1	G>G/A	15	45412435	het	mother	139	missense_variant	213	T/M	deleterious(0.01)	probably_damaging(0.996)	0.32
PDE4D	ATCT>ATCT/A	5	58476466	het	mother	226	inframe_deletion, splice_region_variant	254	R/-			0
ETAA1	C>C/T	2	67631958	het	mother	84	missense_variant	715	P/L	tolerated(0.35)	possibly_damaging(0.44)	1
FAM134B	G>G/C	5	16477814	het	mother	72	missense_variant	319	F/L	deleterious(0)	probably_damaging(0.984)	0
FGFRL1	G>G/A	4	1016254	het	mother	64	missense_variant	115	V/I	tolerated(1)	benign(0.001)	0
GNAL	T>T/C	18	11753853	het	mother	117	missense_variant	178	I/T	tolerated(0.68)	benign(0.009)	0
GNL3L	G>G/T	X	54584923	het	mother	66	missense_variant	501	A/S	tolerated(0.74)	benign(0.019)	0.06
RPA3	TAGCAGCAGC>TAGCAGCAGC/T	7	7712941	het	mother	36	intron_variant, feature_truncation	0				0
KDM4E	A>A/G	11	94758981	het	mother	87	missense_variant	87	H/R	deleterious(0.05)	benign(0.016)	1
KIAA1377	G>G/A	11	101829006	het	mother	50	missense_variant	205	R/K	tolerated(0.83)	benign(0.001)	1
LATS2	G>G/A	13	21563311	het	mother	111	missense_variant	203	A/V	tolerated(0.53)	benign(0)	2
MORN1	G>G/A	1	2290143	het	mother	63	missense_variant	253	R/W	deleterious(0)	probably_damaging(0.996)	1
TTN-AS1,TTN	A>A/C	2	179401074	het	mother	179	intron_variant, nc_transcript_variant	0				0
MUS81	G>G/A	11	65632507	het	mother	86	missense_variant	431	R/H	tolerated(0.08)	probably_damaging(0.981)	1
VWA5B2	G>G/A	3	183959528	het	mother	31	downstream_gene_variant	0				0
XKR8	C>C/T	1	28286666	het	mother	51	downstream_gene_variant	0				0
ZNF695	C>C/T	1	247150739	het	mother	64	intron_variant, nc_transcript_variant	0				0
MVD	G>G/T	16	88718944	het	mother	67	missense_variant	398	P/T	tolerated(0.8)	benign(0.042)	0.05
NEK1	G>G/C	4	170359267	het	mother	83	missense_variant	911	Q/E	tolerated(0.24)	benign(0.004)	0.41
OGDHL	C>C/G	10	50952101	het	mother	66	missense_variant	600	M/I	tolerated(0.39)	benign(0)	1
OR5A1	C>C/T	11	59210646	het	mother	53	missense_variant	2	S/F	tolerated(0.06)	benign(0.001)	0
IGSF10	C>C/T	3	151156366	het	mother	251	downstream_gene_variant	0				0.05
PARP4	G>G/A	13	25021201	het	mother	59	missense_variant	1080	L/F	tolerated(0.1)	benign(0.028)	0
PDPR	C>C/T	16	70162749	het	mother	104	missense_variant	142	R/C	deleterious(0)	probably_damaging(1)	0
PLEKHG4B	G>G/A	5	140622	het	mother	67	missense_variant	67	R/Q	tolerated(0.62)	benign(0)	0
PPP1R15A	G>G/T	19	49377288	het	mother	46	missense_variant	266	E/D	tolerated(0.06)	probably_damaging(0.946)	0
PRG4	A>A/T	1	186275756	het	mother	97	missense_variant	302	E/V		unknown(0)	0
PSMA8	A>A/G	18	23731818	het	mother	45	missense_variant	82	I/V	tolerated(1)	benign(0)	0
PTCH2	C>C/T	1	45294013	het	mother	85	missense_variant	555	R/Q	tolerated(0.31)	benign(0.012)	0
IL17C	G>G/A	16	88706385	het	mother	107	downstream_gene_variant	0				0.23
ROS1	G>G/A	6	117708999	het	mother	105	missense_variant	653	S/F	deleterious(0)	probably_damaging(0.948)	0.32
RPAP1	G>G/A	15	41829178	het	mother	118	missense_variant	49	P/L	tolerated(0.35)	benign(0.001)	1
SHOX2	G>G/T	3	157816035	het	mother	114	missense_variant	283	H/Q	tolerated(0.22)	possibly_damaging(0.881)	0
SLC22A10	G>G/T	11	63064887	het	mother	139	missense_variant	207	G/C	deleterious(0)	probably_damaging(1)	1
SLC3A1	A>A/T	2	44531310	het	mother	113	missense_variant	389	S/C	deleterious(0.05)	possibly_damaging(0.795)	0
SMYD4	C>C/T	17	1690118	het	mother	94	missense_variant	624	G/R	tolerated(0.92)	benign(0)	0
SORCS1	T>T/C	10	108439488	het	mother	92	missense_variant	522	Y/C	tolerated(0.18)	probably_damaging(0.949)	0
SRMS	C>C/T	20	62173916	het	mother	89	missense_variant	222	V/M	tolerated(0.13)	benign(0.015)	1
TEKT4	C>C/T	2	95537568	het	mother	88	missense_variant	82	R/C	tolerated(0.05)	benign(0.101)	1
CELSR3	C>C/T	3	48680470	het	mother	85	downstream_gene_variant	0				1
TRPM5	G>G/C	11	2442361	het	mother	65	missense_variant	122	D/E	deleterious(0)	probably_damaging(0.993)	0
TRPS1	C>C/G	8	116631392	het	mother	122	missense_variant	311	R/S	deleterious(0)	probably_damaging(0.968)	0
UBE4B	G>G/C	1	10228220	het	mother	76	missense_variant	1075	Q/H	deleterious(0)	probably_damaging(0.999)	1
USP35	C>C/T	11	77911745	het	mother	67	missense_variant	363	S/L	tolerated(0.43)	unknown(0)	0.27
VEPH1	C>C/T	3	157099020	het	mother	46	missense_variant	351	R/H	deleterious(0)	probably_damaging(0.929)	0
WDR4	C>C/A	21	44299536	het	mother	41	missense_variant	24	A/S	deleterious(0.02)	benign(0.388)	0.05
ZBTB24	C>C/T	6	109787476	het	mother	119	missense_variant	558	D/N	tolerated(0.36)	benign(0.005)	0.23
ZC3H12C	A>A/C	11	110036365	het	mother	136	missense_variant	852	D/A	deleterious(0.04)	benign(0.269)	1
ZNF747	C>C/A	16	30545994	het	mother	76	missense_variant	3	D/Y	deleterious(0)	probably_damaging(0.951)	0
ZYG11A	A>A/G	1	53347210	het	mother	124	missense_variant	606	N/S	tolerated(0.6)	benign(0.002)	1
ABLIM1	A>A/C	10	116199837	het	mother	60	splice_region_variant, intron_variant	0				0
PKHD1	A>A/C	6	51712773	het	mother	44	splice_region_variant, intron_variant	0				0
RNF165	C>C/T	18	44013476	het	mother	23	splice_region_variant, intron_variant	0				0
RSPH10B	T>T/C	7	5998617	het	mother	327	splice_region_variant, intron_variant	0				0
SCNN1D	C>C/T	1	1222958	het	mother	180	downstream_gene_variant	0				1
SVIL	G>G/A	10	29760178	het	mother	35	splice_region_variant, intron_variant	0				0
RBBP8	A>A/G	18	20516929	het	mother	51	splice_region_variant, intron_variant	0				0.05
AFTPH	A>A/G	2	64796816	het	mother	115	splice_region_variant, intron_variant	0				0.09
PES1	C>C/A	22	30975119	het	mother	79	splice_region_variant, intron_variant	0				0.32
BICC1	T>T/C	10	60556290	het	mother	55	splice_region_variant, intron_variant	0				1
SZT2,HYI	T>T/C	1	43919081	het	mother	57	3_prime_UTR_variant	0				1
INTS6	A>A/G	13	51961679	het	mother	69	splice_region_variant, intron_variant	0				1
STX7	G>G/A	6	132791083	het	mother	193	splice_region_variant, intron_variant	0				1
UBASH3A	T>T/C	21	43846801	het	mother	89	splice_region_variant, intron_variant	0				2
UBASH3A	G>G/A	21	43846802	het	mother	89	splice_region_variant, intron_variant	0				2
ZFAND1	G>G/A	8	82626146	het	mother	29	splice_region_variant, intron_variant	0				2
CDS1	GA>GA/G	4	85556511	het	mother	47	splice_region_variant, intron_variant, feat	0				0
FGF12	GA>GA/G	3	191888452	het	mother	29	splice_region_variant, intron_variant, feat	0				0
KCTD20	TA>TA/T	6	36452603	het	mother	36	splice_region_variant, intron_variant, feat	0				0
GATSL3	A>A/G	22	30682006	het	mother	62	splice_region_variant, synonymous_varia	275	F			0.05
TTC40	C>C/G	10	134674286	het	mother	12	splice_region_variant, synonymous_varia	1697	T			1
C9orf173	A>A/G	9	140146324	het	mother	109	upstream_gene_variant	0				0
ENTPD2	G>G/T	9	139944816	het	mother	79	upstream_gene_variant	0				0
GOLGA8B	T>T/A	15	34825091	het	mother	8	upstream_gene_variant	0				0
SCT	G>G/A	11	626442	het	mother	40	upstream_gene_variant	0				0
SETD1A	G>G/C	16	30991965	het	mother	145	upstream_gene_variant	0				0
PLEKHH2,LOC	C>C/G	2	43902757	het	mother	156	intron_variant	0				2
RNF213	A>A/G	17	78357478	het	mother	76	intron_variant, nc_transcript_variant	0				2
SENP3	C>C/T	17	7468277	het	mother	111	downstream_gene_variant	0				2
C11orf35	C>C/T	11	558232	het	mother	82	upstream_gene_variant	0				0.05
EXOC3L1	G>G/C	16	67221444	het	mother	78	upstream_gene_variant	0				1
SLC12A4	C>C/T	16	67981697	het	mother	32	upstream_gene_variant	0				1
