# Supplementary material for: A Novel Role for CSRP1 in a Lebanese Family with Congenital Cardiac Defects
Source: Front Genet. 2017 Dec 18;8:217. doi: 10.3389/fgene.2017.00217 (PMC5741687; doi:10.3389/fgene.2017.00217)
Supplement: Supplementary file 5 [file DataSheet1.DOC]

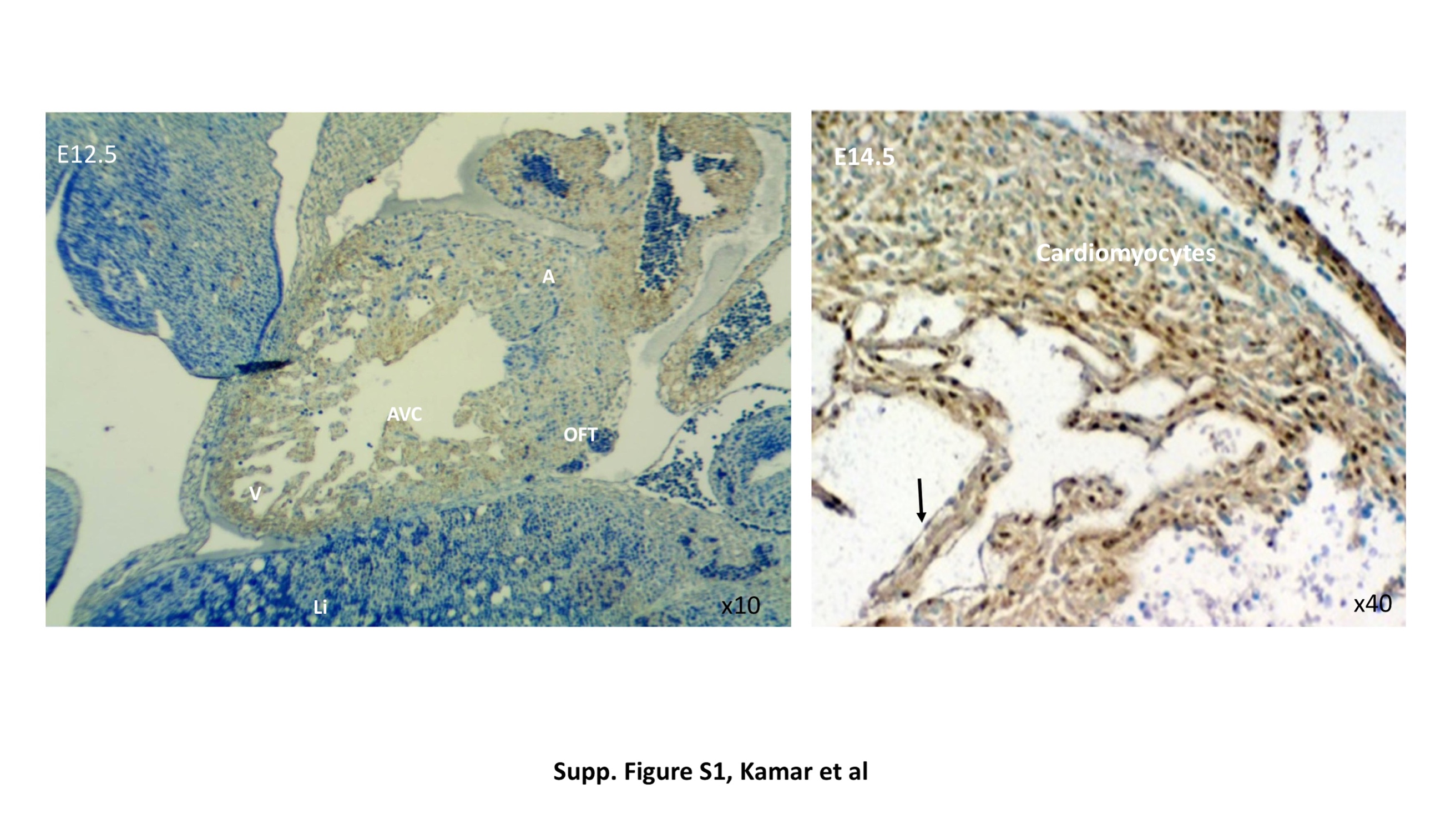


**Supp. Figure S1** Expression of CSRP1 during heart morphogenesis

Frontal sections of mouse embryos at different developmental stages (E12.5 and E14.5). CSRP1 shows a higher expression in nuclei (dark brown dots) of cardiomyocytes compared to the cytoplasm (light brown color) at embryonic stages. Nuclei were counter-stained with methyl green. Photos were taken at magnification 10x and 40x respectively. (A:aorta; OFT:outflowtract; v: ventricle; AVC: atrioventricular canal).


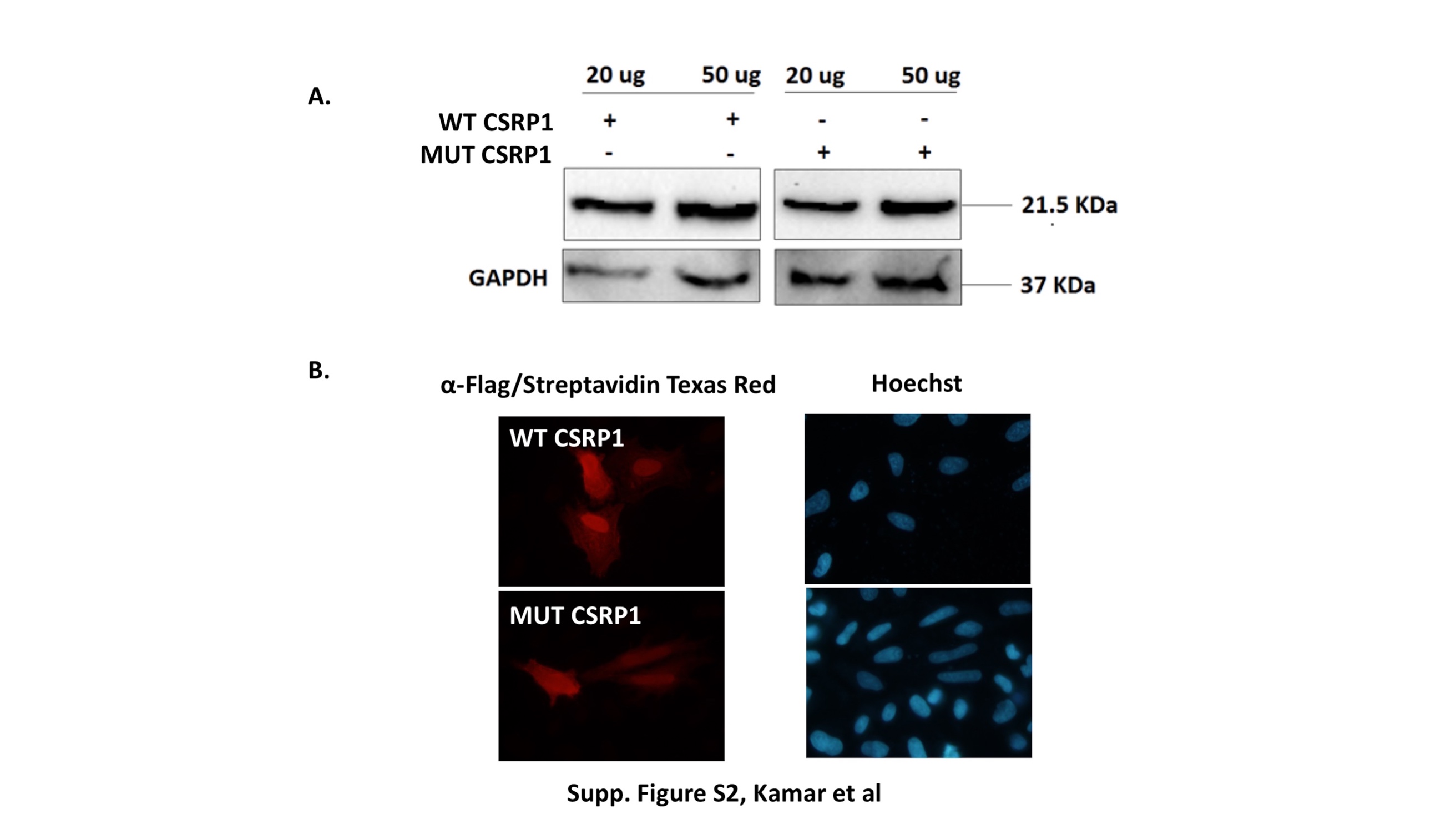


**Supp. Figure S2** Cellular localization of WT and MUT CSRP1 proteins

(**A**) Nuclear extracts from transfected HEK293 cells with either WT CSRP1 or MUT CSRP1 were resolved on an SDS-PAGE. Immunoblotting using anti-Flag antibody showed equal amounts of expressed proteins at 20 µg and 50 µg. Anti-GAPDH was used as a loading control. (**B**) Immunofluorescence of Hela cells transfected with 20 µg of WT CSRP1 and MUT CSRP1 plasmids. The localization of CSRP1 was visualized using anti-Flag antibody followed by biotinylated anti-mouse antibody and then fluorescent Streptavidin Texas Red. Nuclei were stained blue with the Hoechst 33342 dye. CSRP1 (WT or MUT) showed cytoplasmic and nuclear localization (red color).


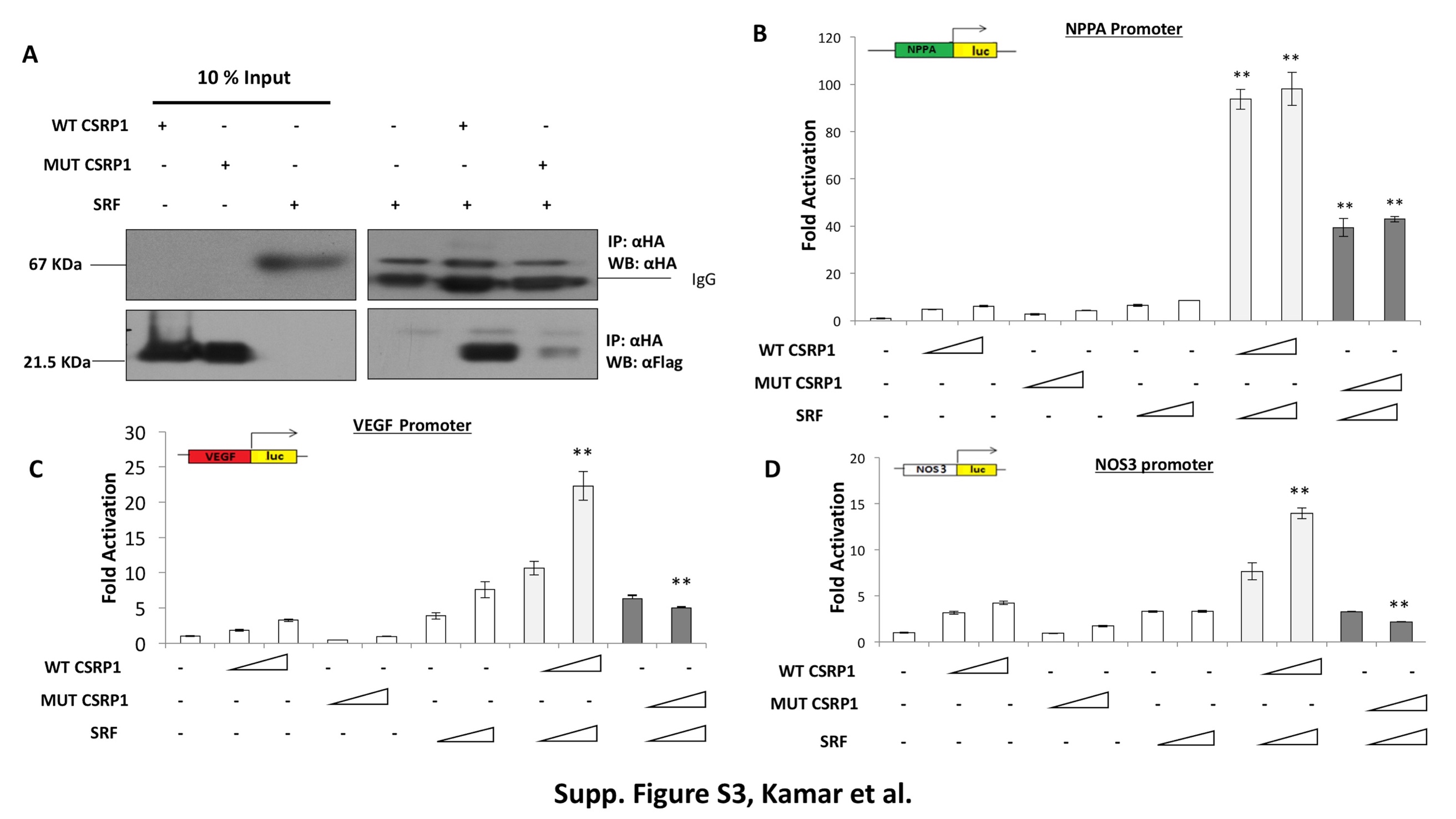


**Supp. Figure S3** The p.E154Vfs*99 mutation abolishes the physical and functional interaction between CSRP1 and SRF

**(A)**The amount of the proteins used for immunoprecipitation was ten times that used in western blot. Nuclear lysates of CSRP1 protein were immunoprecipitated with anti-HA antibody and CSRP1 proteins were detected using anti-Flag antibody (arrows). After membrane stripping, subsequent western blot analysis using anti-HA was performed to detect SRF protein (arrowheads) . (B,C,D)WT or MUT CSRP1 were transiently cotransfected with SRF along with 3.5 µg of NPPA, VEGF, and NOS3-luciferase promoters respectively in HEK293 cells. Media was changed 3 h post transfection and cells were harvested for luciferase assay after 36 h. Relative luciferase activities were presented as fold changes. The data represent the means of 3 independent experiments done in duplicates and the values are ± SE. P-value was assessed used Students’ T-test and significance p<0.01 is indicated by (**), while p<0.05 is indicated by an (*) ; significance of synergy for WT is tested relative to the sum of individual activations, while that of mutant is tested relative to synergy . The triangle represents an increasing dose of the WT and MUT CSRP1 ( 400 ng and 600 ng respectively) and SRF (200 and 400 ng).


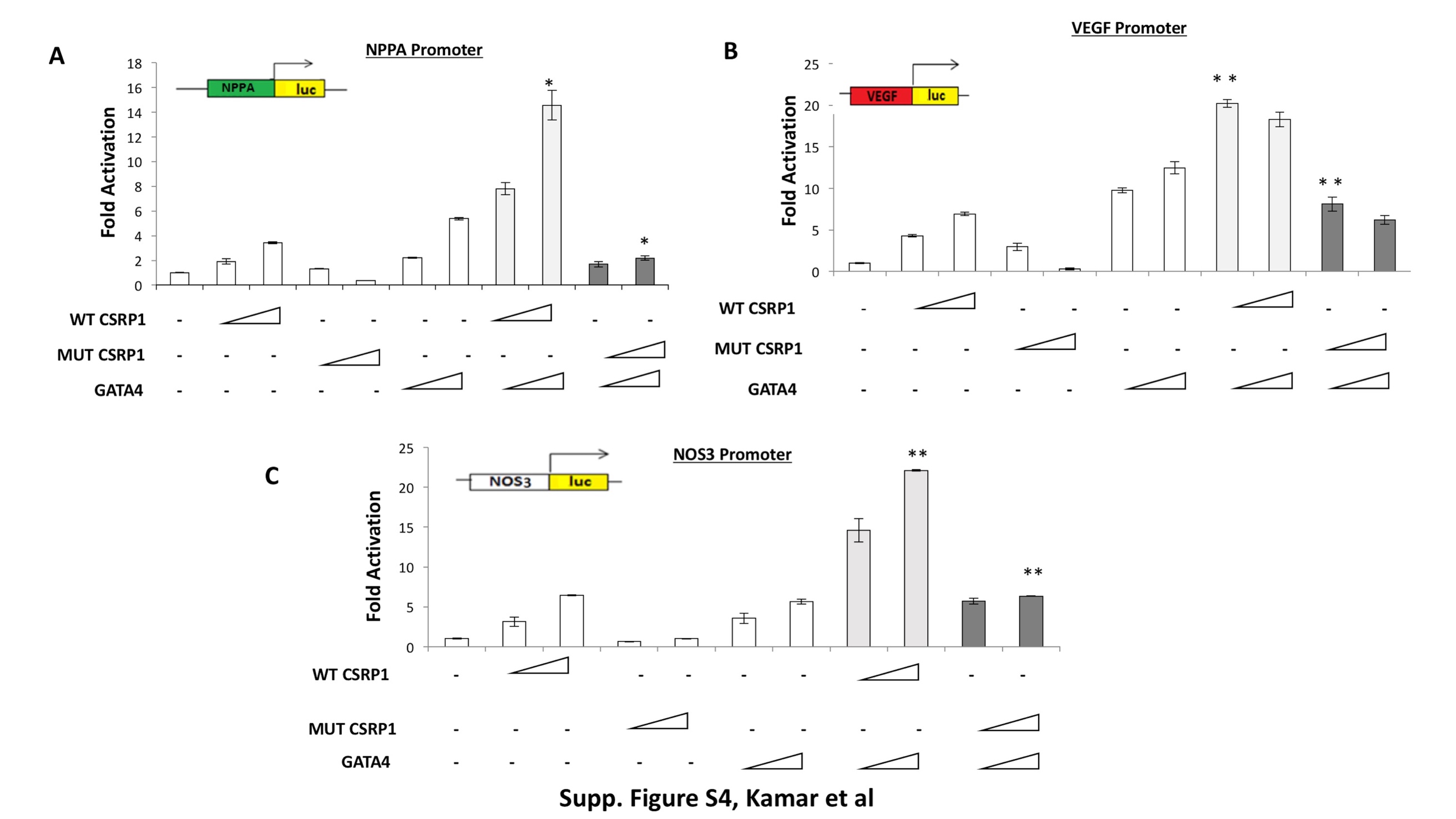


**Supp. Figure 4** Transcriptional activity of WT and/or MUT CSRP1 along with GATA4

(A,B,C) WT or MUT CSRP1 were transiently cotransfected with GATA4 along with NPPA, VEGF, and NOS3-luciferase promoters respectively in HEK293 cells. Media was changed 3 h post transfection and cells were harvested for luciferase assay after 36 h. Relative luciferase activities were presented as fold changes. WT CSRP1 synergistically activates the promoters when cotransfected with GATA4, however, MUT CSRP1 abolished this synergy. The data represent the means of 3 independent experiments done in duplicates and the values are ± SE. P-value was assessed used Students’ T-test. Significance p<0.01 is indicated by an (*) while p<0.05 is indicated by an (*); significance of synergy for WT is tested relative to the sum of individual activations, while that of mutant is tested relative to synergy. The triangle represents an increasing dose of the WT and MUT CSRP1 ( 400 ng and 600 ng respectively) and GATA4 (200 and 400 ng).
